# Cytokine-armed dendritic cell progenitors for antigen-agnostic cancer immunotherapy

**DOI:** 10.1038/s43018-023-00668-y

**Published:** 2023-11-23

**Authors:** Ali Ghasemi, Amaia Martinez-Usatorre, Luqing Li, Mehdi Hicham, Alan Guichard, Rachel Marcone, Nadine Fournier, Bruno Torchia, Darel Martinez Bedoya, Suzel Davanture, Mirian Fernández-Vaquero, Chaofan Fan, Jakob Janzen, Yahya Mohammadzadeh, Raphael Genolet, Nahal Mansouri, Mathias Wenes, Denis Migliorini, Mathias Heikenwalder, Michele De Palma

**Affiliations:** 1 Swiss Institute for Experimental Cancer Research (ISREC), School of Life Sciences, Swiss Federal Institute of Technology in Lausanne (EPFL), Lausanne, Switzerland; 2 Agora Cancer Research Center, Lausanne, Switzerland; 3 Swiss Cancer Center Léman (SCCL), Lausanne, Switzerland; 4 Translational Data Science (TDS) Facility, Swiss Institute of Bioinformatics (SIB), Lausanne, Switzerland; 5 Center for Translational Research in Onco-Hematology, University of Geneva (UNIGE), Geneva, Switzerland; 6 Division of Chronic Inflammation and Cancer, German Cancer Research Center (DKFZ), Heidelberg, Germany; 7 Ludwig Institute for Cancer Research, Lausanne, Switzerland; 8 Department of Oncology, University of Lausanne (UNIL) and Lausanne University Hospital (CHUV), Lausanne, Switzerland; 9 Department of Oncology, Geneva University Hospital (HUG), Geneva, Switzerland; 10 The M3 Research Center, Eberhard Karls University, Tübingen, Germany; 11 Cluster of Excellence iFIT (EXC 2180), Eberhard Karls University, Tübingen, Germany

**Keywords:** Tumour immunology, Cancer immunotherapy, Cancer

## Abstract

Dendritic cells (DCs) are antigen-presenting myeloid cells that regulate T cell activation, trafficking and function. Monocyte-derived DCs pulsed with tumor antigens have been tested extensively for therapeutic vaccination in cancer, with mixed clinical results. Here, we present a cell-therapy platform based on mouse or human DC progenitors (DCPs) engineered to produce two immunostimulatory cytokines, IL-12 and FLT3L. Cytokine-armed DCPs differentiated into conventional type-I DCs (cDC1) and suppressed tumor growth, including melanoma and autochthonous liver models, without the need for antigen loading or myeloablative host conditioning. Tumor response involved synergy between IL-12 and FLT3L and was associated with natural killer and T cell infiltration and activation, M1-like macrophage programming and ischemic tumor necrosis. Antitumor immunity was dependent on endogenous cDC1 expansion and interferon-γ signaling but did not require CD8^+^ T cell cytotoxicity. Cytokine-armed DCPs synergized effectively with anti-GD2 chimeric-antigen receptor (CAR) T cells in eradicating intracranial gliomas in mice, illustrating their potential in combination therapies.

## Abstract (web summary)

De Palma and colleagues develop a dendritic cell therapy based on dendritic cell progenitors engineered to produce IL-12 and FLT3L and show antigen-agnostic reduction of tumor burden that can be exploited for combination therapy in glioma.

## Main

DCs comprise developmentally distinct populations encompassing monocyte-derived DCs (moDCs), conventional DCs (cDCs) and plasmacytoid DCs. cDCs derive from rare bone-marrow (BM)-resident progenitors and can be further resolved into distinct subsets; among these, cDC1 have superior cross-presentation and cross-priming capabilities and are increasingly implicated in the orchestration of antitumor immunity^1–5^. DCs can present tumor antigens to T cells through distinct mechanisms^2,6^. One mechanism involves the engulfment of tumor-derived material followed by the presentation of major histocompatibility complex (MHC) class II (MHCII)-loaded peptides to CD4^+^ T helper cells, which in turn engage other immune cells such as macrophages and B cells. Alternatively, endocytosed tumor antigens may be loaded on MHC class I (MHCI), leading to cross-presentation of MHCI-loaded peptide complexes and cross-priming of naive CD8^+^ T cells. DCs can also acquire pre-formed MHC-loaded peptide complexes from cancer cells; for example, via extracellular vesicle or membrane transfer, a phenomenon referred to as cross-dressing^2,6,7^.

One clinical application, termed DC vaccine, involves isolating moDCs from a patient with cancer, exposing them to tumor antigens ex vivo (antigen loading) and maturing them before reinfusion into the patient^3,8–11^. Although moDC vaccines have shown promising clinical activity, therapeutic responses have been generally modest and inconsistent. Addressing the shortcomings of current moDC vaccines will be essential to improve the applicability of DC-based therapies^3,10,11^. One limitation of moDC-based therapies is the requirement for antigen loading and maturation, which limits DC lifespan, migration and antigen-presentation capacity in the recipient^2^. Additionally, the need for prior knowledge or availability of patient’s relevant tumor antigens poses challenges in the face of inter-patient and intra-patient tumor heterogeneity^3,10,12,13^. To address these limitations, we have developed a DC platform based on engineered DCPs that efficiently generate professional cDC1 and promote antitumor immunity without the need for antigen loading ex vivo.

## Results

### DCPs generate cDC1 in mice without prior host conditioning

**Fig. 1:**
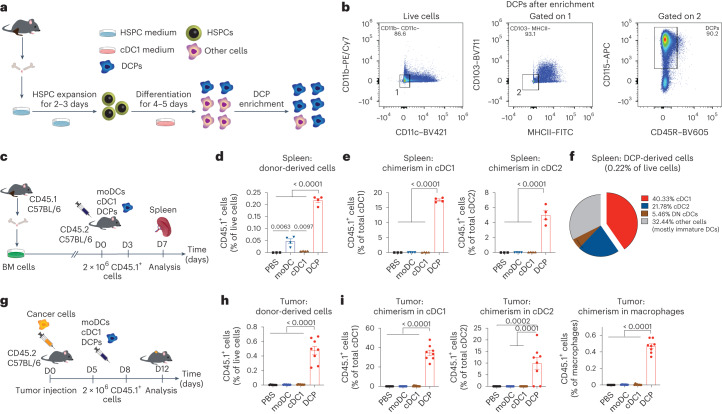
DCPs efficiently generate cDCs in mice. **a**, Procedure to generate DCPs from the mouse BM cells. **b**, Phenotype of DCPs after enrichment of lineage-negative cells. **c**, Procedure to study the fate of adoptively transferred DCPs, moDCs or cDC1-like cells in tumor-free mice. All DC types were generated from the BM of CD45.1 mice and transferred to CD45.2 mice. **d**, Engraftment of CD45.1^+^ cells derived from DCPs, moDCs and cDC1-like cells (mean ± s.e.m.; *n* = 3 mice for PBS and *n* = 4 for DCPs, moDCs and cDC1-like cells) in the spleen of recipient mice, 4 days after the last cell dose. Statistical analysis by one-way ANOVA with Tukey’s multiple comparison test. **e**, Donor cell chimerism in cDC1 and cDC2 (mean ± s.e.m.; *n* = 3 mice for PBS and *n* = 4 for DCPs, moDCs and cDC1-like cells). Statistical analysis by one-way ANOVA with Tukey’s multiple comparison test. **f**, Pie chart showing the fate of DCPs in the spleen (mean values; *n* = 4 mice). Double-negative (DN) DCs are defined as CD8a^–^ CD11b^–^ cDCs. Other cells mostly comprise CD11c^+^ MHCII^–/low^ immature DCs. **g**, Procedure to study the fate of DCPs in MC38 tumor-bearing mice. **h**, Engraftment of CD45.1^+^ cells derived from DCPs, moDCs and cDC1-like cells (mean ± s.e.m.; *n* = 7 mice for PBS and *n* = 8 for DCPs, moDCs and cDC1-like cells) in the tumor of recipient mice, 4 days after the last cell dose. Statistical analysis by one-way ANOVA with Tukey’s multiple comparison test. **i**, Donor cell chimerism in cDC1, cDC2 and macrophages in the tumor of recipient mice (mean ± s.e.m.; *n* = 7 mice for PBS and *n* = 8 for DCPs, moDCs and cDC1-like cells). Statistical analysis by one-way ANOVA with Tukey’s multiple comparison test. Each data point represents one sample from an independent mouse. Source data

**Extended Data Fig. 1:**
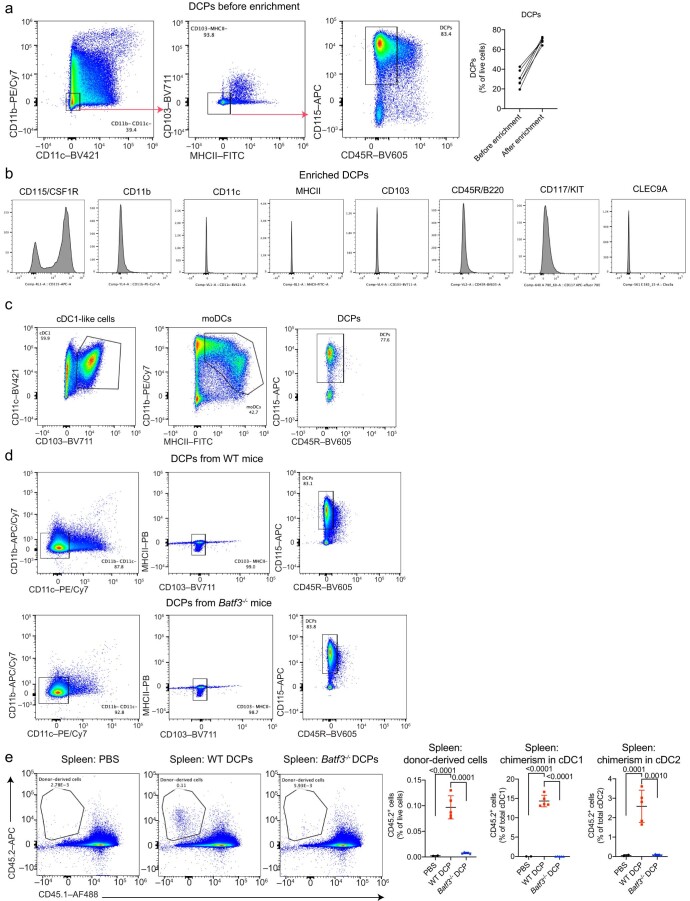
DCPs efficiently generate cDCs in recipient mice. **a**, Left: Phenotype of DCPs before enrichment of lineage-negative cells. Right: Proportion of DCPs before and after enrichment (paired samples) in several independent experiments (*n* = 6). **b**, Flow cytometry analysis of enriched DCPs from a representative experiment (gated on live cells). **c**, Flow cytometry dot plots showing the phenotype of cDC1-like cells, moDCs, and enriched DCPs, in one representative experiment. **d**, Flow cytometry dot plots showing the phenotype of enriched DCPs from representative wild-type (WT; top panels) or *Batf3*^–/–^ (bottom panels) mice. **e**, Left: Engraftment of CD45.2^+^ cells derived from WT or *Batf3*^–/–^ DCPs in the spleen of representative CD45.1 recipient mice. Right: Quantification of donor-derived cells in the spleen of recipient mice (mean ± SEM; *n* = *2* mice for PBS and *n* = *5* for WT and *Batf3*^–/–^ DCPs). Statistical analysis by one-way ANOVA with Tukey’s multiple comparison test. Each data point represents one sample from an independent cell culture or mouse. Source data

BM-resident, common DC progenitors (CDPs) are rare, prospective precursors of cDC1 in both mouse and human systems^3^. To obtain a cell population capable of generating cDC1, we developed a protocol for the ex vivo production of CDP-like cells from mouse BM (Fig. 1a). The two-step procedure involves short-term expansion of hematopoietic stem and progenitor cells (HSPCs) followed by partial differentiation under conditions that promote cDC1 lineage commitment^14^. BM cells were cultured for 2–3 days in ‘HSPC medium’ containing stem cell factor (SCF), thrombopoietin (TPO), FMS-related tyrosine kinase 3 ligand (FLT3L), IL-3, IL-6 and IL-1β (expansion phase). Floating cells were then cultured for four to five additional days in ‘cDC1 medium’ containing granulocyte-macrophage colony-stimulating factor (GM–CSF/CSF2) and FLT3L (differentiation phase). The resulting cell culture contained CDP-like cells (CD115^+^, CD11b^−^, CD11c^−^, CD103^−^, MHCII^−^, CD45R/B220^−^, CD117/KIT^−/low^ and CLEC9A^−^), which could be enriched from about 30% to 70% after depletion of lineage-positive cells (Fig. 1b and Extended Data Fig. 1a,b). At variance with BM-resident CDPs, the cultured CDP-like cells did not express CLEC9A; moreover, they lacked CD11c and CD103, which are expressed in pre-cDC1 and mature cDC1 (refs. ^3,14^). Owing to their similarity but non-identity with naturally occurring CDPs, we termed these cells DCPs.

We then asked whether DCPs could generate cDC1 in mice. We used BM cells of CD45.1 mice to produce DCPs as described above, or moDCs and mature cDC1-like cells (Extended Data Fig. 1c) using established protocols^7,14,15^. We inoculated each DC type intravenously (two doses of 2 × 10^6^ cells 3 days apart) in congenic CD45.2 mice, without prior myeloablation, and analyzed the spleen of recipient mice 4 days after the second DC dose (Fig. 1c). Gating strategies used to identify cell populations by flow cytometry in this and subsequent experiments are shown in Supplementary Figs. 1–6. Mice that received DCPs had a higher frequency of donor-derived CD45.1^+^ cells among splenocytes than mice that received moDCs or cDC1-like cells (Fig. 1d). We then examined splenic cDCs according to earlier work^16^ and found substantial donor chimerism within cDC1 (CD11c^+^CD11b^−^MHCII^+^CD8a^+^; >15%) and, to a lesser extent, cDC2 (CD11c^+^CD11b^+^MHCII^+^CD8a^−^) in mice that received DCPs (Fig. 1e). Conversely, donor cDC chimerism was negligible in mice that received moDCs or mature cDC1-like cells (<0.2%). The majority of DCP-derived cells were cDC1, cDC2 and double-negative (CD11b^−^CD8a^−^) cDCs (Fig. 1f). We also investigated whether BATF3, a transcription factor crucial for cDC1 development^17^, was necessary for generating DCPs ex vivo and for their engraftment and differentiation upon transfer to recipient mice. DCPs could be successfully established from the BM of CD45.2 *Batf3*^−/−^ mice but failed to engraft upon transfer to CD45.1 mice (Extended Data Fig. 1d,e).

**Extended Data Fig. 2:**
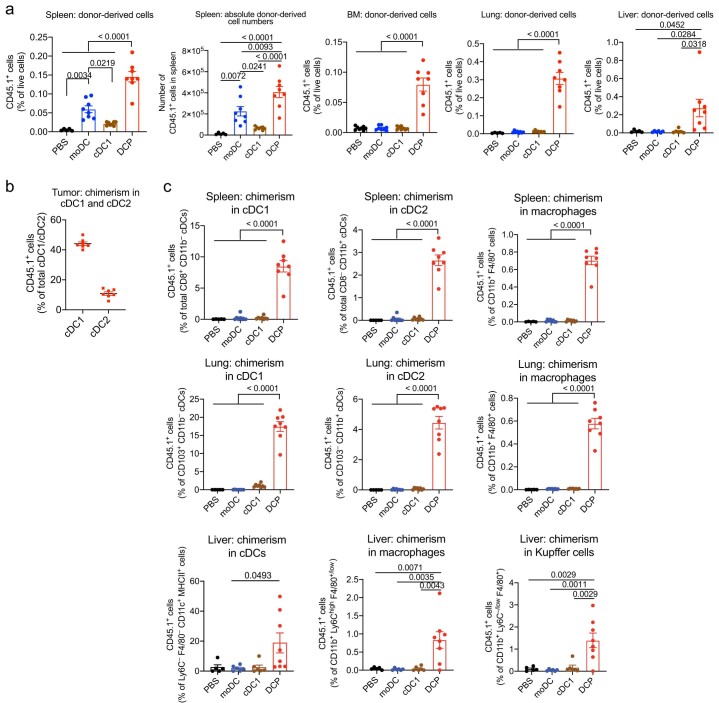
DCPs efficiently generate cDCs in distinct organs of recipient mice. **a**, Engraftment of CD45.1^+^ cells derived from DCPs, moDCs and cDC1-like cells (mean ± SEM; *n* = *5* mice for PBS and *n* = *8* for DCPs, moDCs and cDC1-like cells) in spleen (shown as relative frequency, left, and absolute cell counts, right), BM (mean ± SEM; *n* = *8* mice), lung (mean ± SEM; *n* = *5* mice for PBS and *n* = *8* for DCPs, moDCs and cDC1-like cells) and liver (mean ± SEM; *n* = *5* mice for PBS, *n* = *6* for moDCs and cDC1-like cells, and *n* = *8* for DCPs) of MC38 tumor-bearing mice, 4 days after the last cell dose. Statistical analysis by one-way ANOVA with Tukey’s multiple comparison test. **b**, Donor cell chimerism in cDC1 and cDC2 of MC38 tumors after DCP transfer in an independent experiment (mean ± SEM; *n* = *7* mice). **c**, Donor cell chimerism in cDC1, cDC2, cDCs, macrophages, or Kupffer cells of spleen (mean ± SEM; *n* = *5* mice for PBS and *n* = *8* for DCPs, moDCs and cDC1-like cells), lung (mean ± SEM; *n* = *5* mice for PBS and *n* = *8* for DCPs, moDCs and cDC1-like cells) and liver (mean ± SEM; *n* = *5* mice for PBS, *n* = *6* for moDCs and cDC1-like cells, and *n* = *8* for DCPs). Note that liver-derived cells display some autofluorescence, which gives background signal in the CD45.1 channel. Statistical analysis by one-way ANOVA with Tukey’s multiple comparison test. Each data point represents one sample from an independent mouse. Source data

We next studied the fate of donor DCPs, moDCs and cDC1-like cells in mice with subcutaneous MC38 colorectal tumors (Fig. 1g). CD45.1^+^ DCP-derived cells engrafted in tumor and other organs more efficiently than other DC populations (Fig. 1h and Extended Data Fig. 2a). In tumors, we identified cDC1 as CD103^+^CD11b^−^ cells and cDC2 as CD103^−^CD11b^+^ cells within the Ly6C^−^F4/80^−^CD11c^+^MHCII^+^ population, following previous work^18^. In independent experiments, DCP-derived cells accounted for approximately 35–45% and 10% of the tumor-associated cDC1 and cDC2, respectively (Fig. 1i and Extended Data Fig. 2b). DCPs made minimal contributions to non-cDC populations, such as macrophages (<1%), and robustly differentiated into cDCs also in spleen, lung and liver, while moDCs had low cDC differentiation capacity (Extended Data Fig. 2c). In summary, adoptively transferred DCPs efficiently reconstitute cDC1 and, to a lesser extent, cDC2 in tumor-bearing mice without the need for prior host conditioning.

### IL-12 promotes DCP differentiation into co-stimulatory cDC1

**Extended Data Fig. 3:**
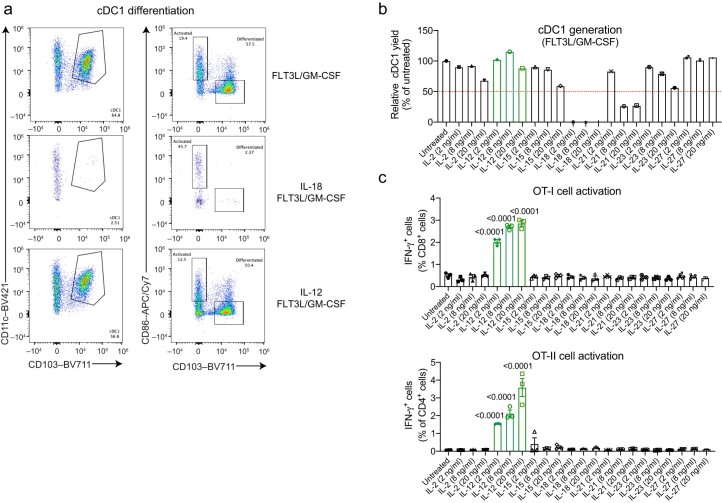
IL-12 and FLT3L promote differentiation of DCPs into cDC1 with enhanced co-stimulatory capacity. **a**, Differentiation of DCPs in cDC1 medium (containing FLT3L/GM-CSF) supplemented with the indicated cytokines, analyzed at day 15. Data show representative flow cytometry dot plots of DCPs stimulated with IL-18 or IL-12. **b**, Differentiation of DCPs into cDC1 in cDC1 medium (FLT3L/GM-CSF) supplemented with the indicated cytokines, shown as cDC1 yield relative to untreated. One sample per condition is shown. **c**, Activation, measured by intracellular IFNγ staining, of OT-I or OT-II cells in co-culture with cDC1-like cells pre-loaded with recombinant OVA protein (mean ± SEM; *n* = *3* independent cell cultures). Statistical analysis by one-way ANOVA with Tukey’s multiple comparison test. Each dot represents one independent cell culture. Source data

We then tested the effects of a panel of cytokines on the differentiation and co-stimulatory capacity of DCP-derived cDC1-like cells. We cultured DCPs in cDC1 medium (containing FLT3L and GM–CSF) supplemented with various cytokines (IL-2, IL-12, IL-15, IL-18, IL-21, IL-23 or IL-27) and analyzed the cells after 15 days. Both IL-18 and IL-21 compromised DCP differentiation into cDC1-like cells, as shown by reduced proportions of CD103^+^CD86^−^ cells, while simultaneously inducing premature DCP activation, as evidenced by increased proportions of CD86^+^CD103^−^ cells (Extended Data Fig. 3a,b). Conversely, IL-12 allowed for unperturbed cDC1-like cell differentiation. To study T cell co-stimulation, we co-cultured ovalbumin (OVA)-loaded, cDC1-like cells with OVA-specific CD8^+^ (OT-I) or CD4^+^ (OT-II) T cells in the presence of the aforementioned cytokines. Among the cytokines tested, only IL-12 induced robust interferon-γ (IFNγ) production by both OT-I and OT-II cells (Extended Data Fig. 3C). Thus, IL-12 preserves the cDC1-differentiation capacity of DCPs while enhancing the co-stimulatory capacity of cDC1-like cells.

### Cytokine-armed DCPs suppress melanoma growth

**Extended Data Fig. 4:**
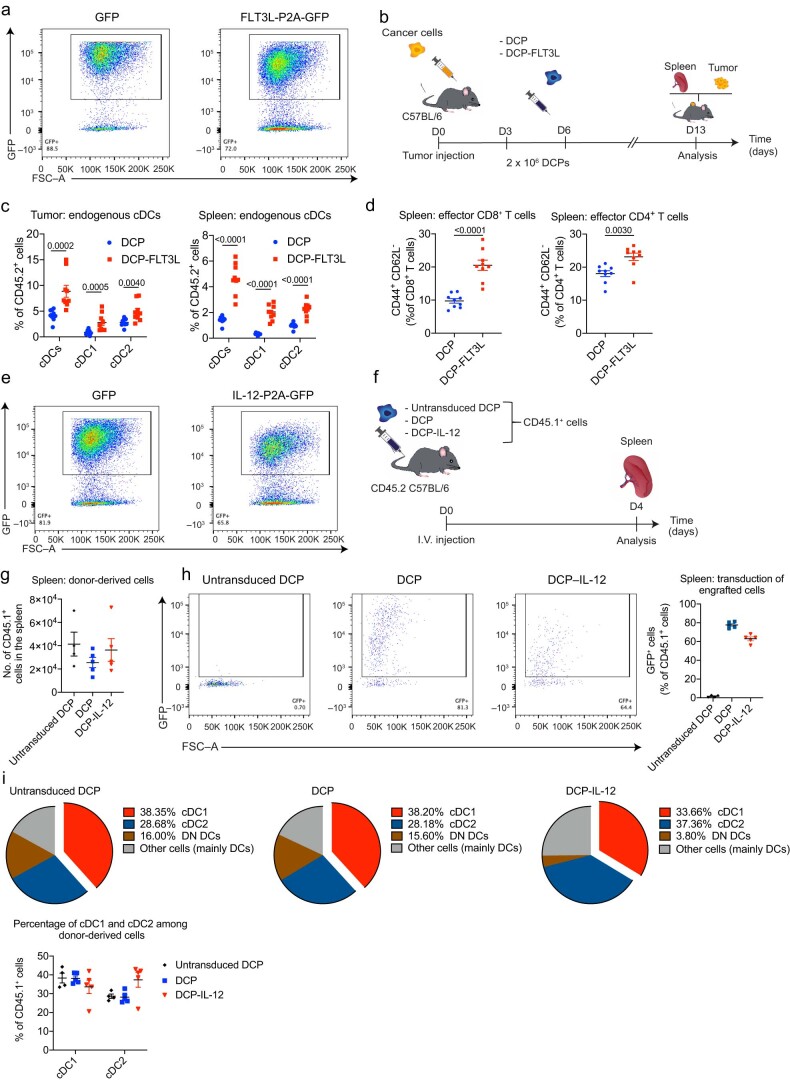
IL-12/FLT3L-expressing DCPs repopulate the cDC compartment of mice. **a**, Expression of GFP in LV-transduced DCPs analyzed 6 days post-transduction. **b**, Schematic of the experimental procedure to study adoptively transferred DCPs in B16F10 tumor-bearing mice. DCPs only express GFP; DCP-FLT3L co-express FLT3L and GFP. **c**, Frequency of the indicated cell types in tumor and spleen of mice that received DCPs (mean ± SEM; *n* = *9* mice). Statistical analysis by two-tailed Mann-Whitney test, performed independently on each cell type. **d**, Frequency of CD8^+^ and CD4^+^ cells with CD44^+^CD62^–^ T effector cell phenotype in spleen (mean ± SEM; *n* = *9* mice). Statistical analysis by two-tailed Mann-Whitney test. **e**, Expression of GFP in LV-transduced DCPs analyzed 6 days post-transduction. **f**, Schematic of the experimental procedure to study the fate of untransduced DCPs, DCPs only expressing GFP (DCP) and DCPs co-expressing IL-12 and GFP (DCP-IL-12) in tumor-free mice. A single dose of 2 × 10^6^ CD45.1^+^ DCPs was infused in CD45.2^+^ mice, and splenocytes were analyzed 4 days later. **g**, Number of CD45.1^+^ donor-derived cells in the spleen (untransduced DCP, *n* = *4* mice; DCP, *n* = *5*; DCP-IL12, *n* = *5*) of tumor-free mice. **h**, Left: Representative flow cytometry dot plots showing the frequency of transduced (GFP^+^) donor-derived cells in the spleen of tumor-free mice. One representative mouse per condition is shown. Right: Frequency of transduced (GFP^+^) donor-derived cells in the spleen (mean ± SEM; untransduced DCP, *n* = *4* mice; DCP, *n* = *5*; DCP-IL12, *n* = *5*). **i**, Top: Pie charts showing the fate of DCP-derived CD45.1^+^ cells in the spleen of tumor-free mice (mean values; untransduced DCP, *n* = *4* mice; DCP, *n* = *5*; DCP-IL12, *n* = *5*). Bottom: Quantification of the data showing the relative frequency (mean ± SEM) of cDC1 and cDC2 among CD45.1^+^ donor-derived cells. Each data point represents one sample from an independent mouse. Source data

FLT3L is a pivotal cytokine for cDC1 induction and expansion^18,19^. We reasoned that enforced expression of FLT3L in DCP-derived cells would expand endogenous cDC1 in tumors. To this aim, we generated a lentiviral vector (LV) expressing murine FLT3L together with green fluorescent protein (GFP); as a control, we used an LV expressing GFP only. We transduced BM-derived HSPCs on day two and then measured GFP expression and FLT3L secretion. Transduced cells efficiently expressed GFP on day six after transduction (Extended Data Fig. 4a) and robustly secreted FLT3L, as shown by enzyme-linked immunosorbent assay (ELISA) of medium conditioned by cells cultured for seven additional days in the absence of exogenous FLT3L (Supplementary Fig. 7a).

We then produced control DCPs (DCPs expressing GFP) and DCPs expressing FLT3L and GFP (hereafter DCP-FLT3L) from the BM of CD45.1 mice, using the protocol shown in Fig. 1a. In this and subsequent DCP transfer experiments, LV transduction was performed 2 h after DCP enrichment. We inoculated enriched DCPs or DCP-FLT3L in CD45.2 mice carrying subcutaneous B16F10 melanoma (Extended Data Fig. 4b). DCP-FLT3L effectively expanded endogenous cDCs in both tumor and spleen compared to control DCPs (Extended Data Fig. 4c). Moreover, DCP-FLT3L increased CD8^+^ and CD4^+^ T effector (CD44^+^CD62L^−^) cells (Extended Data Fig. 4d). Thus, FLT3L-armed DCPs may initiate antitumor immunity in mice by expanding endogenous cDCs and T effector cells.

IL-12 is a key cytokine for T cell activation^20^. Given that IL-12 improved the T cell co-stimulatory capacity of DCP-derived cDC1, we reasoned that transgenic expression of IL-12 by DCPs would enhance antitumor immunity initiated by DPC-FLT3L. We transduced BM-derived HSPCs on day two to generate DCP-IL-12 (DCPs expressing IL-12 and GFP) and control DCPs (DCPs expressing GFP only). Transduced cells robustly expressed GFP on day six after transduction (Extended Data Fig. 4e) and secreted IL-12, as shown by ELISA (Supplementary Fig. 7b). To study engraftment, we transduced enriched CD45.1 DCPs and inoculated 2 × 10^6^ cells in tumor-free CD45.2 mice (Extended Data Fig. 4f). DCP-IL-12 were not counter-selected, retained transgene expression (Extended Data Fig. 4g,h) and produced mature cDCs at the expected frequency in the spleen of recipient mice (Extended Data Fig. 4i).

**Fig. 2:**
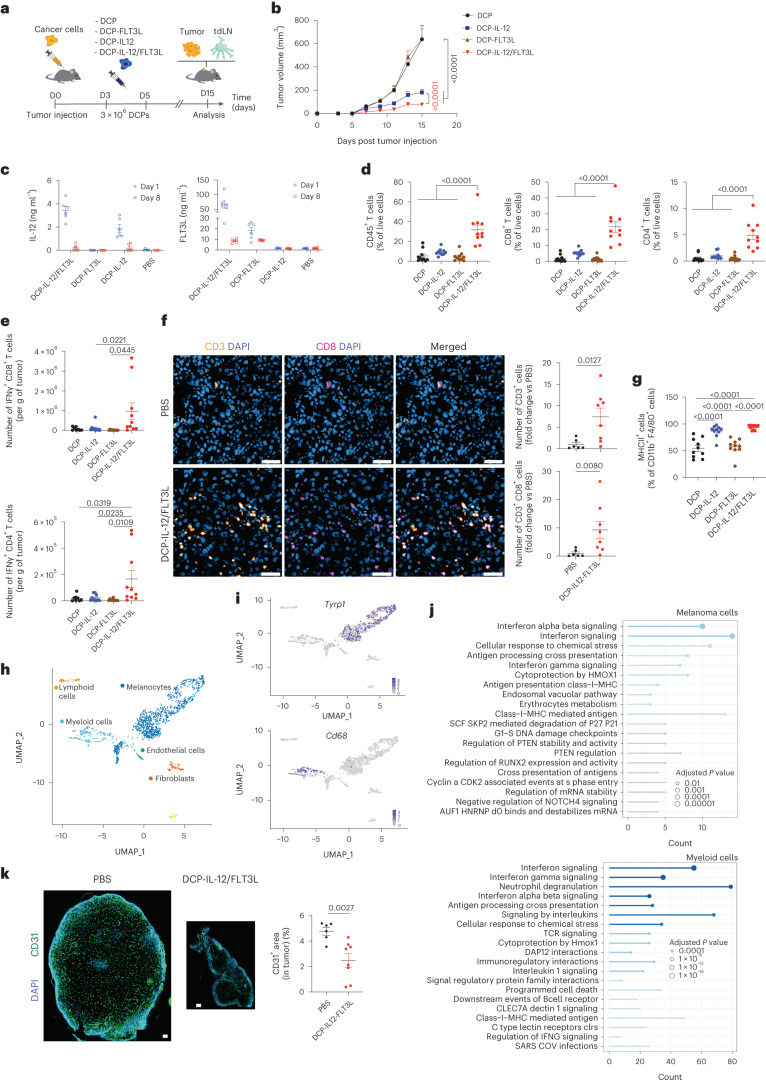
Cytokine-armed DCPs activate immunity and inhibit melanoma growth. **a**, Procedure to study transfer of cytokine-armed DCPs in B16F10 tumor-bearing mice. **b**, B16F10 tumor growth (mean  ± s.e.m.; *n* = 10 mice). Statistical analysis by two-way ANOVA with Tukey’s multiple comparison test; red *P* value was calculated by two-way ANOVA with Sidak’s multiple comparison test on DCP-IL-12 versus DCP-IL-12/FLT3L. **c**, Concentration of IL-12 and FLT3L in serum (mean ± s.e.m.; PBS, *n* = 5 mice; other groups, *n* = 6) of mice shown in Extended Data Fig. 5b, analyzed by ELISA at the indicated time points after the last DCP infusion. **d**, Frequency of the indicated cell types in tumors (mean ± s.e.m.; *n* = 10 mice). Statistical analysis by one-way ANOVA with Tukey’s multiple comparison test. **e**, IFNγ expression by ex vivo re-stimulated CD8^+^ and CD4^+^ T cells (mean ± s.e.m.; *n* = 10 mice in all groups, except for DCP where *n* = 8). Statistical analysis by one-way ANOVA with Tukey’s multiple comparison test. **f**, Left: representative images of CD3 (yellow) and CD8 (magenta) immunostaining, and DAPI nuclear staining (blue), of tumors from mice treated as indicated. Scale bar, 50 μm. Right: quantification of the data (mean ± s.e.m.; *n* = 6 mice for PBS and *n* = 8 for DCP-IL-12/FLT3L). Statistical analysis by two-tailed Mann–Whitney test. **g**, Frequency of M1-like TAMs in tumors (mean ± s.e.m.; *n* = 10 mice). Statistical analysis by one-way ANOVA with Tukey’s multiple comparison test. **h**, Annotation of main cell populations identified by scRNA-seq of B16F10 tumors. The uniform manifold approximation and projection (UMAP) plot shows merged samples from both PBS and DCP-IL-12/FLT3L-treated mice. **i**, Expression of *Tyrp1* and *Cd68* shown on the UMAP. **j**, Most deregulated pathways in cancer cells (identified by *Tyrp1* expression) and myeloid cells (identified by *Cd68* expression) upon DCP-IL-12/FLT3L computed by overrepresentation analysis using Reactome pathways. Statistical analysis by one-tailed Fisher’s exact test followed by Benjamini–Hochberg *P* value correction. Pathways in blue are significant by adjusted *P* value. **k**, Left: representative images of real-size tumors immunostained for CD31 (green, endothelial cells) and stained with DAPI (blue), from mice treated as indicated. Scale bar, 250 μm. Right: quantification of the CD31^+^ area (mean ± s.e.m.; *n* = 6 mice for PBS and *n* = 8 for DCP-IL-12/FLT3L). Statistical analysis by two-tailed Mann–Whitney test. Each data point represents one sample from an independent mouse except for **b**, in which each data point represents the mean volume of independent tumors. Source data

**Extended Data Fig. 5:**
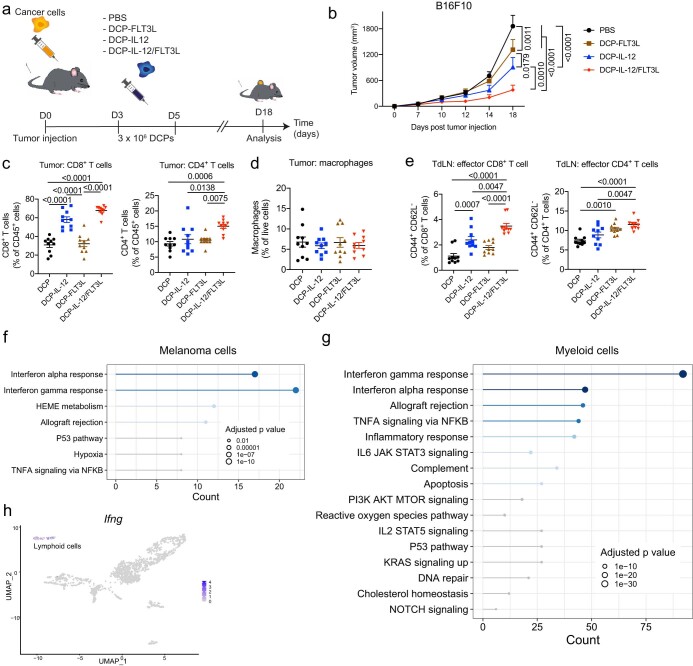
Cytokine-armed DCPs activate immunity and inhibit melanoma growth. **a**, Schematic of the experimental procedure to study adoptive transfer of cytokine-armed DCPs in B16F10 tumor-bearing mice (longer follow-up study). **b**, Tumor growth in mice with longer follow-up. Data show tumor volume (mean ± SEM; PBS, *n* = *5* mice; other groups, *n* = *6*). Statistical analysis by two-way ANOVA with Tukey’s multiple comparison test. Note that the PBS and DCP-IL-12/FLT3L group datasets are also shown in Fig. 4J, as the two studies were conducted in parallel. **c-e**, Frequency of the indicated cell types in B16F10 tumors and tdLNs (mean ± SEM; *n* = *10* mice) analyzed at day 15 from the experiment shown in Fig. 2A, B. Statistical analysis by one-way ANOVA with Tukey’s multiple comparison test. **f-g**, scRNA-Seq of B16F10 tumors. The overrepresentation analysis of the Hallmark database shows the most deregulated pathways in cancer cells (identified by *Tyrp1* expression) and myeloid cells (identified by *Cd68* expression) upon DCP-IL-12/FLT3L treatment, identified by unsupervised ranking according to Hallmark. Statistical analysis by one-tailed Fisher’s exact test followed by Benjamini-Hochberg p-value correction. Pathways in blue are significant by adjusted p value. **h**, *Ifng* expression in different cell clusters. Each data point represents one sample from an independent mouse, except for (b) in which each point represents the mean volume of independent tumors. Source data

We next performed DCP transfer studies in tumor-bearing mice by combining DCPs transduced with either IL-12 or FLT3L. In these experiments, IL-12 was coupled to the neutral marker dLNGFR (a truncated low-affinity human nerve growth factor receptor)^21^, whereas FLT3L was coupled to GFP. We administered a mixture of 1 × 10^6^ DCP-IL-12 and 2 × 10^6^ DCP-FLT3L to mice with B16F10 tumors on days three and five after tumor challenge (Fig. 2a). To examine the effects of DCPs expressing either IL-12 or FLT3L, we combined them with the appropriate number of DCPs expressing either GFP or dLNGFR, respectively. Control mice received phosphate-buffered saline (PBS) or DCPs expressing either GFP or dLNGFR. We found that a combination of DCP-IL-12 and DCP-FLT3L (hereafter DCP-IL-12/FLT3L) achieved superior tumor control compared to DCPs expressing either cytokine alone in independent experiments (Fig. 2b), including studies with longer follow-up analysis (Extended Data Fig. 5a,b). Serum levels of both IL-12 and FLT3L declined sharply from day one to day eight after the last DCP infusion (Fig. 2c), arguing against stable engraftment of cytokine-producing DCPs in recipient mice.

### Cytokine-armed DCPs activate antitumor immunity

B16F10 tumors—including the OVA-expressing variant employed in our study—contain scant T cell infiltrates and respond poorly to immune checkpoint blockade^22,23^. Flow cytometry analysis of intra-tumoral immune cells unveiled synergy between DCP-derived IL-12 and FLT3L. DCP-IL-12/FLT3L dramatically increased tumor infiltration by hematopoietic cells, CD8^+^ and CD4^+^ T cells compared to DCPs expressing either cytokine alone (Fig. 2d and Extended Data Fig. 5c). Moreover, ex vivo restimulation assays showed greater proportions of activated IFNγ^+^ T cells in some of the tumors of DCP-IL-12/FLT3L-treated mice (Fig. 2e). These results were corroborated by immunofluorescence staining of tumor sections and quantitative analysis (Fig. 2f). Both DCP-IL-12/FLT3L and DCP-IL-12 enhanced tumor-associated macrophage (TAM) expression of MHCII (>90%; Fig. 2g and Extended Data Fig. 5d), indicating acquisition of an immunostimulatory (M1-like) phenotype^24^. Finally, the relative abundance of CD44^+^CD62L^−^ T effector cells in tumor-draining lymph nodes (tdLNs) was higher in DCP-IL-12/FLT3L-treated mice (Extended Data Fig. 5e), suggesting activation of a systemic T cell response. These results indicate that IL-12/FLT3L-armed DCPs promote broad immune responses in a T cell-poor melanoma model.

### Cytokine-armed DCPs reprogram the tumor microenvironment through IFNγ

We then performed single-cell RNA sequencing (scRNA-seq) analysis of whole B16F10 tumors, which we analyzed 6 days after the second DCP administration. Owing to the substantial presence of melanosomes in melanoma preparations^25,26^, we could accurately identify only the major cell clusters (Fig. 2h). Nevertheless, we observed activation of IFNγ and type-I IFN signaling, as well as other immune-response pathways (for example, antigen presentation), in both cancer and myeloid cells of DCP-IL-12/FLT3L-treated tumors compared to vehicle-treated tumors, as shown by unsupervised ranking of the most deregulated biological processes according to Reactome and Hallmark (Fig. 2i,j and Extended Data Fig. 5f,g). As expected, IFNγ was only detectably expressed in the lymphoid-cell cluster (Extended Data Fig. 5h). Together with the flow-based data shown above, scRNA-seq analysis strongly suggests that DCP-IL-12/FLT3L instigated an IFNγ response that contributed to limiting tumor growth, at least partly, through effects on melanoma and myeloid cells. Additionally, there were antiangiogenic responses in DCP-IL-12/FLT3L-treated melanomas (Fig. 2k), which could have involved both direct vascular-pruning effects of IFNγ^27^ and indirect mechanisms; for example, through M1-programmed (angiostatic) TAMs^28^.

### DCPs provide a cell therapy platform alternative to moDCs

**Fig. 3:**
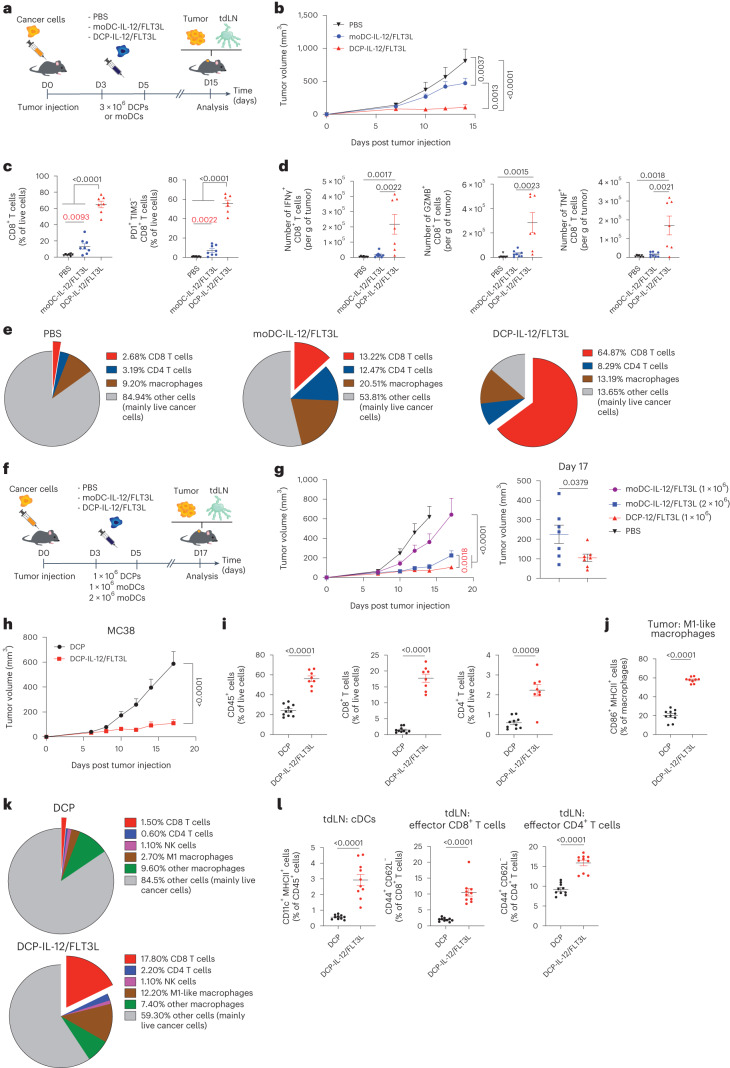
DCPs offer an effective cytokine-delivery platform alternative to moDCs. **a**, Procedure to study cytokine-armed DCPs and moDCs in B16F10 tumor-bearing mice. **b**, B16F10 tumor growth (mean ± s.e.m.; PBS, *n* = 7 mice; DCP-IL-12/FLT3L, *n* = 7; moDC-IL-12/FLT3L, *n* = 8). Statistical analysis by two-way ANOVA with Tukey’s multiple comparison test. **c**, Frequency of the indicated cell types in B16F10 tumors (mean ± s.e.m.; PBS, *n* = 7 mice; DCP-IL-12/FLT3L, *n* = 7; moDC-IL-12/FLT3L, *n* = 8). Statistical analysis by one-way ANOVA with Tukey’s multiple comparison test; *P* values in red were calculated by Mann–Whitney test on PBS versus moDC-IL-12/FLT3L. **d**, IFNγ, GZMB and TNF expression by ex vivo re-stimulated T cells (mean ± s.e.m.; PBS, *n* = 7 mice; DCP-IL-12/FLT3L, *n* = 7; moDC-IL-12/FLT3L, *n* = 8). Statistical analysis by one-way ANOVA with Tukey’s multiple comparison test. **e**, Pie charts showing the cell composition of B16F10 tumors (mean values; PBS, *n* = 7 mice; DCP-IL-12/FLT3L, *n* = 7; moDC-IL-12/FLT3L, *n* = 8). **f**, Procedure to study cytokine-armed DCPs and moDCs in B16F10 tumor-bearing mice. **g**, B16F10 tumor growth (mean ± s.e.m.; PBS, *n* = 8 mice; DCP-IL-12/FLT3L (1 × 10^6^), *n* = 7; moDC-IL-12/FLT3L (1 × 10^6^), *n* = 6; moDC-IL-12/FLT3L (2 × 10^6^), *n* = 7). Left, tumor volume over time; right, tumor volume at endpoint (day 17). Statistical analysis by two-way ANOVA with Tukey’s multiple comparison test; *P* value in red was calculated by two-way ANOVA with Sidak’s multiple comparison test on DCP-IL-12/FLT3L (1 × 10^6^) versus moDC-IL-12/FLT3L (2 × 10^6^). Statistical analysis at day 17 by two-tailed Mann–Whitney test. Note that the PBS and DCP-IL-12/FLT3L group datasets are also shown in Extended Data Fig. 7m, as the two studies were conducted in parallel. **h**, MC38 tumor growth (mean ± s.e.m.; *n* = 10 mice). Statistical analysis by two-way ANOVA with Sidak’s multiple comparison test. **i**,**j**, Frequency of the indicated cell types in MC38 tumors (mean ± s.e.m.; DCP, *n* = 10 mice; DCP-IL-12/FLT3L, *n* = 8 in **i** and *n* = 9 in). Statistical analysis by two-tailed Mann–Whitney test. **k**, Pie charts showing the cell composition of MC38 tumors (mean values; DCP, *n* = 10 mice; DCP-IL-12/FLT3L, *n* = 8). **l**, Frequency of the indicated cell types in MC38 tdLNs (mean ± s.e.m.; *n* = 10 mice). Statistical analysis by two-tailed Mann–Whitney test. Each data point represents one sample from an independent mouse except for **b**, **g** and **h**, in which each point represents the mean volume of independent tumors. Source data

**Extended Data Fig. 6:**
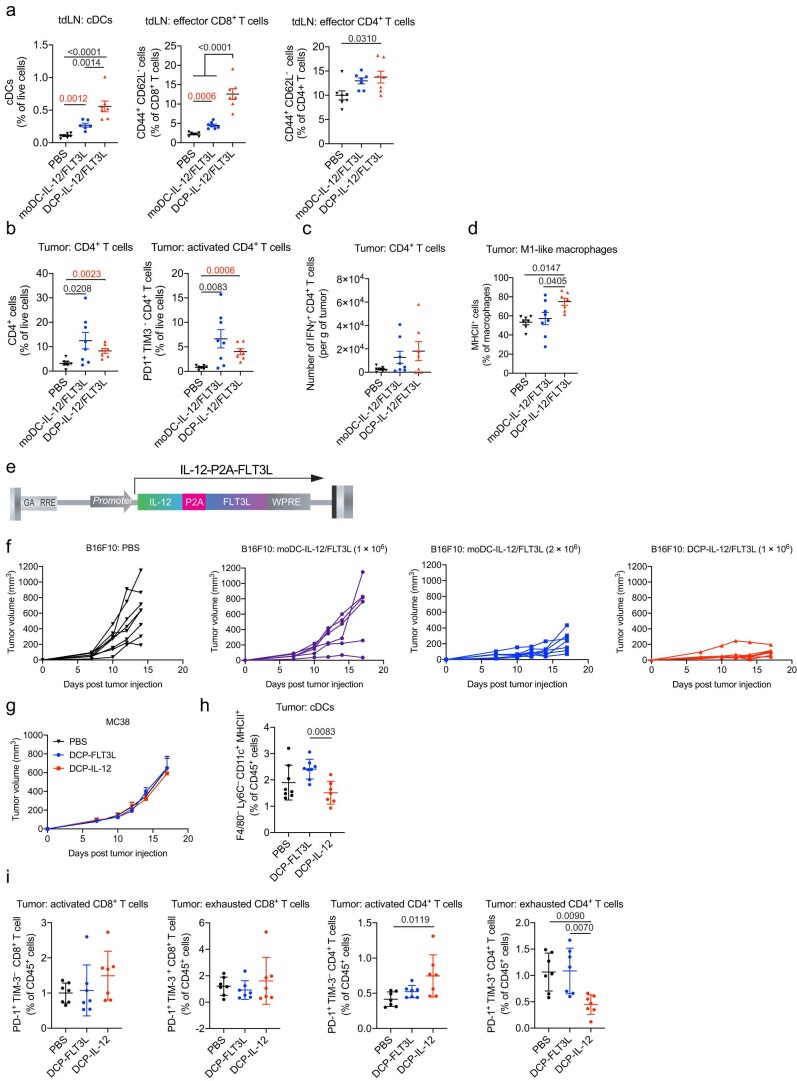
DCPs offer an effective cytokine-delivery platform alternative to moDCs. **a–d**, Frequency of the indicated cell types in B16F10 tdLNs and tumors of mice treated with cytokine-armed DCPs or moDCs (mean ± SEM; PBS, *n* = *7* mice; DCP-IL-12/FLT3L, *n* = *7*; moDC-IL-12/FLT3L, *n* = *7-8*). Statistical analysis by one-way ANOVA with Tukey’s multiple comparison test (black p values) or two-tailed Mann-Whitney test (red p values). **e**, Schematic of the bicistronic LV construct used to co-express IL-12 and FLT3L. **f**, Growth of individual B16F10 tumors in mice (PBS, *n* = *8* mice; DCP-IL-12/FLT3L (1 × 10^6^), *n* = *7*; moDC-IL-12/FLT3L (1 × 10^6^), *n* = *6*; moDC-IL-12/FLT3L (2 × 10^6^), *n* = *7*). **g**, MC38 tumor growth in mice. Data show tumor volume (mean ± SEM; *n* = *8* mice). Statistical analysis by two-way ANOVA with Tukey’s multiple comparison test (not significant). **h-i**, Frequency of the indicated cell types in MC38 tumors (mean ± SEM; PBS, *n* = *8* mice in (h) and *n* = *7* in (i); DCP-IL-12, *n* = *7*; DCP-FLT3L, *n* = *8* in (h) and *n* = *7* in (i)). Statistical analysis by one-way ANOVA with Tukey’s multiple comparison test. Each data point represents one sample or tumor measurement from an independent mouse, except for (g) in which each point represents the mean volume of independent tumors. Source data

Most preclinical and clinical experimentations with antigen-loaded DCs used moDCs^3^. We then investigated whether transgenic expression of IL-12 and FLT3L would confer upon moDCs the ability to expand T cells and control tumor growth in the absence of ex vivo antigen loading. We administered a mixture of 1 × 10^6^ DCP-IL-12 or moDC-IL-12, and 2 × 10^6^ DCP-FLT3L or moDC-FLT3L, to mice with B16F10 tumors on days three and five following tumor challenge (Fig. 3a). While DCP-IL-12/FLT3L achieved tumor stabilization, moDC-IL-12/FLT3L only delayed tumor growth (Fig. 3b). This outcome was associated with significantly higher CD8^+^ T cell infiltration and activation in tumors (Fig. 3c), as well as an expansion of cDCs and T effector (CD44^+^CD62L^−^) cells in tdLNs (Extended Data Fig. 6a), in mice that received DCP-IL-12/FLT3L. Moreover, tumors of DCP-IL-12/FLT3L-treated mice had higher proportions of CD8^+^ T cells expressing IFNγ, granzyme B (GZMB) and tumor necrosis factor (TNF) upon ex vivo restimulation (Fig. 3d). Although both DCP-IL-12/FLT3L and moDC-IL-12/FLT3L moderately increased CD4^+^ T cell infiltration and activation in tumors (Extended Data Fig. 6b,c), only DCP-IL-12/FLT3L enhanced expression of MHCII in TAMs (Extended Data Fig. 6d). Overall, the tumor cell composition in DCP-IL-12/FLT3L-treated mice was dominated by T cells, accounting for almost two-thirds of the live cells (Fig. 3e). This result may explain the pervasive IFNγ signature and marked M1 programming of TAMs observed after DCP-IL-12/FLT3L therapy.

The studies described above used a mix of DCs expressing either IL-12 or FLT3L in a 1:2 ratio. To explore the versatility of our platform, we co-expressed both cytokines from a single bicistronic LV (Extended Data Fig. 6e), that is, in the same cell, which represents a strategy better suited to clinical translation. DCP-IL-12/FLT3L and moDC-IL-12/FLT3L co-expressed IL-12 and FLT3L in vitro (Supplementary Fig. 7c) and exhibited antitumoral activity in the B16F10 model (Fig. 3f,g and Extended Data Fig. 6f). As observed in studies involving split LV transduction, DCPs were more effective than moDCs, although doubling the moDC-IL-12/FLT3L dose improved tumor control. Collectively, these preclinical results indicate that cytokine-armed DCPs provide an alternative strategy to moDCs for antigen-agnostic DC therapy applications.

### Cytokine-armed DCPs suppress MC38 tumor growth

We then tested DCP-IL-12/FLT3L in the MC38 model, which is characterized by abundant infiltrates of immunosuppressive TAMs^24,29^. MC38 tumor-bearing mice were treated as in the melanoma study shown in Fig. 3a above. DCP-IL-12/FLT3L achieved substantial MC38 tumor control (Fig. 3h and Extended Data Fig. 6g–i), facilitated immune-cell infiltration (Fig. 3i) and induced TAM acquisition of an M1-like phenotype (Fig. 3j,k). Additionally, DCP-IL-12/FLT3L strongly expanded cDCs and CD44^+^CD62L^−^ T effector cells in tdLNs (Fig. 3l), which aligns with findings in the B16F10 melanoma model.

### Tumor response to cytokine-armed DCPs is cDC1-dependent

**Fig. 4:**
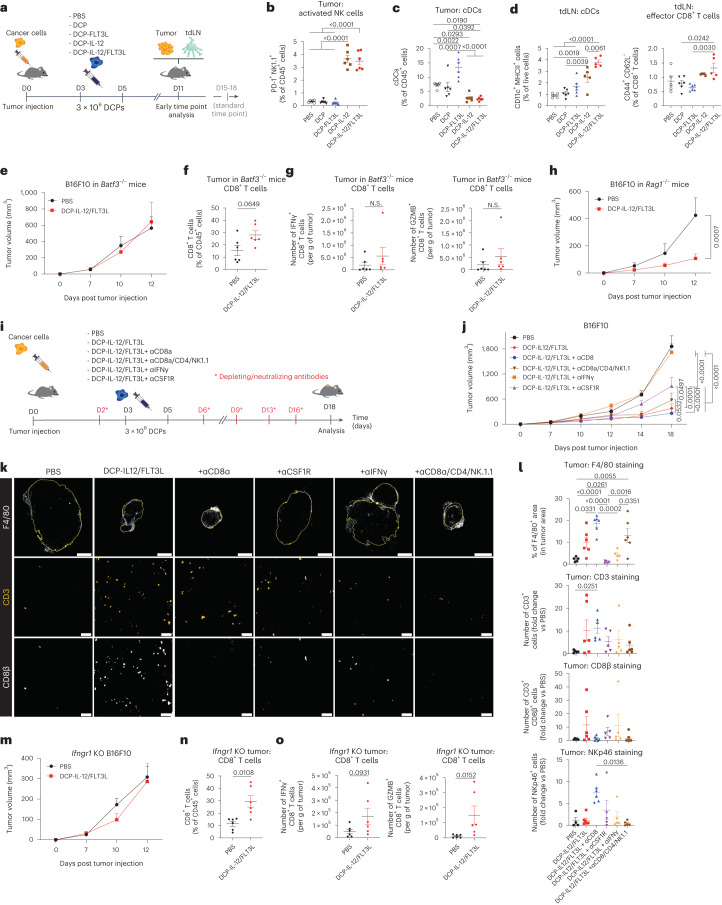
Tumor response to cytokine-armed DCPs is cDC1 and IFNγ-dependent but does not require CD8^+^ T cells. **a**, Procedure to study cytokine-armed DCPs in B16F10 tumor-bearing mice (early time point). **b**–**d**, Frequency of the indicated cell types in tumors (mean ± s.e.m.; *n* = 6 mice) and tdLNs (PBS, *n* = 5 mice; DCP, *n* = 6; DCP-FLT3L, *n* = 6; DCP-IL-12, *n* = 5; DCP-IL-12/FLT3L, *n* = 5). Statistical analysis by one-way ANOVA with Tukey’s multiple comparison test. **e**, B16F10 tumor growth in *Batf3*^–/–^ mice (mean volume ± s.e.m.; *n* = 6 mice). Statistical analysis by two-way ANOVA with Sidak’s multiple comparison test (not significant). **f**, Frequency of CD8^+^ T cells in tumors of *Batf3*^–/–^ mice (mean ± s.e.m.; *n* = 6 mice). Statistical analysis by two-tailed Mann–Whitney test. **g**, IFNγ and GZMB expression by ex vivo re-stimulated T cells (mean ± s.e.m.; *n* = 6 mice). Statistical analysis by two-tailed Mann–Whitney test (N.S., not significant). **h**, B16F10 tumor growth in *Rag1*^–/–^ mice (mean  ± s.e.m.; *n* = 6 mice). Statistical analysis by two-way ANOVA with Sidak’s multiple comparison test. **i**, Procedure to study cell or cytokine depletion. **j**, B16F10 tumor growth (mean ± s.e.m.; PBS, *n* = 5 mice; DCP-IL-12/FLT3L, DCP-IL-12/FLT3L + aCD8a and DCP-IL-12/FLT3L + aCD8a/CD4/NK1.1, *n* = 6; DCP-IL-12/FLT3L + aCSF1R and DCP-IL-12/FLT3L + aIFNγ, *n* = 5). Statistical analysis by two-way ANOVA with Tukey’s multiple comparison test. The PBS and DCP-IL-12/FLT3L datasets are also shown in Extended Data Fig. 5b (the two studies were conducted in parallel). **k**, Representative images of F4/80 (white), CD3 (orange) or CD8β (white) immunostaining of B16F10 tumors. Scale bar, 2 mm (top) or 50 μm (middle and bottom). **l**, Quantification of immune cells in tumors (mean ± s.e.m.; PBS, *n* = 5 mice, except for NKp46 staining where *n* = 4; DCP-IL-12/FLT3L, DCP-IL-12/FLT3L + aCD8a and DCP-IL-12/FLT3L + aCD8a/CD4/NK1.1, *n* = 6, except for NKp46 staining in DCP-IL-12/FLT3L + aCD8a/CD4/NK1.1 where *n* = 5; DCP-IL-12/FLT3L + aCSF1R and DCP-IL-12/FLT3L + aIFNγ, *n* = 5). Statistical analysis by one-way ANOVA with Tukey’s multiple comparison (F4/80) or Kruskal–Wallis test with Sidak’s multiple comparison (CD3, CD8β, NKp46). **m**, *Ifngr1*-knockout (KO) B16F10 tumor growth (mean ± s.e.m.; *n* = 6 mice). Statistical analysis by two-way ANOVA with Sidak’s multiple comparison test. **n**, Frequency of CD8^+^ T cells in *Ifngr1*-knockout B16F10 tumors (mean ± s.e.m.; *n* = 6 mice). Statistical analysis by two-tailed Mann–Whitney test. **o**, IFNγ and GZMB expression by ex vivo re-stimulated T cells from *Ifngr1*-knockout B16F10 tumors (mean ± s.e.m.; *n* = 6 mice). Statistical analysis by two-tailed Mann–Whitney test. Each data point represents one sample from an independent mouse except for **e**, **h**, **j** and **m**, in which each point represents the mean volume of independent tumors. Source data

**Extended Data Fig. 7:**
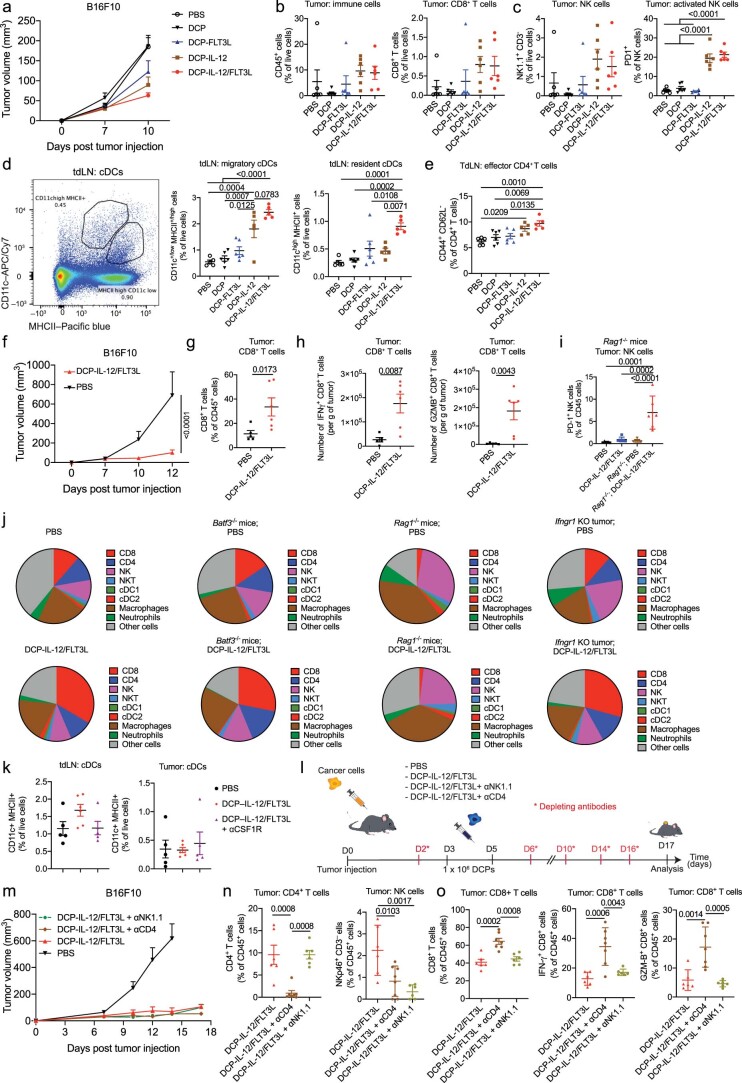
Tumor response to cytokine-armed DCPs is cDC1 and IFNγ-dependent but does not require CD8^+^ T cells. **a**, B16F10 tumor growth (early time point of analysis; mean volume ± SEM; *n* = 6 mice). **b-c**, Frequency of the indicated cell types in tumors at day 11 (mean ± SEM; *n* = 6 mice). Statistical analysis by one-way ANOVA with Tukey’s multiple comparison test**. d**, Left: flow cytometry analysis of migratory and resident cDCs in the tdLN of one representative mouse. Right: Quantification of migratory and resident cDCs (mean ± SEM; PBS, *n* = *5* mice; DCP, *n* = *6*; DCP-FLT3L, *n* = *6*; DCP-IL-12, *n* = 5; DCP-IL-12/FLT3L, *n* = *5*). Statistical analysis by one-way ANOVA with Tukey’s multiple comparison test. **e**, Frequency of CD4^+^ T effector cells in tdLN at day 11 (mean ± SEM; PBS, *n* = 5 mice; DCP, *n* = *6*; DCP-FLT3L, *n* = *6*; DCP-IL-12, *n* = *5*; DCP-IL-12/FLT3L, *n* = *5*). Statistical analysis by one-way ANOVA with Tukey’s multiple comparison test. **f**, B16F10 tumor growth (mean volume ± SEM; PBS, *n* = 5 mice; DCP-IL-12/FLT3L, *n* = *6*) in wild-type mice. Statistical analysis by two-way ANOVA with Sidak’s multiple comparison test. **g**, Frequency of CD8^+^ T cells in tumors (mean ± SEM; PBS, *n* = *5* mice; DCP-IL-12/FLT3L, *n* = *6*). Statistical analysis by two-tailed Mann-Whitney test. **h**, IFNγ and GZMB expression by ex vivo re-stimulated T cells (mean ± SEM; PBS, *n* = *5* mice; DCP-IL-12/FLT3L, *n* = *6*). Statistical analysis by two-tailed Mann-Whitney test. **i**, Frequency of activated NK cells in tumors of *Rag1*^–/–^ mice (mean ± SEM; *n* = *6* mice) and wild-type mice (PBS, *n* = 5 mice; DCP-IL-12/FLT3L, *n* = 6). Statistical analysis by one-way ANOVA with Tukey’s multiple comparison test. **j**, Pie charts showing the cell composition of B16F10 tumors (mean values; *n* = *6* mice for all groups, except for *n* = *5* in PBS). **k**, Frequency of cDCs in tdLN and tumors (mean ± SEM; PBS, *n* = *5* mice; DCP-IL-12/FLT3L, *n* = *6*; DCP-IL-12/FLT3L + anti-CSF1R, *n* = *5*). Statistical analysis by one-way ANOVA with Tukey’s multiple comparison test. **l**, Experimental procedure to study cell depletion in B16F10 tumor-bearing mice. **m**, B16F10 tumor growth (mean volume ± SEM; PBS, *n* = *8* mice; DCP-IL-12/FLT3L, *n* = *7*; DCP-IL-12/FLT3L + aCD4, *n* = *8*; DCP-IL-12/FLT3L + aNK1.1, *n* = *6*). Statistical analysis by two-way ANOVA with Tukey’s multiple comparison test. The PBS and DCP-IL12/FLT3L datasets are also shown in Fig. 3G, as the two studies were conducted in parallel. **n**, Frequency of the indicated cell types in tumors at day 17 (mean ± SEM; DCP-IL-12/FLT3L, *n* = *6* mice; DCP-IL-12/FLT3L + aCD4, *n* = *7* (left) or *8* (right); DCP-IL-12/FLT3L + aNK1.1, *n* = *6*). Statistical analysis by one-way ANOVA with Tukey’s multiple comparison test. **o**, IFNγ and GZMB expression by ex vivo re-stimulated CD8^+^ T cells isolated from tumors (mean ± SEM; DCP-IL-12/FLT3L, *n* = *6* mice; DCP-IL-12/FLT3L + aCD4, *n* = *7*; DCP-IL-12/FLT3L + aNK1.1, *n* = *6*). Statistical analysis by one-way ANOVA with Tukey’s multiple comparison test. Each data point represents one sample from an independent mouse, except for (a), (f), and (m) in which each point represents the mean volume of independent tumors. Source data

To explore potential mechanisms of tumor response to DCP-IL-12/FLT3L, we studied B16F10 tumors at an early time point after DCP transfer (6 days after the second DCP dose; Fig. 4a). At this early stage of tumor development, there were scarce hematopoietic and CD8^+^ T cell infiltrates, which were only moderately increased by DCP-IL-12 or DCP-IL-12/FLT3L (Extended Data Fig. 7a,b). However, the latter treatments markedly increased the abundance of activated natural killer (NK) cells in the tumors (Fig. 4b and Extended Data Fig. 7C). Thus, early tumor responses to DCP-IL-12/FLT3L may primarily involve IL-12-dependent effects on NK cells. Interestingly, DCP-IL-12 and DCP-IL-12/FLT3L exhibited a dual effect, reducing cDCs in tumors while simultaneously augmenting cDCs—including CD11c^low/+^MHCII^+/high^ migratory cDCs—in tdLNs (Fig. 4c,d and Extended Data Fig. 7d,e). This response was accompanied by a moderate increase in CD44^+^CD62L^−^ T effector cells in tdLNs. Conversely, DCP-FLT3L induced a more pronounced increase in cDCs within tumors compared to tdLNs. These results support the hypothesis that DCP-derived FLT3L directly promotes the initial expansion of endogenous cDCs in tumors, while IL-12 instigates their migration from the tumor microenvironment (TME) to the tdLN through NK cell-derived IFNγ. This migration would enable the cDCs to initiate T cell priming in the tdLN.

We then asked whether endogenous cDC1 and T cells are required for tumor inhibition in response to DCP-IL-12/FLT3L. Absence of endogenous cDC1 in *Batf3*^−/−^ recipient mice fully negated the therapeutic activity of DCP-IL-12/FLT3L (Fig. 4e and Extended Data Fig. 7f–h) and failed to elicit robust T cell infiltration and activation (Fig. 4f,g) in B16F10 tumors, suggesting that cytokine-armed DCPs engage endogenous cDC1 to activate antitumor immunity. Surprisingly, DCP-IL-12/FLT3L were also effective in *Rag1*^−/−^ mice (Fig. 4h), which lack mature T cells and B cells^30^. In B16F10 tumors of *Rag1*^−/−^ mice, we observed greatly heightened proportions of activated, PD-1^+^ NK cells (Extended Data Fig. 7i), which may explain, at least in part, the persistence of antitumoral effects. An overview of the immune cell composition of B16F10 tumors inoculated in *Batf3*^−/−^, *Rag1*^−/−^ and wild-type mice is shown in Extended Data Fig. 7j. These results led us to hypothesize that the antitumoral activity of DCP-IL-12/FLT3L relied on endogenous cDC1 but did not require the tumoricidal functions of T effector cells.

### Tumor response is IFNγ-dependent and CD8^+^ T cell-independent

To gain further insight into the involvement of T cells in tumor response to DCP-IL-12/FLT3L, we conducted cell depletion and cytokine neutralization studies in mice with B16F10 tumors (Fig. 4i). In line with findings in *Rag1*^−/−^ mice, elimination of CD8^+^ T cells did not affect tumor response to DCP-IL-12/FLT3L and, remarkably, simultaneous elimination of CD8^+^ T cells, CD4^+^ T cells and NK1.1^+^ NK cells only moderately rescued tumor growth (Fig. 4j). By contrast, neutralization of IFNγ fully rescued tumor growth, while depletion of TAMs using a colony-stimulating factor 1 receptor (CSF1R) antibody partly rescued tumor growth. Of note, CSF1R did not affect cDC numbers in tumors and tdLNs (Extended Data Fig. 7k), consistent with studies in *Csf1r*^−/−^ mice^31^. Given that flow cytometry analysis captures relative cell proportions, we used immunofluorescence staining of tumor sections with noncompeting antibodies to obtain quantitative data (Fig. 4k,l). DCP-IL-12/FLT3L increased F4/80^+^ TAMs, a response that was abrogated by both CSF1R blockade and IFNγ neutralization but not by T cell and NK cell elimination. Furthermore, DCP-IL-12/FLT3L augmented both CD8^+^ and total CD3^+^ T cells, but eliminating CD8^+^ T cells did not decrease overall CD3^+^ T cell numbers, suggesting compensatory tumor infiltration by CD3^+^CD8^−^ T cells. Interestingly, CD8^+^ T cell elimination increased NK cells and TAMs, which remained elevated even after the combined elimination of CD8^+^ and CD4^+^ T cells and NK cells. In an independent study, eliminating CD4^+^ T cells or NK1.1^+^ NK cells individually did not impair tumor response to DCP-IL-12/FLT3L treatment (Extended Data Fig. 7l,m), and disrupting CD4^+^ T cells was associated with compensatory increases in both total and activated (IFNγ^+^ or GZMB^+^) CD8^+^ T cells (Extended Data Fig. 7n,o). Collectively, these findings indicate that B16F10 tumor response to DCP-IL-12/FLT3L is strictly IFNγ-dependent, involves a diverse assortment of IFNγ-producing cells and may depend, at least in part, on the antitumoral activity of IFNγ-stimulated M1-like TAMs.

**Extended Data Fig. 8:**
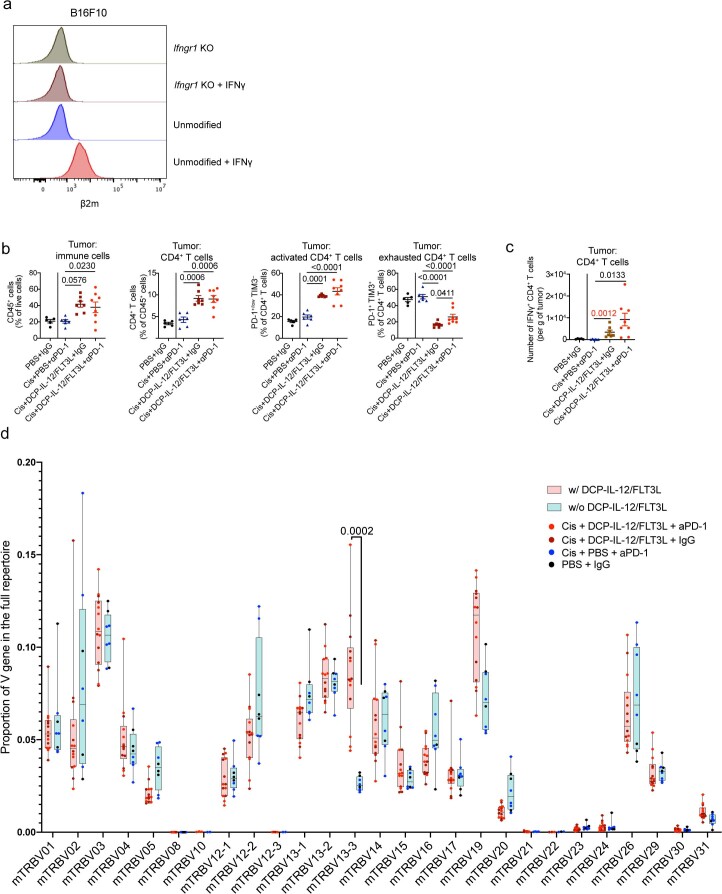
Cytokine-armed DCPs improve the efficacy of cisplatin/anti-PD-1 in a colorectal cancer model. **a**, Flow cytometry analysis of B2M (indicative of MHCI expression) in the indicated B16F10 cells, treated as indicated. Note that *Ifngr1* KO B16F10 fail to upregulate B2M in response to IFNγ. **b**, Frequency of the indicated cell types in MC38 tumors (mean ± SEM; PBS + IgG, *n* = *5* mice; cis + PBS + ɑPD-1, *n* = *6*; cis + DCP-IL-12/FLT3L + IgG, *n* = *7*; cis + DCP-IL-12/FLT3L + ɑPD-1, *n* = *8*). Statistical analysis by one-way ANOVA with Tukey’s multiple comparison test (the analysis excludes tumors in the PBS + IgG, which were processed independently). **c**, IFNγ expression by ex vivo re-stimulated T cells from the tumors treated as indicated (mean ± SEM; PBS + IgG, *n* = *5* mice; cis + PBS + ɑPD-1, *n* = *6*; cis + DCP-IL-12/FLT3L + IgG, *n* = *7*; cis + DCP-IL-12/FLT3L + ɑPD-1, *n* = *8*). Statistical analysis by one-way ANOVA with Tukey’s multiple comparison test (black p values) or two-tailed Mann-Whitney test (red p values). **d**, V gene usage profile. Data were extracted from bulk TCR-Seq of tumor samples (PBS + IgG, *n* = *3* mice; cis + PBS + ɑPD-1, *n* = *5*; cis + DCP-IL-12/FLT3L + IgG, *n* = *7*; cis + DCP-IL-12/FLT3L + ɑPD-1, *n* = *7*). Box plots show median (central bar), numerical data through their 3rd and 1st quartiles (box), and maximum and minimun values (whiskers). All tumors from mice that received DCP-IL-12/FLT3L were combined and compared with tumors from mice that did not receive DCP-IL-12/FLT3L. Statistical analysis by multiple Wilcoxon rank sum test corrected with the Holm-Sidak method. Each data point represents one sample from an independent mouse. Source data

Finally, to explore potential direct effects of IFNγ on B16F10 melanoma cells, we generated *Ifngr1*-knockout B16F10 cells. Abrogation of cancer-cell responsiveness to IFNγ (Extended Data Fig. 8a) was sufficient to negate the therapeutic activity of DCP-IL-12/FLT3L in mice (Fig. 4m), despite the observed enhancement of intratumoral CD8^+^ T cell infiltration and activation (Fig. 4n,o). Taken together, our results position IFNγ as a key mediator of tumor inhibition and strongly suggest that IFNγ production, rather than direct CD8^+^ T cell cytotoxicity, is required for therapeutic response to DCP-IL-12/FLT3L.

### Cytokine-armed DCPs improve efficacy of chemoimmunotherapy

**Fig. 5:**
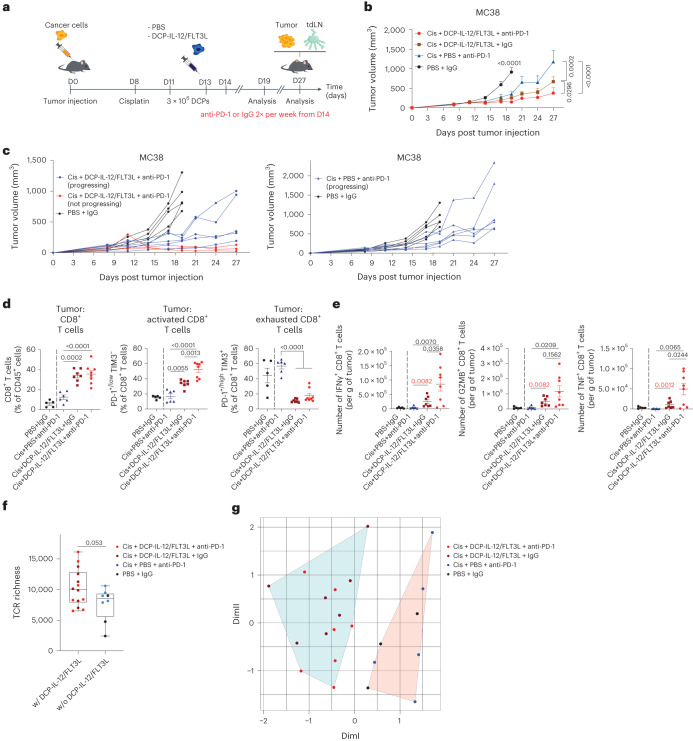
Cytokine-armed DCPs improve the efficacy of cisplatin and PD-1 blockade in a colorectal cancer model. **a**, Procedure to study MC38 tumor response to cytokine-armed DCPs in combination with cisplatin (cis) and anti-PD-1. **b**,**c**, MC38 tumor growth showing mean tumor growth (**b**) and growth of individual tumors (**c**). Data show tumor volume (mean ± s.e.m.; PBS + IgG, *n* = 5 mice; cis + PBS + anti-PD-1, *n* = 6; cis + DCP-IL-12/FLT3L + IgG, *n* = 7; cis + DCP-IL-12/FLT3L + anti-PD-1, *n* = 8). Statistical analysis by two-way ANOVA with Tukey’s multiple comparison test. **d**, Frequency of the indicated cell types in MC38 tumors (mean ± s.e.m.; PBS + IgG, *n* = *5* mice; cis + PBS + anti-PD-1, *n* = 6; cis + DCP-IL-12/FLT3L + IgG, *n* = 7; cis + DCP-IL-12/FLT3L + anti-PD-1, *n* = 8). Statistical analysis by one-way ANOVA with Tukey’s multiple comparison test (the analysis excludes tumors in the PBS + IgG group, which were processed independently). **e**, IFNγ, GZMB and TNF expression by ex vivo re-stimulated T cells (mean ± s.e.m.; PBS + IgG, *n* = 5 mice; cis + PBS + anti-PD-1, *n* = 6; cis + DCP-IL-12/FLT3L + IgG, *n* = 7; cis + DCP-IL-12/FLT3L + anti-PD-1, *n* = 8). Statistical analysis by one-way ANOVA with Tukey’s multiple comparison test (black *P* values) or two-tailed Mann–Whitney test (red *P* values, comparing two groups of interest). **f**, Diversity of TCR repertoire (PBS + IgG, *n* = 3 mice; cis + PBS + anti-PD-1, *n* = 5; cis + DCP-IL-12/FLT3L + IgG, *n* = 7; cis + DCP-IL-12/FLT3L + anti-PD-1, *n* = 7) assessed in bulk TCR sequencing (TCR-seq) on mRNAs isolated from fresh–frozen MC38 tumor samples. TCR diversity was estimated by TCR richness. Box plots show median (central bar), numerical data through their third and first quartiles (box), and maximum and minimum values (whiskers). All tumors from mice that received DCP-IL-12/FLT3L were combined and compared with tumors from mice that did not receive DCP-IL-12/FLT3L. Statistical analysis by two-tailed Student’s *t-*test after normality testing by Shapiro–Wilk test and *Q*–*Q* plot visualization. **g**, *K*-means clustering of bulk TCR-seq data based on V gene usage, showing separation of samples containing DCP-IL-12/FLT3L (*n* values as in **f**). Each data point represents one sample or tumor measurement from an independent mouse except for **b**, in which each data point represents the mean volume of independent tumors. Source data

B16F10 and MC38 tumors exhibit rapid growth kinetics, which complicates the assessment of therapeutic interventions in advanced tumors. To slow MC38 tumor growth, we pretreated MC38 tumor-bearing mice with a single dose of cisplatin, a chemotherapeutic drug used in colorectal cancer treatment^32^. This was followed by DCP-IL-12/FLT3L on days 11 and 13, and PD-1 blocking antibodies starting on day 14 twice weekly (Fig. 5a). Although a combination of cisplatin and anti-PD-1 delayed tumor growth, the addition of DCP-IL-12/FLT3L to cisplatin improved the antitumor response, which was further ameliorated by PD-1 blockade (Fig. 5b). The combined treatment (cisplatin, DCPs, anti-PD-1) resulted in tumor regression or stabilization in three out of eight mice (day 27 versus day 11), whereas all tumors progressed in the other groups (Fig. 5c). DCP-IL-12/FLT3L combined with cisplatin increased intratumoral infiltration by hematopoietic cells, CD8^+^ and CD4^+^ T cells, independent of PD-1 blockade (Fig. 5d and Extended Data Fig. 8b). Notably, the majority of T cells exhibited a non-exhausted, activated phenotype. Additionally, ex vivo restimulation assays showed enhanced expression of IFNγ, GZMB and TNF in CD8^+^ T cells, and elevated IFNγ in CD4^+^ T cells (Fig. 5e and Extended Data Fig. 8c) in tumors of mice that received the full treatment regimen, which may explain the additive benefits of PD-1 blockade. Conversely, mice treated with cisplatin and anti-PD-1 had non-elevated intratumoral CD8^+^ or CD4^+^ T cells, which mostly exhibited an exhausted phenotype. These data indicate that cytokine-armed DCPs improve chemoimmunotherapy efficacy in a colorectal cancer model.

### Cytokine-armed DCPs increase T cell receptor diversity in a tumor model

We examined T cell diversity in MC38 tumors by bulk sequencing of T cell receptor beta (TCRβ). We observed a trend towards higher diversity in the T cell repertoire of tumors that received DCP-IL-12/FLT3L, as shown by the higher number of unique clonotypes (Fig. 5f). Interestingly, T cells in tumors of mice that received DCP-IL-12/FLT3L shared significant similarity in terms of V gene usage in their TCR, as indicated by unsupervised *K*-means clustering (Fig. 5g and Extended Data Fig. 8d). These findings suggest that DCP-IL-12/FLT3L promotes expansion of T cells with shared specificity towards MC38 tumor-associated antigens.

### Cytokine-armed DCPs are effective in liver cancer models

**Fig. 6:**
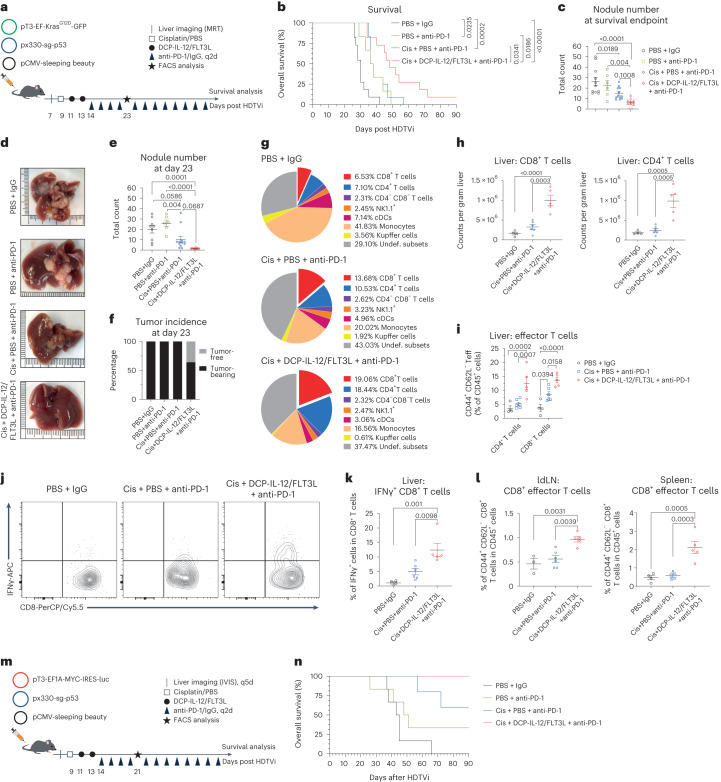
Cytokine-armed DCPs are effective in two genetically engineered liver cancer models. **a**, Induction and treatment of *Kras*^G12D^; *Trp53*^–/–^ liver tumors. Mice were monitored for up to 90 days. **b**, Survival of *Kras*^G12D^; *Trp53*^–/–^ tumor-bearing mice (PBS + IgG, *n* = 11 mice, 30 days; PBS + anti-PD-1, *n* = 6, 37 days; cis + PBS + anti-PD-1, *n* = 12, 37 days; cis + DCP-IL-12/FLT3L + anti-PD-1, *n* = 11, 48 days). Statistical analysis by log-rank Mantel–Cox test. Two independent experiments combined (one mouse was terminated while being tumor-free). **c**, Number of *Kras*^G12D^; *Trp53*^–/–^ liver nodules (mean ± s.e.m.) at survival endpoint (*n* values as in **b**). Statistical analysis by one-way ANOVA with Turkey’s multiple comparisons test. **d**, Representative *Kras*^G12D^; *Trp53*^–/–^ livers analyzed at day 23. Two independent experiments combined. **e**, Number of *Kras*^G12D^; *Trp53*^–/–^ liver nodules at day 23 (mean ± s.e.m.; PBS + IgG, *n* = 9 mice; PBS + anti-PD-1, *n* = 6; cis + PBS + anti-PD-1, *n* = 12; cis + DCP-IL-12/FLT3L + anti-PD-1, *n* = 11). Two independent experiments combined. Statistical analysis by one-way ANOVA with Turkey’s multiple comparisons test. **f**, *Kras*^G12D^; *Trp53*^–/–^ tumor incidence at day 23 (*n* values as in **d**). Two independent experiments combined**. g**, Pie charts showing immune cell composition of *Kras*^G12D^; *Trp53*^–/–^ livers at day 23 from one of two experiments (mean values; PBS + IgG, *n* = 4 mice; cis + PBS + anti-PD-1, *n* = 6; cis + DCP-IL-12/FLT3L + anti-PD-1, *n* = 5). **h**,**i**, Frequency of the indicated cell types in *Kras*^G12D^; *Trp53*^–/–^ livers at day 23 (mean ± s.e.m.; *n* values as in **g**). Statistical analysis by one-way ANOVA with Turkey’s multiple comparisons test (**h**) or two-way ANOVA with Turkey’s multiple comparisons test (**i**). **j**,**k**, IFNγ expression by ex vivo re-stimulated T cells from *Kras*^G12D^; *Trp53*^–/–^ livers at day 23 (mean ± s.e.m.; *n* values as in **g**). Statistical analysis one-way ANOVA with Turkey’s multiple comparisons test. Representative CD8^+^ T cells are shown in **j**. **l**, Frequency of the indicated cell types in *Kras*^G12D^; *Trp53*^–/–^ liver-draining lymph nodes (ldLNs) and spleens at day 23 (mean ± s.e.m.; *n* values as in **g**, except for PBS + IgG in ldLNs where *n* = 3 mice). Statistical analysis one-way ANOVA with Turkey’s multiple comparisons test. **m**, Induction and treatment of *Myc*; *Trp53*^–/–^ liver tumors. Mice were monitored for up to 90 days**. n**, Survival of *Myc*; *Trp53*^–/–^ tumor-bearing mice (PBS + IgG, *n* = 6 mice, 44 days; PBS + anti-PD-1, *n* = 6, 49.5 days; cis + PBS + anti-PD-1, *n* = 5, undefined; cis + DCP-IL-12/FLT3L + anti-PD-1, *n* = 6, undefined). Statistical analysis not applicable. Each data point represents one sample from an independent mouse. Source data

We asked whether DCPs are also effective in two genetically engineered liver cancer models obtained by hydrodynamic tail vein injection (HDTVi) of cancer-causing plasmids. In the first model, activation of a *Kras*^G12D^ oncogene and deletion of *Trp53* in liver hepatocytes induces multifocal liver tumors with features of hepatocellular carcinoma and cholangiocarcinoma. *Kras*^*G12D*^; *Trp53*^−/−^ tumors establish an immunosuppressive and nearly immune-desert TME, and exhibit aggressive growth patterns with median mouse survival of approximately 30 days^33^. Tumor-initiated mice were treated with anti-PD-1 alone, a combination of cisplatin and anti-PD-1 or DCP-IL-12/FLT3L with cisplatin and anti-PD-1 (Fig. 6a). DCP-IL-12/FLT3L extended survival significantly compared to other treatments (Fig. 6b). Notably, these mice had fewer large tumors, despite a longer time that had elapsed, on average, between tumor induction and termination (Fig. 6c).

**Extended Data Fig. 9:**
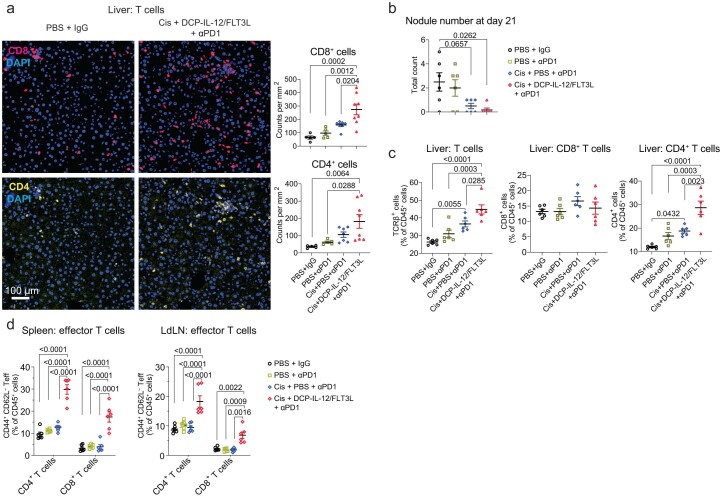
Cytokine-armed DCPs are effective in two genetically engineered liver cancer models. **a**, Left: representative images of CD8 (red) or CD4 (yellow) immunostaining, and DAPI nuclear staining (blue), of livers of Kras^G12D^/Tp53^–/–^ tumor-bearing mice treated as indicated and analyzed at day 23. Scale bar, 100 μm. Right: quantification of the data (mean ± SEM; PBS + IgG, *n* = *5* mice; PBS + aPD1, *n* = *5*; Cis + PBS + aPD1, *n* = *7*; Cis + DCP–IL-12/FLT3L + aPD1, *n* = *8*). Statistical analysis by one-way ANOVA with Tukey’s multiple comparisons test. **b**, Number of liver nodules in cMyc/Tp53^–/–^ tumor-bearing mice treated as indicated and analyzed at day 21 (mean ± SEM; *n* = *6* mice). Statistical analysis one-way ANOVA with Tukey’s multiple comparisons test. **c, d**, Frequency of the indicated cell types in livers or liver-draining lymph nodes (ldLNs) of in cMyc/Tp53^–/–^ tumor-bearing mice treated as indicated and analyzed at day 21 (mean ± SEM; *n* = *6* mice). Statistical analysis one-way ANOVA with Turkey’s multiple comparisons test (c) and two-way ANOVA with Sidak’s multiple comparisons test (d). Each data point represents one sample from an independent mouse. Source data

In two independent studies, we analyzed the livers at a fixed time point (day 23 after tumor initiation). At this time point, the mice in the triple combination cohort had substantially reduced numbers of macroscopic liver tumors and four out of eleven mice were tumor-free (Fig. 6d–f), consistent with results of the survival study. Immunofluorescence staining of the liver parenchyma revealed increased density of CD8^+^ and CD4^+^ T cells in the majority of the mice that received DCP-IL-12/FLT3L compared to other treatments (Extended Data Fig. 9a). We also examined immune cell parameters by flow cytometry at the same time point of analysis (day 23). DCP-IL-12/FLT3L expanded CD8^+^ and CD4^+^ T cells in the liver parenchyma, both as proportions of CD45^+^ hemopoietic cells (Fig. 6g) and absolute cell counts (Fig. 6h). Moreover, DCP-IL-12/FLT3L increased CD44^+^CD62L^−^ CD8^+^ and CD4^+^ T effector cells (Fig. 6i) and IFNγ^+^ CD8^+^ T cells (Fig. 6j,k). Higher proportions of CD44^+^CD62L^−^ CD8^+^ T effector cells were also observed in liver-draining lymph nodes and spleen (Fig. 6l).

We then employed an HDTVi-based *Myc*-driven and *Trp53*-deleted liver cancer model, which develops fewer tumors than the *Kras*^G12D^; *Trp53*^−/−^ model. *Myc*; *Trp53*^−/−^ tumors have features of hepatocellular carcinoma, harbor dysfunctional DCs and are resistant to immune checkpoint blockade^34^. Tumor-initiated mice were treated with anti-PD-1 alone, a combination of cisplatin and anti-PD-1 or DCP-IL-12/FLT3L with cisplatin and anti-PD-1 (Fig. 6m). In this model, DCP-IL-12/FLT3L achieved 100% survival, which was superior to survival rates in the other treatment groups (Fig. 6n). Furthermore, an independent mouse cohort analyzed at a fixed time point (day 21 after tumor initiation) showed that five out of six mice in the DCP-IL-12/FLT3L group had no evidence of macroscopic tumors, whereas at least 50% of the mice had tumors in the other groups (Extended Data Fig. 9b). Flow cytometry analysis of the liver parenchyma showed increased proportions of CD4^+^ (but not CD8^+^) T cells in mice that received DCP-IL-12/FLT3L (Extended Data Fig. 9c); this response was associated with elevated proportions of CD4^+^ and CD8^+^ T effector cells in both liver-draining lymph nodes and spleen (Extended Data Fig. 9d). In summary, cytokine-armed DCPs improve tumor response to cisplatin and anti-PD-1 by eliciting IFNγ-producing T cells, decreasing tumor multiplicity and extending survival in two aggressive liver cancer models.

### Cytokine-armed DCPs synergize with CAR-T in a glioma model

**Extended Data Fig. 10:**
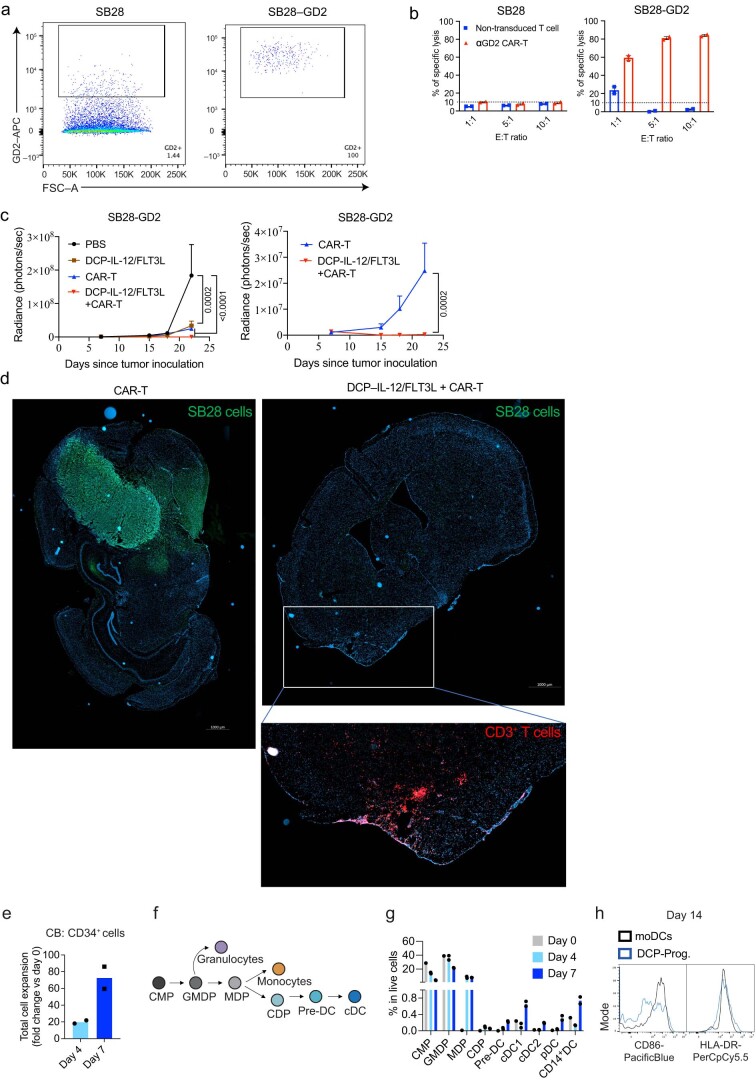
Cytokine-armed DCPs synergize with CAR-T cells to eradicate mouse gliomas and can be generated from human HSPCs. **a**, Expression of GD2 by S28-GD2 cancer cells. **b**, Antigen-specific cytotoxic activity by CAR-T cells. Engineered anti-GD2 CAR-T cells were cocultured with unmodified SB28 or SB-28-GD2^+^ cells at the indicated cell ratios. Unmodified T cells were used as control. Data show 3 technical replicates per condition. **c**, Tumor burden assessed by IVIS imaging (mean radiance ± SEM; PBS, *n* = *7* mice; DCP-IL-12/FLT3L, *n* = *6*; CAR-T, *n* = *8*; DCP-IL-12/FLT3L + CAR-T, *n* = *8*) shown until day 22, a time point when all but one mouse were still alive (note that one mouse in the CAR-T group was terminated on day 18, so the data on day 22 show 7 of 8 tumors in the CAR-T group). Statistical analysis by two-way ANOVA with Tukey’s (left) or Sidak’s (right) multiple comparison test**. d**, representative images of GFP (green, marking SB28 glioma cells) or CD3 (red) immunostaining, and DAPI nuclear staining (blue), of brain sections from two representative mice treated as indicated. Scale bar: 1 mm. **e**, Number of cells after CB CD34^+^ cell expansion, analyzed at day 4 or 7 (fold-change relative to day 0). The data show one representative donor of two, and two technical replicates (data points). **f**, Schematic view of human DC hematopoiesis. **g**, Percentage of the indicated cell types in total live CB-derived cells analyzed at day 0, 4 and 7. The data show one representative donor and two technical replicates (data points). **h**, Representative flow cytometry histograms of CD86 and HLA-DR expression in moDCs and MPB-derived DCP progeny (DCP-Prog.) assessed on day 14. Each data point represents one sample from an independent mouse, except for (b), (g), and (h) in which each point represents the mean volume of independent tumors. Common myeloid progenitor (CMP); Granulocyte, monocyte and DC progenitor (GMDP); Monocyte and DC progenitor (MDP); Common-dendritic cell progenitor (CDP); cDC precursor (Pre-DC); Conventional DC (cDC). Source data

CAR-T are T cells with engineered tumor specificity. While CAR-T can recognize and kill cancer cells that express the targeted antigen, their therapeutic efficacy in solid tumors is limited by antigenic heterogeneity and the immunosuppressive TME^35^. We used the aggressive SB28 murine glioma model, which recapitulates key features of human glioblastoma, is immunologically silent and is not responsive to immune checkpoint blockade^36^. We used CAR-T specific to GD2, a disialogangloside expressed in subsets of human gliomas and a clinically validated CAR-T target^37,38^. GD2^+^ SB28 glioma cells were generated by transducing the parental cell line with LVs encoding the GD2 synthases, GD2S and GD3S (Extended Data Fig. 10a), as previously described^7^. Anti-GD2 CAR-T efficiently killed GD2^+^ but not GD2^−^ SB28 cells in vitro (Extended Data Fig. 10b).

**Fig. 7:**
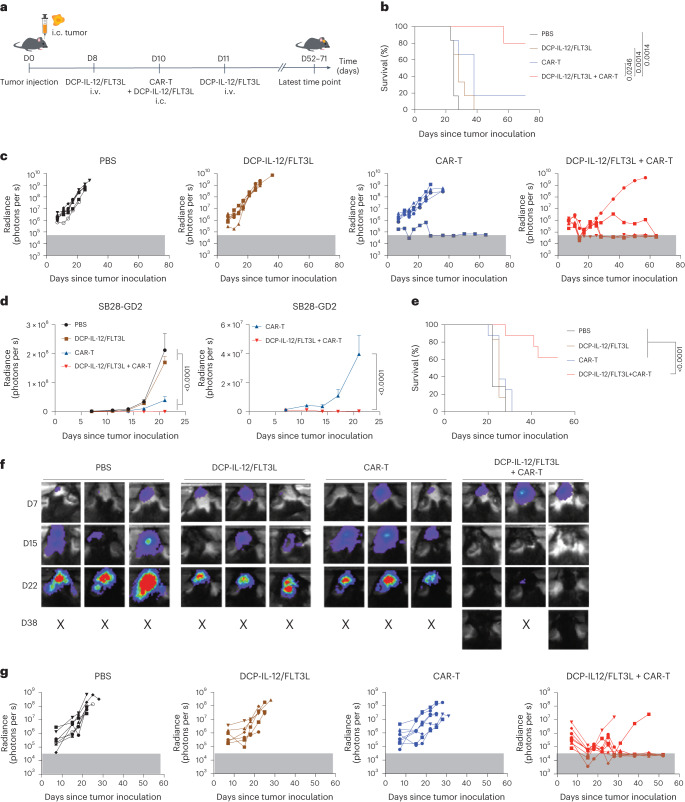
Cytokine-armed DCPs synergize with CAR-T cells to eradicate mouse gliomas. **a**, Procedure to study the combination of DCP-IL-12/FLT3L and CAR-T cells for the treatment of the SB28-GD2 glioma model. Mice received cytokine-armed DCPs both intracranially (i.c.) and intravenously (i.v.), and CAR-T cells intracranially. **b**, Survival analysis (PBS, *n* = 6 mice; DCP-IL-12/FLT3L, *n* = 6; CAR-T, *n* = 6; DCP-IL-12/FLT3L + CAR-T, *n* = 5). Mice were monitored for up to 71 days. Statistical analysis by log-rank Mantel–Cox test. **c**, Growth of individual tumors assessed by in vivo fluorescence imaging (IVIS) (*n* values as in **b**). The gray box indicates background radiance signal. **d**, Tumor burden quantified by IVIS imaging (mean radiance ± s.e.m.; *n* values as in **b**) shown until day 20, a time point when all the mice were still alive. The graph on the right shows the CAR-T and DCP-IL-12/FLT3L + CAR-T treatment groups separately. Statistical analysis by two-way ANOVA with Tukey’s (left) or Sidak’s (right) multiple comparison test**. e**, Survival analysis (PBS, *n* = 7 mice; DCP-IL-12/FLT3L, *n* = 6; CAR-T, *n* = 8; DCP-IL-12/FLT3L + CAR-T, *n* = 8). One mouse in the DCP-IL-12/FLT3L + CAR-T cohort was terminated while being tumor-free according to IVIS and post-mortem analysis. Mice were monitored for up to 52 days. Statistical analysis by log-rank Mantel–Cox test. **f**, IVIS imaging data of three representative mice from **e** per treatment condition (*n* values as in **e**). **g**, Growth of individual tumors assessed by IVIS imaging (*n* values as in **e**). The gray box indicates background radiance signal. Each data point in **c** and **g** represents one tumor measurement; each data point in **d** represents the mean volume of independent tumors. Source data

We inoculated mice with SB28-GD2 cells intracranially and treated them with DCP-IL-12/FLT3L, anti-GD2 CAR-T or a combination of both (Fig. 7a). Tumor progression was monitored by longitudinal live imaging analysis. Mice receiving a combination of DCP-IL-12/FLT3L and CAR-T survived significantly longer than those receiving either cell therapy alone (Fig. 7b). While DCP-IL-12/FLT3L or CAR-T monotherapies moderately delayed tumor progression, all but one mouse in the CAR-T cohort developed progressive disease (Fig. 7c,d). Conversely, the combined treatment induced tumor regression in four out of five mice, which remained tumor-free until the study termination (day 71 after tumor inoculation).

The study described above used a mixture of DCPs expressing either IL-12 or FLT3L. We then repeated the glioma study by using DCPs singly transduced with the bicistronic IL-12/FLT3L LV. Consistent with the results shown above, DCP-IL-12/FLT3L plus CAR-T eradicated tumors in six out of eight mice (one mouse was killed while tumor-free), while all mice in the other groups had progressive disease (Fig. 7e–g and Extended Data Fig. 10c). Post-mortem immunofluorescence staining of the brains revealed no detectable glioma cells in the mice that were assessed as tumor-free by live imaging; interestingly, abundant CD3^+^ T cell infiltrates persisted at the site where the tumor had fully regressed (Extended Data Fig. 10d). Thus, DCP-IL-12/FLT3L synergize with GD2-specific CAR-T to eradicate the majority of intracranial gliomas in mice.

### Generation of human DCPs with antigen-presentation capacity

**Fig. 8:**
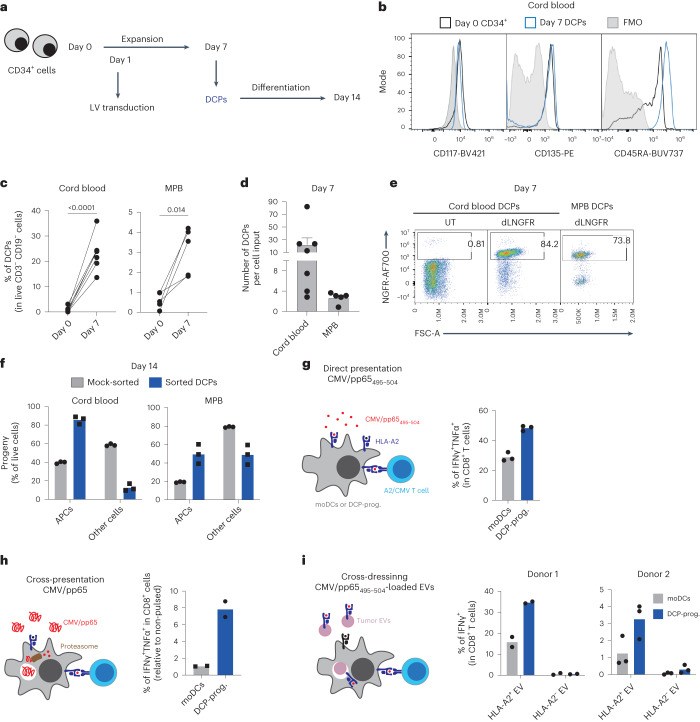
Human HSPCs are a source of DCPs with antigen-presentation capacity. **a**, Procedure to generate human DCPs from cord blood or MPB CD34^+^ cells. **b**, Representative flow cytometry histograms of CD117, CD135 and CD45RA expression of day zero cord-blood CD34^+^ cells and day seven DCPs (Lin^–^CD34^+^CD115^+^). Day seven cells were used as fluorescence minus one (FMO) staining control. **c**, Percentage of DCPs in total CD3^–^CD19^–^ live cells at days 0 and 7 after expansion (cord blood, *n* = 7 independent donors; MPB, *n* = 5). Statistical analysis by paired, two-tailed Student’s *t*-test. **d**, Number of DCPs at day 7 per input cell at day 0 (mean ± s.e.m.; cord blood, *n* = 7 independent donors; MPB, *n* = 5). **e**, Representative flow cytometry dot plots of NGFR expression of untransduced (UT) or LV-transduced (dLNGFR) DCPs, analyzed at day 7. **f**, Percentage of APCs (containing CD14^+^ monocytes, CD14^–^CD141^+^CLEC9A^+^ cDC1s, CD14^–^CD141^–^CLEC9A^–^CD1c^+^ cDC2s and CD14^–^CD141^+^CLEC9A^–^ immature DCs) after 7-day differentiation of sorted DCPs or mock-sorted cells. The data show one representative donor and three technical replicates (data points). Additional experiments with three independent donors are shown as Source Data Fig. 8. **g**, Direct antigen presentation by DCPs. The data show the percentage of IFNγ^+^TNF^+^ cells within A2/CMV/pp65_495-504_-specific CD8^+^ T cells after co-culture with CMV/pp65_495-504_ peptide-loaded HLA-A2^+^ cord-blood-derived DCP progeny (DCP-Prog.) or monocyte-derived HLA-A2^+^ DCs (moDCs). The data show one representative donor and three technical replicates (data points). Additional experiments with three independent donors are shown as Source Data Fig. 8. **h**, Antigen cross-presentation by DCPs. The data show the percentage of IFNγ^+^TNF^+^ cells within CD8^+^ A2/CMV/pp65_495-504_-specific CD8^+^ T cells after co-culture with CMV/pp65 protein-loaded HLA-A2^+^ cord-blood-derived DCP progeny or moDCs. The data show one representative donor and two technical replicates (data points). **i**, Antigen cross-dressing by DCPs. The data show the percentage of IFNγ^+^ cells within A2/CMV/pp65_495-504_-specific CD8^+^ T cells after co-culture with HLA-A2^–^ MPB-derived DCP progeny or moDCs, which were previously exposed to CMV/pp65_495-504_ peptide-pulsed melanoma extracellular vesicles (EVs). The data show two independent donors with two (donor 1) or three (donor 2) technical replicates (data points). Source data

The preclinical efficacy of murine DCPs motivated us to test whether human cord-blood CD34^+^ HSPCs would support DCP differentiation. Within 1 week, CD34^+^ cells cultured in the presence of FLT3L, IL-3, IL-6, TPO and the small molecule UM729 (ref. ^39^) expanded significantly (Fig. 8a and Extended Data Fig. 10e) and acquired more differentiated progenitor states encompassing common myeloid progenitors, granulocyte-monocyte and DC progenitors, monocyte-DC progenitors (MDPs), CDPs and pre-DCs^40^ (Extended Data Fig. 10f). Although the frequency of cells expressing CDP markers was negligible (<0.1%), the cultured cells contained a sizeable proportion (about 7%) of MDPs (Extended Data Fig. 10g). Given that MDPs are precursors of mononuclear antigen-presenting cells (APCs), including CDPs, cDC1 and cDC2 (ref. ^4^), we investigated the ability of a cell population containing MDPs, identified as Lin^–^CD34^+^CD115^+^ and provisionally termed DCPs, to generate cDCs. At day seven, the Lin^–^CD34^+^CD115^+^ DCPs expressed markers shared between CDPs and MDPs (for example, CD117/KIT, CD135 and CD45RA; Fig. 8b). These cells could be obtained from both cord blood and mobilized peripheral blood (MPB), which serves as a clinical source of CD34^+^ HSPCs^41^. However, cord blood yielded more DCPs than MPB (Fig. 8c,d). Both cord blood and MPB-derived DCPs could be efficiently transduced with an LV expressing dLNGFR (Fig. 8e), demonstrating the feasibility of LV transduction in this cell population.

To investigate the APC differentiation capacity of Lin^–^CD34^+^CD115^+^ DCPs, we sorted them from day-seven cord blood or MPB cultures and cultured them in cDC medium (containing FLT3L, GM–CSF, SCF and IFNα). As a control, we mock-sorted total day-seven cells, the majority of which were CD115^−^. After 1 week (day 14), the Lin^–^CD34^+^CD115^+^ cells had efficiently differentiated into APCs, including monocytes, cDC1, cDC2 and immature DCs, with lesser contribution to other cell types, which instead expanded in mock-sorted cell cultures (Fig. 8f). These results indicate that Lin^–^CD34^+^CD115^+^ cells can function as human DCPs.

We next assessed the antigen-presentation capacity of human DCP-derived cells (termed DCP progeny) and moDCs (Extended Data Fig. 10h). We used the cytomegalovirus (CMV) protein pp65 (or its HLA-A2-restricted peptide, pp65_495-504_) and HLA-A2-restricted, CMV-specific T cells^42^. We examined three antigen-presentation pathways: (1) presentation of pp65_495-504_ peptide-loaded HLA-A2, which mimics direct presentation; (2) cross-presentation of the pp65_495-504_ peptide endogenously processed from the native pp65 protein; and (3) antigen cross-dressing^6,7^ of DCs by extracellular vesicle-borne pp65_495-504_ HLA-A2. T cells were co-cultured in the presence of DCP progeny or moDCs that were previously exposed to the pp65_495-504_ peptide, pp65 protein or pp65_495-504_-loaded extracellular vesicles, to respectively assay direct presentation, cross-presentation and cross-dressing. For cross-dressing, we used extracellular vesicles isolated from either HLA-A2^+^ or negative human melanoma cells^43^ and DCs obtained from an HLA-A2-negative donor, with the premise that HLA-A2-negative extracellular vesicles would not activate HLA-A2-restricted T cells through cross-dressing. In each instance, the DCP progeny activated CMV-specific T cells more effectively than moDCs, although the magnitude of T cell activation varied with the donor (Fig. 8g–i; see Source Data Fig. 8). These results indicate that human DCPs, identified as Lin^–^CD34^+^CD115^+^ cells, can differentiate into a cell progeny with antigen-presentation capacity.

## Discussion

We present a cell therapy platform based on engineered DCPs that resemble naturally occurring mouse CDPs or human MDPs and possess the ability to generate cDC1 in mice. Expression of IL-12 and FLT3L by DCPs and their cDC1 progeny elicits antitumor immunity that is independent of direct T cell cytotoxicity. This approach relies on synergistic effects of IL-12—a pleiotropic cytokine that activates both innate and adaptive immune cells, including cross-primed CD8^+^ T cells^20^—and FLT3L, a crucial factor for the development and function of cDCs^18,19,44^.

The antitumoral efficacy of cytokine-armed DCPs may depend on several key features. Firstly, DCPs efficiently generate cDC1, which have crucial roles in regulating tumor immunity^1–5^. Secondly, expression of FLT3L expands endogenous cDCs. Thirdly, IL-12 activates lymphocytes and NK cells to secrete IFNγ, which in turn exerts pleiotropic functions contributing to tumor growth inhibition^11,45^. Physiological release of IL-12 by cDC1 initiates antitumor immunity^46–51^, which may explain our finding that DCPs are more effective than cytokine-armed moDCs. Moreover, we observed higher engraftment of the DCP progeny compared to moDCs in recipient mice. This is particularly relevant for achieving robust FLT3L expression, as the poor stability of this cytokine limits its bioavailability^52^, and frequent administrations of recombinant FLT3L are necessary to obtain therapeutic effects in mouse tumor models^18,53^.

The mechanisms whereby cytokine-armed DCPs initiate antitumor immunity remain to be elucidated. Surprisingly, tumor response to cytokine-armed DCPs was conserved in both *Rag1*-deficient and immunocompetent mice with depleted T cells, suggesting that T cell cytotoxicity may be dispensable for antitumor immunity. Conversely, IFNγ neutralization negated the therapeutic efficacy of DCPs. It is plausible that IL-12, when expressed by DCP-derived cDC1, stimulated IFNγ production by a variety of T cell and NK cell subsets, as shown in previous studies^54^, potentially in an antigen-independent manner^55^. Accordingly, activated NK cells were significantly elevated in tumors of DCP-treated *Rag1*^−/−^ mice. Moreover, scRNA-seq analysis revealed pervasive IFNγ signaling in both myeloid and melanoma cells of B16F10 tumors. A variety of immune cells, including CD4^+^ T helper and regulatory T cells, γδ T cells and NK cells, can produce IFNγ in tumors^45^ and contribute to immunotherapy efficacy in HLA-negative tumors that evade CD8^+^ T cell recognition^56,57^. IFNγ is crucial for immunotherapy response in both mice and patients with cancer^58–62^. It directly programs antitumoral (M1-like) TAMs^24^ and has cytostatic effects on cancer cells^63^ and endothelial cells^27^. IFNγ neutralization abated M1-like TAMs and abrogation of cancer-cell responsiveness to IFNγ suppressed therapeutic response to DCP treatment. Although IFNγ signaling in cancer cells may orchestrate feedback inhibition mechanisms that limit antitumor immunity in certain contexts^64^, our results emphasize the critical role of IFNγ signaling in cancer cells for the effectiveness of DCP therapy.

We and others have previously illustrated the therapeutic potential of myeloid cells engineered to express immune-activating cytokines, such as IFNα and IL-12, in mouse cancer models^65–69^. Earlier approaches differ from our strategy in several ways, such as the use of hematopoietic progenitors with broad myeloid-cell differentiation or the need for host conditioning to enable sustained cytokine delivery by transduced cells. Unlike previous approaches, our strategy involves transfer of DCPs, which are cDC1-committed, initiate long-lasting immune responses and persist in sizable numbers for only several days, mitigating potential safety concerns related to persistent cytokine expression. Similar to traditional moDC vaccines, repeated DCP injections may be considered in patients with cancer to enhance the durability of the immune response. Cytokine-armed DCPs significantly improved immunochemotherapy efficacy in two aggressive liver cancer models. In a mouse glioma model refractory to immune checkpoint blockade^36^, DCPs synergized with CAR-T cells to eradicate established tumors, whereas both monotherapies exhibited minor activity. This finding suggests that IL-12 deployment by DCPs may directly enhance CAR-T cells. Alternatively, DCPs may orchestrate an IFNγ-dependent, endogenous immune response that eliminates cancer cells escaping recognition and killing by CAR-T cells. These results, coupled with the feasibility of developing human DCP-like cells, motivate further preclinical studies of antigen-agnostic DCP-based therapies.

## Methods

### Ethical approvals

Studies conducted at the Swiss Federal Institute of Technology in Lausanne (EPFL; De Palma laboratory) were approved by the Veterinary Authorities of the Canton Vaud according to Swiss law (protocols VD3154, VD3154.1 and VD3785). Studies conducted at the University of Geneva (UNIGE; Migliorini laboratory; glioma models) were approved by the Veterinary Authorities of the Canton Geneva according to Swiss law (protocol VD3717c). Studies conducted at the German Cancer Research Center (DKFZ; Heikenwälder laboratory; liver cancer models) were approved by the Regierungspräsidium Karlsruhe according to German law (protocols G275/18, G5/19 and DKFZ332). All studies were compliant with the humane endpoints established in the above authorizations.

### Design and construction of LVs

To generate bicistronic constructs for the expression of either FLT3L or IL-12 in combination with a marker gene (GFP or dLNGFR), or for the co-expression of FLT3L and IL-12 without marker genes, we used the P2A peptide^70^. To co-express FLT3L and GFP, we modified an LV transfer construct containing the spleen focus-forming virus (SFFV) promoter^7^. A synthetic complementary DNA (cDNA) sequence encoding for the mouse FLT3L, in which an IgK secretion signal (MDFQVQIFSFLLISASVIMSRG) replaced the native signal peptide, was obtained from GenScript and cloned downstream to the SFFV promoter. Then, a P2A-GFP sequence was cloned downstream to the FLT3L cDNA to obtain the ‘FLT3L-P2A-GFP’ LV. To co-express IL-12 and GFP, we obtained from GenScript a synthetic cDNA sequence encoding for the single-chain bioactive murine IL-12 containing the P40 and P35 subunits separated by a linker^71^. The IL-12 sequence was then cloned upstream to the P2A-GFP sequence to obtain the ‘IL-12-P2A-GFP’ LV. To co-express IL-12 with dLNGFR or FLT3L, we obtained full-length, mouse-optimized DNA sequences from GenScript and cloned them downstream to the SFFV promoter to obtain the ‘IL-12-P2A-dLNGFR’ and ‘IL-12-P2A-FLT3L’ LVs. Monocistronic LVs expressing GFP or dLNGFR from the SFFV promoter were described previously^7^.

### LV production and titration

Third-generation self-inactivating LV particles were produced according to published protocols^72,73^. In brief, 293T cells were transiently transfected with a mix of packaging plasmids and the desired transfer construct as described previously^72,73^. Conditioned medium was collected after 48 h and 72 h and concentrated by ultracentrifugation with a Beckman ultracentrifuge^72,73^. To titrate concentrated LVs, 293T cells were transduced by serially diluted LV particles. The frequency of marker gene (GFP or dLNGFR/CD271)-positive cells was measured by flow cytometry 5–8 days after transduction, and the titer was calculated as described previously^73^. The IL-12-P2A-FLT3L LV was titrated by ELISA of the capsid protein p24 (OriGene), IL-12 (BD Biosciences) or FLT3L (Invitrogen).

### Design and production of retroviral vectors

The anti-GD2 CAR retroviral vector was generated as follows. The murine-optimized anti-GD2 scFv 14g2a was obtained from GenScript. The scFv fragment was cloned in the pMSGV retroviral vector in frame with mouse CD8a hinge and transmembrane segments, mouse 4–1BB intracellular domain and mouse CD3ζ intracellular domain. Phoenix-Eco cells were transfected with the GD2 CAR plasmid and pCL-Eco-packaging plasmid using lipofectamine (Invitrogen). Cell culture supernatant containing retroviral vector particles was collected after 48 h and 72 h and concentrated as described above for LV particles.

### Cell lines

The 293T cells were obtained from L. Naldini (San Raffaele Institute, Milan, Italy). Phoenix-Eco cells were obtained from ATCC (cat. no. CRL-3214). MC38 colorectal carcinoma cells and B16F10 melanoma cells modified to express OVA were obtained from P. Romero (University of Lausanne, Switzerland). SB28 glioma cells were generated and provided by H. Okada (University of California, San Francisco, CA, USA); these cells express both luciferase and GFP. Cell lines were cultured in DMEM (ThermoFisher) with 10% FBS (Gibco), 1% l-glutamine (Gibco) and 1% penicillin–streptomycin (Pen–Strep; ThermoFisher). While original stocks of cancer cell lines were authenticated, we did not perform further authentication in the past several years. However, the cell lines appeared authentic based on morphology and growth behavior in vitro and in vivo, especially with respect to the ability to form tumors in mice, expression of defined fluorescent or bioluminescent genes and gene expression. In particular, 293T and Phoenix-Eco cells supported high-titer LV and retroviral vector production; B16F10 cells were validated as melanoma cells by RNA-seq analysis; and SB28 cells expressed luciferase and GFP and formed invasive gliomas in mice. All cell lines were negative for *Mycoplasma* contamination in tests performed in the laboratory.

### Modification of cell lines

SB28 cells were modified to express GD2 by transduction of cancer cells with LVs encoding GD2S and GD3S synthases, as described previously^7^. GD2^+^ (transduced) cancer cells were sorted using a FACSAria II SORP (Becton Dickinson).

To generate *Ifngr1*-knockout B16F10 cancer cells, we used a previously described CRISPR–CAS9 system that does not involve stable expression of immunogenic CAS9 or resistance genes by the modified cells^74^. The guide RNA sequences were as follows:

*Ifngr1*-KO_Fw: CACCGATTAGAACATTCGTCGGTAC

*Ifngr1*-KO_Rv: CTAATCTTGTAAGCAGCCATGCAAA

The targeting plasmid was transfected in parental B16F10 cells (which express OVA) using lipofectamine (Invitrogen), and GFP^+^ cells were sorted 72 h after transfection. Cells were kept in culture and passaged until expression of GFP was lost. To isolate *Ifngr1*-knockout cells, sorted cells were stimulated with IFNγ (PeproTech; 20 ng ml^–1^) for 24 h to induce expression of H-2K^b^ on the *Ifngr1*-proficient population^7^ and stained with an antibody to H-2K^b^/SIINFEKL (BioLegend). H-2K^b^/SIINFEKL-negative cells were then sorted. To further validate loss of IFNGR expression in *Ifngr1*-knockout cells, sorted *Ifngr1*-knockout and *Ifngr1*-proficient cells were treated with IFNγ or left untreated and then stained with an anti-B2m antibody (BioLegend) to assess responsiveness to IFNγ, or lack thereof.

### Isolation of mouse BM cells

Long bones of CD45.1 or CD45.2 C57BL/6 mice were excised and decontaminated. BM cells were isolated by a pulse of high-speed centrifugation and passed through a 70 μm cell strainer to remove debris and aggregates. Red blood cells were removed with RBC Lysis Buffer (Sigma) and BM cells were filtered to obtain single-cell suspensions. BM marrow cells were washed once in complete RPMI 1640 Medium (ThermoFisher) supplemented with 10% FBS (Gibco), 1% glutamine and 1% Pen–Strep before transfer to specific media.

### Generation of mouse DCPs

DCPs were generated from mouse BM cells isolated from long bones as described above. The two-step procedure involved short-term expansion of HSPCs followed by partial differentiation under conditions that promote CDP and cDC1 lineage commitment^14^. For the expansion phase, BM cells were cultured at a density of 2 × 10^6^–3 × 10^6^ cells per ml in 10 cm plates for 2–3 days in complete RMPI medium containing recombinant murine cytokines: 100 ng ml^–1^ SCF, 40 ng ml^–1^ TPO, 50 ng ml^–1^ FLT3L, 30 ng ml^–1^ IL-3, 30 ng ml^–1^ IL-6 and 30 ng ml^–1^ IL-1b (all from PeproTech) (referred to as ‘HSPC medium’). For the differentiation phase, floating cells were then collected and cultured at a density of 2–3 × 10^6^ cells per ml in six-well plates for 4–5 days in complete RMPI medium containing 200 ng ml^–1^ FLT3L and 5 ng ml^–1^ GM–CSF (PeproTech) (cDC1 medium). The DCPs were then enriched by depleting lineage-positive cells using EasySep Mouse Hematopoietic Progenitor Cell Isolation Kit (STEMCELL Technologies).

### Differentiation of DCPs in the presence of T cell cytokines

Enriched DCPs (10^6^ cells per ml) were resuspended in ‘cDC1 medium’ (complete RMPI medium with 200 ng ml^–1^ FLT3L and 5 ng ml^–1^ GM–CSF) supplemented with various recombinant murine cytokines (IL-2, IL-12, IL-15, IL-18, IL-21, IL-23, IL-27 or none; all from PeproTech except for IL-18, IL-23 and IL-27, from BioLegend). Each cytokine was tested at the concentration of 2 ng ml^–1^, 8 ng ml^–1^ or 20 ng ml^–1^. The phenotype of DCP-derived cells was examined after 15 days of culture.

### Generation of mouse moDCs and cDC1-like cells

To generate moDCs, BM cells were cultured in complete RPMI supplemented with 100 ng ml^–1^ GM–CSF and 40 ng ml^–1^ IL-4 (referred to as ‘moDC medium’) at a concentration of 2 × 10^6^–3 × 10^6^ cells per ml for 2 days, as previously described^7,15^. Non-adherent and loosely adherent cells were gently collected, replated at a concentration of 2 × 10^6^–3 × 10^6^ cells per ml in six-well plates and cultured for four to six additional days. To generate cDC1-like cells, we used a published protocol^14^ with some modifications. In brief, BM cells were cultured in cDC1 medium at a density of 2 × 10^6^–3 × 10^6^ cells per ml in six-well plates for up to 14–18 days but adding fresh medium every 3–4 days.

### Co-culture of OT-I and OT-II T cells with antigen-loaded cDC1-like cells

BM cells were differentiated into cDC1-like cells and incubated for 4 h in 96-well U-bottom plates (5 × 10^4^ per well) in 200 µl of medium in the presence of 2 mg OVA protein (10 mg ml^–1^; vac-stova; InvivoGen) and individual interleukins (IL-2, IL-12, IL-15, IL-18, IL-21, IL-23 or IL-27). The concentration of each interleukin was 2 ng ml^–1^, 8 ng ml^–1^ or 20 ng ml^–1^ (in 200 µl). Meanwhile, OT-I CD8^+^ and OT-II CD4^+^ cells were isolated using EasySep kits (STEMCELL Technologies) and resuspended in T cell medium (complete RPMI medium with 50 μM beta-mercaptoethanol, minimal non-essential amino acids, 1 mM sodium pyruvate and 10 mM HEPES). OVA-loaded cDC1-like cells were then washed and co-cultured with OT-I or OT-II cells in T cell medium in the presence of the aforementioned interleukins. Co-culture of OVA-loaded cDC1-like cells with OT-I or OT-II cells was continued for 3 days and 5 days, respectively. T cell activation was measured by intracellular staining with antibodies against IFNγ (clone XMG1.1, BD Biosciences) using BD Golgi Stop kit (BD Biosciences).

### Mouse DCP and moDC transduction with LVs

Freshly enriched DCPs were cultured in cDC1 medium at a concentration of 1.5 × 10^6^ DCPs per ml in six-well plates and transduced 2 h later with LVs at the multiplicity of infection of 350 (determined by GFP or dLNGFR titer, or ELISA, as described above). Non-adherent or loosely attached moDCs were collected, plated in six-well plates at a concentration of 1.5 × 10^6^ moDCs per ml and transduced with LVs at the multiplicity of infection of 100. Transduction was measured 3 days after transduction by flow cytometry or ELISA. For adoptive transfer to mice, transduced cells were collected 12–14 h after transduction and resuspended in PBS before infusion.

### CAR-T cell production

Spleens from C57BL/6 mice were smashed on a 70 μm cell strainer. CD8^+^ T cells were purified using the EasySep Mouse T Cell Isolation Kit (STEMCELL Technologies), then 0.5 × 10^6^ T cells were seeded in 48-well plates in complete RPMI medium supplemented with 10% fetal calf serum (FCS) and 50 IU ml^–1^ rhIL-2 (Bio-Techne). T cells were activated with Activator CD3/CD28 Dynabeads (Gibco/ThermoFisher) at a ratio of two beads per cell. Retroviral vector transduction was conducted after 24 h in 48-well plates coated with 20 μg ml^–1^ recombinant human fibronectin (Takara Clontech). The medium was replaced on day three with medium containing 10 IU ml^–1^ rhIL-2, 10 ng ml^–1^ rhIL-7 (PeproTech) and 10 ng ml^–1^ rhIL-15 (PeproTech). Cells were passaged every second day. CAR expression was determined on day eight by flow cytometry before T cell collection for downstream experiments.

### CAR-T cell killing assay

SB28 cells, both unmodified or modified to express GD2, were seeded in 96-well plates at 5 × 10^4^ cells per well. Cells were allowed to adhere to the plate for 2 h, followed by the addition of non-transduced T cells and anti-GD2 (14g2a) mouse CAR-T cells. Three different effector:target ratios were tested: 1:1, 5:1 and 10:1. The cytotoxic assay was conducted for 72 h, then the proportion of dead cancer cells was evaluated by flow cytometry. SB28 cells were identified by GFP expression while dead cells were detected using the LIVE/DEAD Fixable Violet Dead Cell Stain Kit (Invitrogen). The percentage of specific lysis was calculated using the following formula: % specific lysis = ((% sample lysis − % control lysis)/(100 − % control lysis)) × 100, where ‘% sample lysis’ corresponds to T cells + cancer cells and ‘% control lysis’ corresponds to cancer cells alone.

### Mice

All studies used C57Bl/6 mice (CD45.2 wild type; CD45.1 wild type; *Batf3*^−/−^; *Rag1*^−/−^). C57Bl/6 (CD45.2) mice were purchased from Charles River Laboratories (France). C57Bl/6 (CD45.1), *Batf3*^−/−^ and *Rag1*^−/−^ mice were maintained as stable colonies in the EPFL mouse facility. All mice were housed and bred under specific pathogen-free conditions in the EPFL, UNIGE and DKFZ mouse facilities. Mice were housed in groups of up to five mice per cage at 18–24 °C with 40–60% humidity and maintained on a 12:12 h light:dark cycle (06:00–18:00 h). Experiments involving subcutaneous tumor models used cohorts of 6–9-week-old female mice. Glioma studies were performed twice: once in female mice and once in male mice (all 6–8 weeks old). The liver cancer studies used 8–9-week-old male mice. BM cells were isolated from female mice.

### Adoptive DC transfer studies

For adoptive transfer of DCs to tumor-free mice, DCPs, moDCs and cDC1-like cells were prepared from CD45.1 mice (or CD45.2 mice in the case of *Batf3*^−/−^ donor mice). Congenic CD45.2 mice (or CD45.1 mice in the case of *Batf3*^−/−^ donor cells) received 2 × 10^6^ cells twice (3 days apart) through the tail vein, without prior conditioning. Recipient mice were killed 4 days after the second cell dose, and the spleen was removed to analyze donor-derived cells.

For adoptive transfer of DCPs, moDCs and cDC1-like cells to tumor-bearing mice, CD45.1 or CD45.2 cells, generated and transduced as described above, were infused twice (2–3 days apart) in recipient mice (either CD45.2 or CD45.1). The DC dose and time of injection after tumor initiation was dependent on the tumor model, as shown in the figures. When LVs were used that expressed only one cytokine, transduced cells were mixed before injection through the tail vein in a 1:2 ratio for IL-12 and FLT3L-expressing cells, respectively (1 × 10^6^ IL-12-expressing cells plus 2 × 10^6^ FLT3L-expressing cells in most experiments, excluding intracranial DC delivery; see ‘intracranial glioma model’ model below). Control cells expressing marker genes only (GFP or dLNGFR) were mixed to corresponding ratios and numbers, and infused. When LVs were used that expressed both cytokines coordinately, 1 × 10^6^ cells (most experiments) or 2 × 10^6^ cells (for moDC in dose escalation study) were infused (excluding intracranial DC delivery; see ‘intracranial glioma model’ model below). Recipient mice were killed several days or weeks after the second DCP injection, depending on the tumor model.

### Subcutaneous tumor models

B16F10 and MC38 cells were passaged at least three times to obtain actively growing cells. Cancer cells were then resuspended in PBS (5 × 10^6^ cells per ml for MC38 and 2 × 10^6^ cells per ml for B16F10 cancer cells), and 100 µl of cell suspension was injected subcutaneously into the right flank of C57BL/6 mice. DCPs, moDCs and cDC1-like cells were infused on days 3 and 5 after tumor injection, except in Fig. 5a,b (days 11 and 13). The mice and tumor growth were monitored three times per week. The tumors were allowed to grow up to 1 cm^3^ in size. Upon reaching the endpoint tumor size, the experiments could continue for an additional 48 h, provided that all health parameters detailed in a health score sheet remained normal. Long (*D*) and short (*d*) tumor diameters were measured with a caliper and the tumor volume calculated using the following formula: tumor volume = ½ × *d*^2^ × *D*.

### Liver tumor models

To obtain *Kras*^G12D^; *Trp53*^−/−^ liver tumors, we used HDTVi of previously described oncogenic plasmids^75^. To obtain mice with *Kras*^G12D^; *Trp53*^−/−^ liver tumors, we prepared 2 ml of 0.9% NaCl solution containing the following plasmids (for one mouse): 5 µg of pT3-EF1a-Kras^G12D^-IRES-GFP (gift from D. Tschaharganeh, DKFZ, Heidelberg), 10 µg of px330-sg-tp53 (gift from T. Jacks, MIT, Boston; addgene, plasmid no. 59910) and 1.25 µg of pCMV-sleeping beauty 13 (pCMV-SB13) transposase-encoding plasmid^76^ (gift from D. Tschaharganeh, DKFZ, Heidelberg). To obtain mice with *Myc*; *Trp53*^−/−^ liver tumors, we prepared 2 ml of 0.9% NaCl solution containing the following plasmids (for one mouse): 5 µg of pT3-EF1a-MYC-IRES-luciferase^34^ (addgene, plasmid no. 129775), 10 µg of px330-sg-tp53 and 1.25 µg of pCMV-SB13 transposase-encoding plasmid. The plasmid mix was delivered by HDTVi with its volume adjusted to 10% of the body weight of each mouse.

Both HDTVi models were initiated in male C57BL/6 mice at the age of 8–9 weeks. Seven days after HDTVi, the liver was imaged by magnetic resonance tomography (MRT) for the *Kras*^G12D^; *Trp53*^−/−^ model, and by in vivo imaging system (IVIS) analysis of luciferase for the *Myc*; *Trp53*^−/−^ model. For MRT (*Kras*^G12D^; *Trp53*^−/−^ model), anesthetized mice were screened using a Pharmascan 7T MRT (Bruker) with ParaVision 5.1 software in FLASH scan mode without fat suppression, using an echo-time of 2.2 ms for out-phase and 2.9 ms for in-phase. For IVIS, imaging was performed using an IVIS Spectrum system (PerkinElmer). Mice were injected intraperitoneally with fresh d-luciferin (150 mg kg^–1^; ThermoFisher Scientific) and anesthetized with 3% isoflurane. Ten minutes after luciferin injection, mice were scanned in the IVIS imaging chamber. The luciferase signal was analyzed with IVIS software (v4.7.3; PerkinElmer). Randomization was performed based on MRT or IVIS imaging results.

Transduced DCPs were infused in two doses on days 11 and 13 after HDTVi. DCPs were mixed before injection (1 × 10^6^ IL-12-expressing cells and 2 × 10^6^ FLT3L-expressing cells). For each model, one mouse cohort was killed on day 23 (*Kras*^G12D^; *Trp53*^−/−^) or day 21 (*Myc*; *Trp53*^−/−^) for flow cytometry. Another cohort was monitored for survival for up to 90 days. The number of liver nodules was determined post-mortem by inspection under a stereoscope. Termination criteria were defined by authority-confined endpoints (morbidity; non-physiological posture; indication of jaundice, cramps, or emaciation; weight loss of more than 20% of the initial weight).

### Intracranial glioma model

For the intracranial glioma model, 6–8-week-old C57BL/6 mice were implanted intracranially with SB28 cells using a stereotaxic apparatus. Mice were anesthetized with isofluorane and received subcutaneous injections of 2 μg of buprenorphinum (Bupaq; Streuli) in 100 μl of PBS and 50 μl of lidocaine 1% (Streuli) before surgery. Next, 1.6 × 10^3^ SB28 cells were injected in 2 μl PBS in the pallidum. Seven days after tumor engraftment, we measured the tumor burden by IVIS (PerkinElmer) and randomized the mice according to tumor burden. Eight and eleven days after tumor engraftment, the mice received transduced DCPs intravenously (either 1 × 10^6^ IL-12-expressing cells plus 2 × 10^6^ FLT3L-expressing cells, or 1 × 10^6^ DCPs co-expressing IL-12 and FLT3L). Additionally, 10 days after tumor engraftment, mice received 0.5 × 10^6^ transduced DCPs and 1 × 10^6^ CAR-T cells intracranially in the same location as the injected tumor. Mice were monitored three times per week, imaged by IVIS two times per week and killed when meeting authority-confined endpoints (15% weight loss over 1 week; compromised ability to walk, eat or drink; dyspnea; hunched posture; or lethargic behavior).

### Treatment of mice with cell-depleting or neutralizing antibodies

Cell-depleting or neutralizing antibodies were administered starting 1 day before DCP transfer, followed by injection twice per week until the end of the experiment. We used the following monoclonal antibodies: CD8a^+^ cell-depleting antibody (12 mg kg^–1^; clone 53–6.7, rat IgG2a, Bio X Cell), CD4^+^ cell-depleting antibody (10 mg kg^–1^; clone GK1.5, rat IgG2b, Bio X Cell), CSF1R^+^ cell-depleting antibody (30 mg kg^–1^; clone AFS98, rat IgG2a, Bio X Cell), NK1.1^+^ cell-depleting antibody (20 mg kg^–1^; clone PK136, rat IgG2a, InVivoPlus) and IFNγ-neutralizing antibody (12 mg kg^–1^; clone XMG1.2, rat IgG1, Bio X Cell). All antibodies were prepared in sterile PBS (100 μl) and injected intraperitoneally.

### Treatment of mice with cisplatin and PD-1 blocking antibodies

For the treatment of MC38 tumor-bearing mice, cisplatin (5 mg kg^–1^) was administered once on day eight after tumor injection. The mice then received a PD-1 blocking antibody (10 mg kg^–1^; rat IgG2a, clone RMPI-14, BE0146, Bio X Cell) or rat IgG2a isotype control antibody (10 mg kg^–1^; clone 2A3, BE0089, Bio X Cell) twice per week starting 1 day after DCP transfer. For the treatment of liver tumor-bearing mice, cisplatin (5 mg kg^–1^) was injected intraperitoneally in mice on day nine after HDTVi. These mice were also treated with a PD-1 blocking antibody or control IgG2a (3 mg kg^–1^) three times per week starting 1 day after DCP transfer. Cisplatin and antibodies were prepared in sterile PBS (100 μl) and injected intraperitoneally.

### Flow cytometry analysis of mouse cells

Tissue samples were processed as described in the Reporting Summary. Single-cell suspensions were incubated in PBS with Fc block (1:100; BD Biosciences) and fixable live–dead colors, and stained with antibodies (see Reporting Summary and Supplementary Table 1). In experiments with non-fixable live–dead colors, cells were resuspended in live–dead color 7-AAD (1 µg ml^–1^; BioLegend) or DAPI (0.1 µg ml^–1^; Sigma-Aldrich) before acquisition by flow cytometer. Stained samples were analyzed with the following flow cytometry machines: BD LSRFortessa (Becton Dickinson); BD LSR II SORP (Becton Dickinson); and Attune NxT (Invitrogen). Analysis of flow cytometry data used FlowJO v10.1. Immune cell populations were identified as indicated in the Reporting Summary and Supplementary Figs. 1–6.

### Immunofluorescence staining of tumor and liver

Freshly isolated tumors and livers were snap-frozen in OCT Compound and stored at −80 °C. Tissue sections were fixed in methanol for 20 min at −20 °C, washed and incubated in PBS with Fc block, 1% BSA and 5% FBS for 1 h at room temperature (20–25 °C). Sections were incubated overnight at 4 °C in blocking solution containing primary antibodies, followed by staining with secondary antibodies in some cases (see Reporting Summary and Supplementary Table 1). After staining, nuclei were labeled with DAPI. Image acquisition used an Axioscan microscope (Zeiss) as detailed in the Reporting Summary.

### Immunofluorescence staining of brain

Upon euthanasia, mice were perfused by cardiac injection of PBS. Whole brains were removed, placed in OCT and snap-frozen. Sections of 10 μm were fixed in 80% methanol. Following incubation in Tris-NaCl-blocking buffer, slides were incubated overnight with the antibodies listed in the Reporting Summary and Supplementary Table 1. Slides were mounted with Fluoromount and DAPI (Invitrogen). Images were acquired with an Axioscan microscope (Zeiss).

### Generation and transduction of human DCPs

CD34^+^ cells purified from cord blood were purchased from STEMCELL Technologies. CD34^+^ cells purified from plerixafor and G-CSF-MPB were purchased from STEMCELL Technologies and Lonza. CD34^+^ cells were cultured in StemSpan SFEMII medium (STEMCELL Technologies) with StemSpan CD34^+^ Expansion Supplement (STEMCELL Technologies) and 1 μM UM729 (STEMCELL Technologies) for 7 days at a concentration of 5 × 10^4^ cells per ml in U-bottom 96-well plates (medium was replaced every 2–3 days). Day-seven cultures contained CD34^+^CD115^+^ cells (comprising DCPs) and additional progenitor-cell populations identified as indicated in the Reporting Summary. To transduce human DCPs, day-one CD34^+^ cells were transferred to retronectin (Takara) coated wells and dmPGE2 (STEMCELL Technologies) was added to a final concentration of 10 μM. After 2 h, the cells were transduced with a dLNGFR-encoding LV^7^ at the multiplicity of infection of 300. The medium was replaced on the following day, and the cells were cultured for five additional days.

### Purification of human DCPs and differentiation into DCP-derived DCs and APCs

Day-seven cultures were processed to FACS-sort CD34^+^CD115^+^ DCPs (or mock-sorted) using a BD FACSAria II SORP apparatus. Sorted DCPs were cultured in StemSpam SFEMII medium (STEMCELL Technologies) supplemented with 50 units per ml of penicillin (Gibco), 50 μg ml^–1^ streptomycin (Gibco), 20 ng ml^–1^ hGM-CSF, 100 ng ml^–1^ hFLT3L, 20 ng ml^–1^ hSCF (all from PeproTech) and 1,000 IU ml^–1^ hIFNa2b (InvivoGen) for 7 days. Day-14 cultures contained differentiated cell populations identified as indicated in the Reporting Summary.

### Generation of human moDCs

Blood from healthy donors was obtained from the Blood Transfusion Center (University of Lausanne, Switzerland) under Project P_297. Peripheral blood mononuclear cells were isolated by density gradient centrifugation on Lymphoprep (STEMCELL Technologies). To generate moDCs, CD14^+^ cells were isolated with magnetic beads (Miltenyi) and cultured in RPMI 1640 containing 10% FBS, 100 U ml^–1^ penicillin, 100 μg ml^–1^ streptomycin, 2 mM glutamine, 50 ng ml^–1^ hGM-CSF and 50 ng ml^–1^ hIL4 (PeproTech; 200-04), at a density of 10^6^ cells per ml for 7 days.

### Human T cell stimulation assays

The A2/CMV/pp65_495-504_-specific CD8^+^ T cell line^42^ was provided by D. Speiser (University of Lausanne, Switzerland) and kept in culture in 8% human serum (from AB^+^ donors; Blood Transfusion Center, Lausanne), 100 U ml^–1^ penicillin, 100 μg ml^–1^ streptomycin, 2 mM glutamine, 1% NEAA (Gibco), 1 mM sodium pyruvate (Gibco) and 55 μM 2-mercaptoethanol (Gibco), in RPMI 1640 supplemented with 150 U ml^–1^ hIL-2 (PeproTech).

To evaluate antigen presentation, HLA-A2^+^ moDCs or DCP-derived DCs and APCs (DCP-progeny) were pulsed with 1 μg ml^–1^ CMV/pp65_495-504_ peptide (provided by the Peptide & Tetramer Core Facility; Ludwig Institute for Cancer Research, Lausanne) for 1 h at 37 °C. After washing, cells were co-cultured with A2/CMV/pp65_495-504_-specific CD8^+^ T cells at a 1:1 ratio in RPMI 1640 containing 10% FBS, 100 U ml^–1^ penicillin and 100 μg ml^–1^ streptomycin. After 30 min at 37 °C, brefeldin A (1:1000; BD Biosciences, GolgiPlug) was added and cells were further cultured overnight before flow cytometry analysis. To assess cross-presentation, HLA-A2^+^ moDCs or DCP-progeny were pulsed with 10 μg ml^–1^ CMVpp65 protein (Abcam) for 2 h at 37 °C before adding A2/CMV/pp65_495-504_-specific CD8^+^ T cells at a 1:1 ratio. Co-cultures were kept at 37 °C overnight and for another 4 h in the presence of brefeldin A before flow cytometry analysis. To assess cross-dressing, extracellular vesicles isolated from human melanoma cell lines^43^ were directly pulsed with 1 μg ml^–1^ CMV/pp65_495-504_ peptide for 1 h at 37 °C and washed with PBS before adding them to HLA-A2^−^ moDCs or DCP-progeny at 1 μg ml^–1^ concentration in RPMI 1640 containing 5% extracellular-vesicle-depleted FBS, 100 U ml^–1^ penicillin and 100 μg ml^–1^ streptomycin and glutamine. Extracellular vesicles were pulsed overnight at 37 °C and, after two washes, A2/CMV/pp65_495-504_-specific CD8^+^ T cells were added to the cells at a 1:1 ratio. After 30 min at 37 °C, brefeldin A was added and cells were cultured for another 5 h before flow cytometry analysis. As negative controls, we used non-pulsed moDCs or DCP-derived DCs and APCs, or T cells alone. As a positive control, T cells were stimulated with 10 ng ml^–1^ PMA and 500 ng ml^–1^ ionomycin.

### scRNA-seq

After filtration through a 40 μm Flowmi strainer (Bel-Art), single-cell tumors were resuspended in PBS with 0.04% BSA, checked for the absence of doublets or aggregates and loaded into a Chromium Single Cell Controller (10× Genomics, Pleasanton) in a chip together with beads, master mix reagents (containing reverse transcriptase and poly-dT primers) and oil to generate single-cell-containing droplets. Single-cell expression libraries were prepared using Chromium Single-Cell 3′ Library & Gel Bead Kit v3.1 (PN-1000268; protocol CG000315, Rev C). Quality control was performed with a TapeStation 4200 (Agilent) and QuBit dsDNA high-sensitivity assay (ThermoFisher). Sequencing libraries were loaded onto an Illumina HiSeq 4000 paired-end flow cell and sequenced using read lengths of 28 nt for read1 and 91 nt for read2, at a depth of about 35 k reads per cell. Cell Ranger Single-Cell Software Suite v6.1.1 was used to perform sample demultiplexing, barcode processing and 3′ gene counting using 10× Genomics custom annotation of murine genome assembly mm10. After mapping with Cell Ranger 7.0 (X, with parameters force = 15,000) on the mouse reference refdata-gex-mm10-2020-A, cells were considered for further processing using Seurat (v4.1.1). Using Seurat (v4.1.1), cells with more than 20% mitochondrial content were removed; only cells in which at least 200 genes were expressed were retained; and only genes detected in at least ten cells were retained, for a final set of 1,524 cells and 17,901 genes. Samples were independently log-normalized and integrated using 4,000 most variable features. Unsupervised clustering was performed by applying the graph-based clustering approach and Louvain algorithm, and uniform manifold approximation and projection dimensional reduction was performed based on the previously computed neighbor graph using the top 30 principal components. Manual annotation based on selected markers was used to annotate cell clusters by unsupervised clustering. Wilcoxon test statistics were used to examine differences between DCP-treated and control samples at the cell level. Overrepresentation was performed on statistically significant genes using the Hallmark and Reactome gene sets collection with ClusterProfiler (v4.4.1), using a hypergeometric test followed by Benjamini–Hochberg *P* value correction.

### Bulk TCR sequencing

Sample preparation and TCR-seq were performed as described^77^, with some modifications as follows. TCR products were purified, quantified and loaded on a NextSeq instrument (Illumina) for the deep sequencing of the TCRβ chain. The TCR sequences were further processed using ad hoc Perl scripts to: (1) pool all TCR sequences coding for the same protein sequence; (2) correct for amplification and sequencing errors using 9mers UMI; (3) filter out all out-frame sequences; and (4) determine the abundance of each distinct TCR sequence. TCRs with a single read were removed for the analysis. TCR clones that were considered out of frame were filtered out from the datasets. Analysis of the TCR clones was performed by using the Immunarch library in R v4.2.1. TCR diversity was estimated by computing the TCR richness of each tumor, defined as the number of unique clonotypes in each dataset. Tumors of mice treated with DCPs were pooled together, and TCR richness was compared with tumors of mice that did not receive DCPs. The proportion of each V gene was compared between the two groups. Tumors were correlated based on V gene usage using the Jansen–Shannon divergence and clustered by multidimensional scaling and *k*-means clustering.

### Statistics and reproducibility

Studies involving independent cohorts of mice were typically performed once, with several exceptions stated in the figure legends. No specific statistical tests were used to predetermine the sample size, and our previous experience with different tumor models provided guidance. Based on these considerations, we typically employed experimental cohorts of five to ten mice. Studies conducted in parallel may share selected mouse cohorts to limit the number of experimental mice. Consequently, some datasets may be shown more than one time in separate figures to facilitate presentation of the data (for example, Fig. 3g and Extended Data Fig. 7m; Fig. 4j and Extended Data Fig. 5b). Studies involving human primary blood-derived cells used several independent donors in experiments aimed to characterize key properties of the cells (for example, DCP expansion and yield). In those studies, we used five to seven different donors to verify reproducibility of the key results across independent donors. Further studies that aimed to evaluate qualitative differences (for example, behavior of moDCs and DCPs) used only one to three independent donors, and statistical analyses were not performed.

Tumor-bearing mice were randomized before treatment by allocating mice to alternate treatment groups. Endpoints for experiments with mice were selected in accordance with institutional-approved criteria; fixed time points of analysis shown in the figures indicate time elapsed from tumor injection. The investigators were blinded when acquiring tumor volumetry data (both at randomization and endpoint of analysis), flow cytometry data and immunofluorescence staining data, but were not blinded when analyzing flow cytometry data. In some cases, selected samples were excluded from specific analyses because of technical flaws during sample processing or data acquisition; this was the case, for example, when analyzing tissue samples that did not provide sufficient numbers of cells for multi-panel flow cytometry. Outliers were not excluded from the analyses.

Graphs were generated and statistical analyses performed with Prism (GraphPad Software). Error bars indicate s.e.m. unless indicated otherwise. The number of biological (non-technical) replicates and applied statistical analysis are indicated in the figure legends. In brief, comparison between two unpaired groups was performed by the non-parametric Mann–Whitney test. For multiple comparisons involving one variable, one-way ANOVA was performed followed by Tukey’s multiple comparison test, unless otherwise stated in the figure legends. Simultaneous analysis of two variables (tumor growth over time) among multiple groups was performed by two-way ANOVA followed by Tukey’s (three groups or more) or Sidak’s (two groups) multiple comparison test. Other statistical tests were applied in selected cases, as detailed in the figure legends.

### Reporting summary

Further information on research design is available in the Nature Portfolio Reporting Summary linked to this article.

### Supplementary information

Supplementary Information Supplementary Figs. 1–7 and Supplementary Table 1

Reporting Summary

### Source data

Source Data Fig. 1 Statistical Source Data

Source Data Fig. 2 Statistical Source Data

Source Data Fig. 3 Statistical Source Data

Source Data Fig. 4 Statistical Source Data

Source Data Fig. 5 Statistical Source Data

Source Data Fig. 6 Statistical Source Data

Source Data Fig. 7 Statistical Source Data

Source Data Fig. 8 Statistical Source Data

Source Data Extended Data Fig. 1 Statistical Source Data

Source Data Extended Data Fig. 2 Statistical Source Data

Source Data Extended Data Fig. 3 Statistical Source Data

Source Data Extended Data Fig. 4 Statistical Source Data

Source Data Extended Data Fig. 5 Statistical Source Data

Source Data Extended Data Fig. 6 Statistical Source Data

Source Data Extended Data Fig. 7 Statistical Source Data

Source Data Extended Data Fig. 8 Statistical Source Data

Source Data Extended Data Fig. 9 Statistical Source Data

Source Data Extended Data Fig. 10 Statistical Source Data

## Data Availability

All reagents used in this study are either commercially available or can be made available from the corresponding author upon reasonable request. The scRNA-seq data have been deposited in Gene Expression Omnibus (GEO) with GEO accession GSE228014. TCR-seq data have been deposited in GEO with GEO accession GSE228161. No custom code was generated. Source data are provided with this paper.
