# Supplementary material for: Cytokine-armed dendritic cell progenitors for antigen-agnostic cancer immunotherapy
Source: Nat Cancer. 2023 Nov 23;5(2):240–61. doi: 10.1038/s43018-023-00668-y (PMC10899110; doi:10.1038/s43018-023-00668-y)
Supplement: Supplementary file 1 — Supplementary Figs. 1–7 and Supplementary Table 1 [file 43018_2023_668_MOESM1_ESM.pdf]

nature cancer

Article

https://doi.org/10.1038/s43018-023-00668-y

Cytokine-armed dendritic cell progenitors for antigen-agnostic cancer immunotherapy

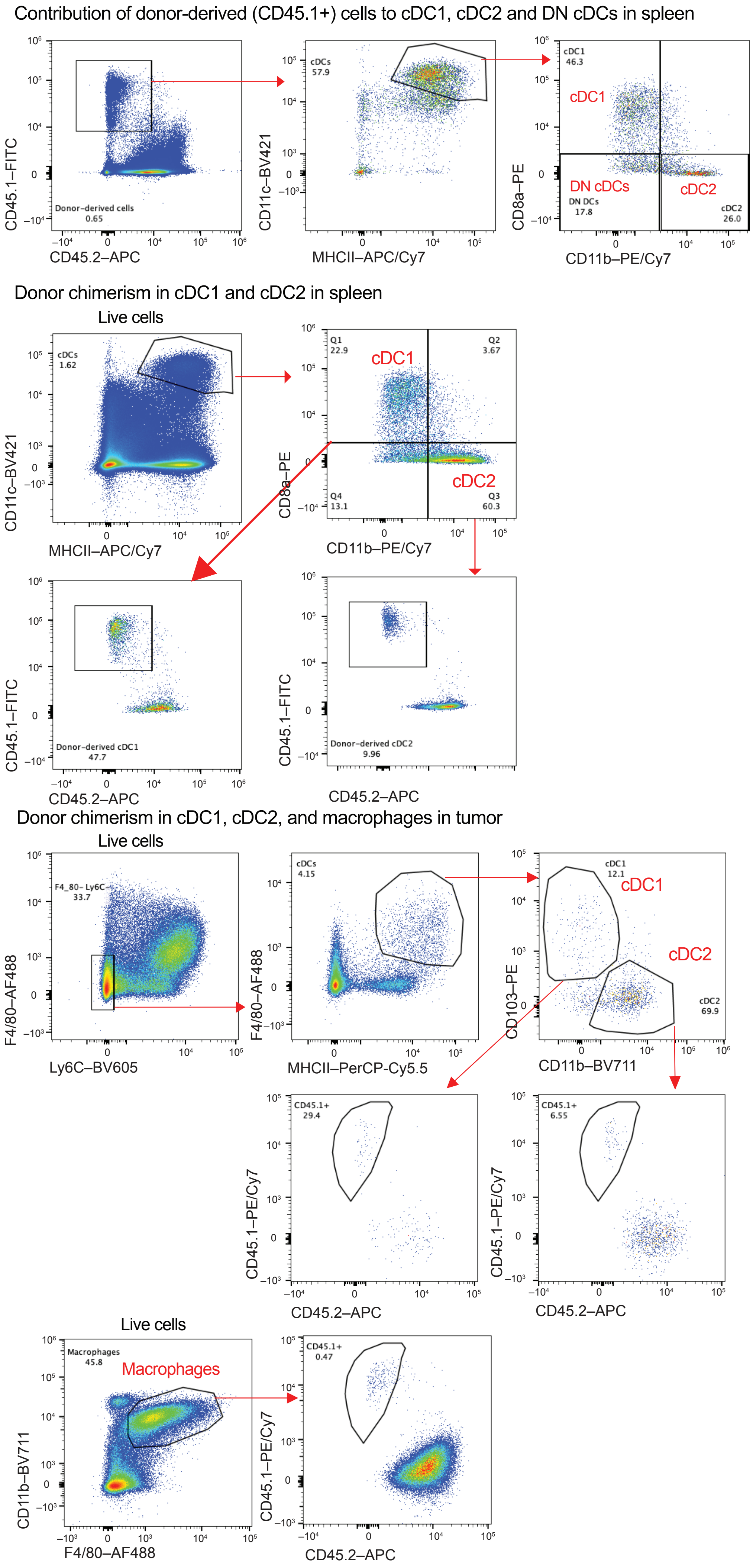

Supplementary Fig. 1: Gating strategy to identify donor chimerism in spleen and tumor.

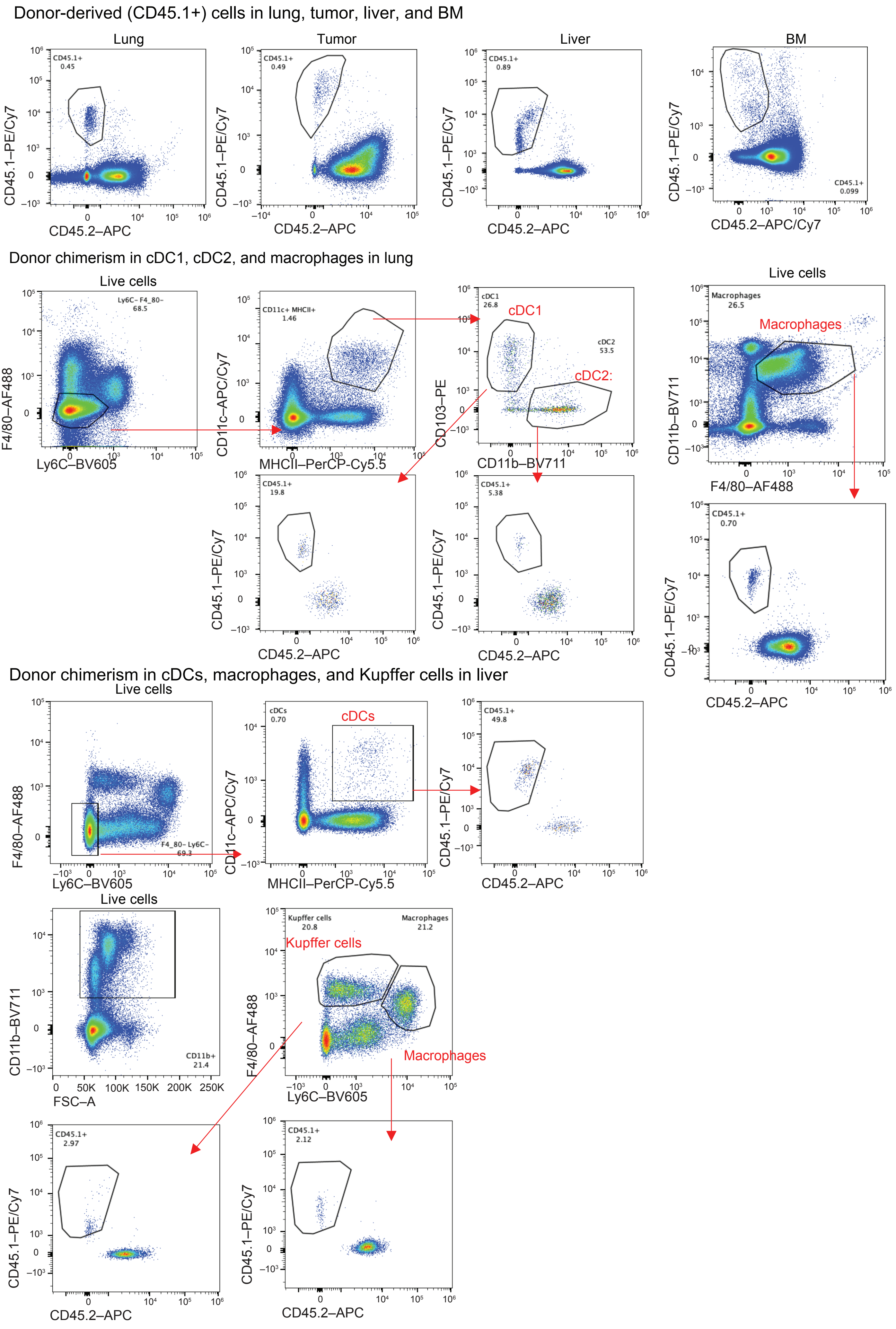

Supplementary Fig. 2: Gating strategy to identify donor chimerism in lung and liver.

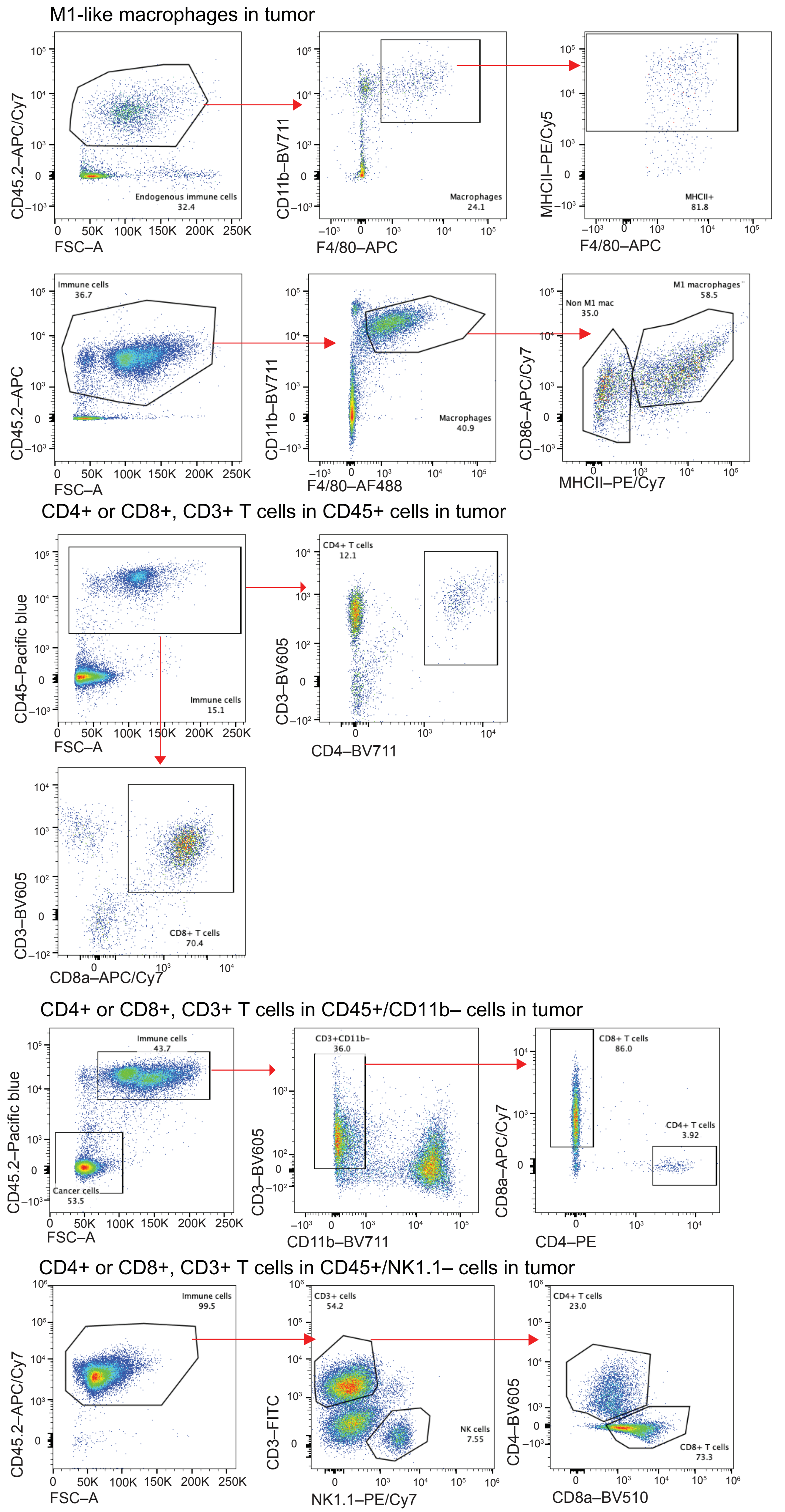

Supplementary Fig. 3: Gating strategy to identify T cells and macrophages in tumor.

Activated or exhausted CD8+ T cells in tumor

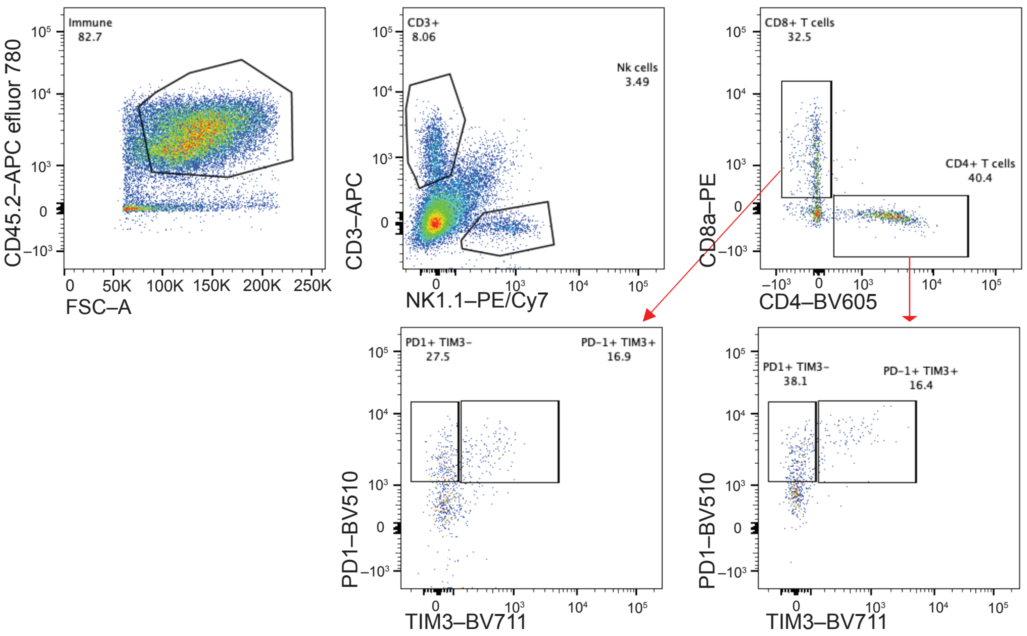

NK1.1+/CD3- or NK1.1+/CD11b- NK cells or NKT cells in CD45+ cells

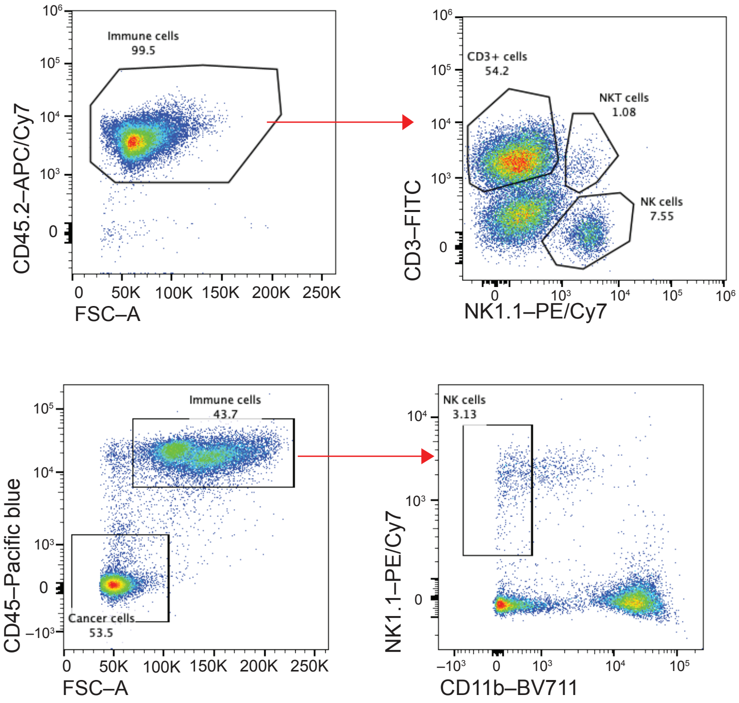

Supplementary Fig. 4: Gating strategy to identify T cell activation/exhaustion and NK/NKT cells in tumor.

CD8+ or CD4+ T effector cells in tdLN

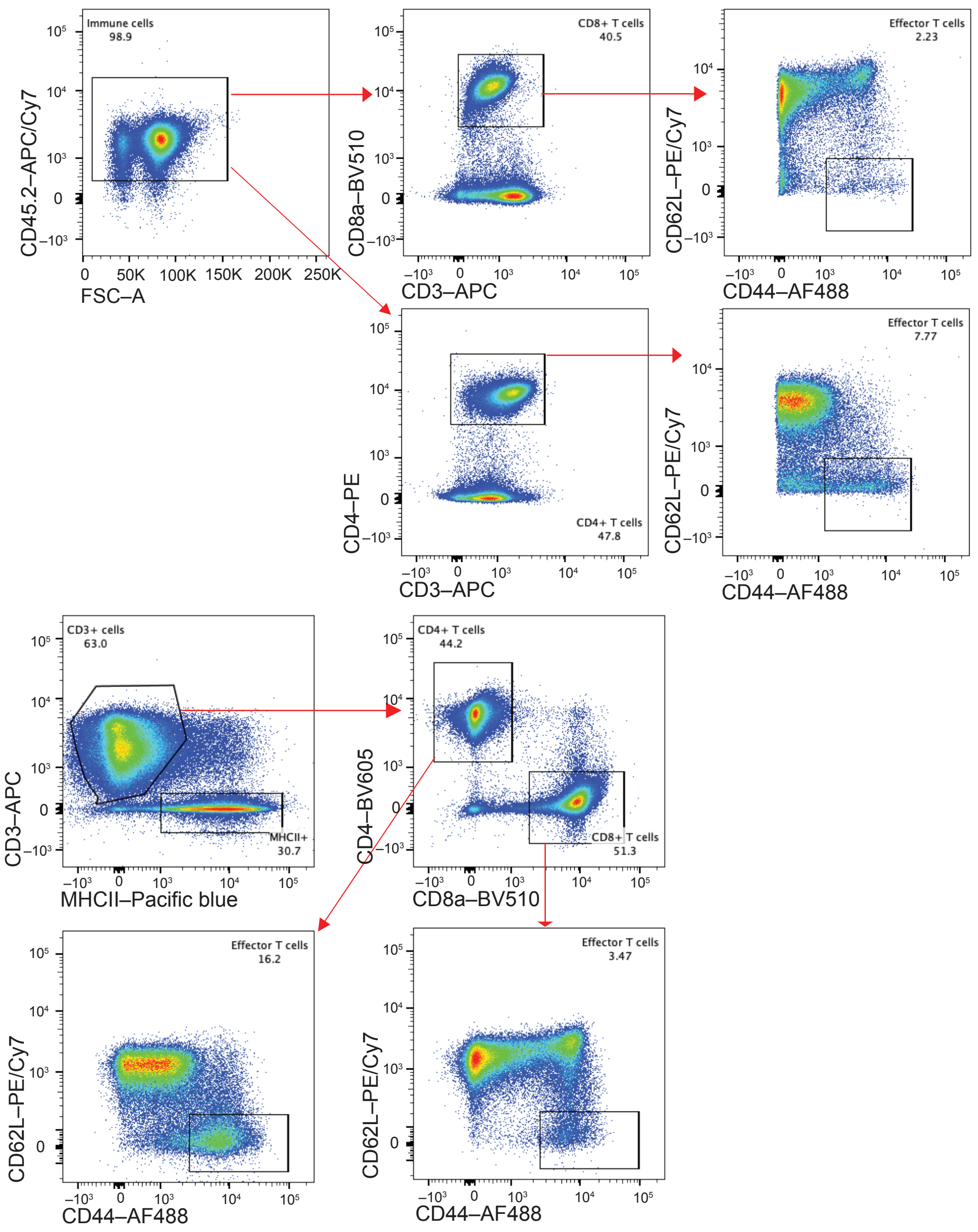

Myeloid cells in liver

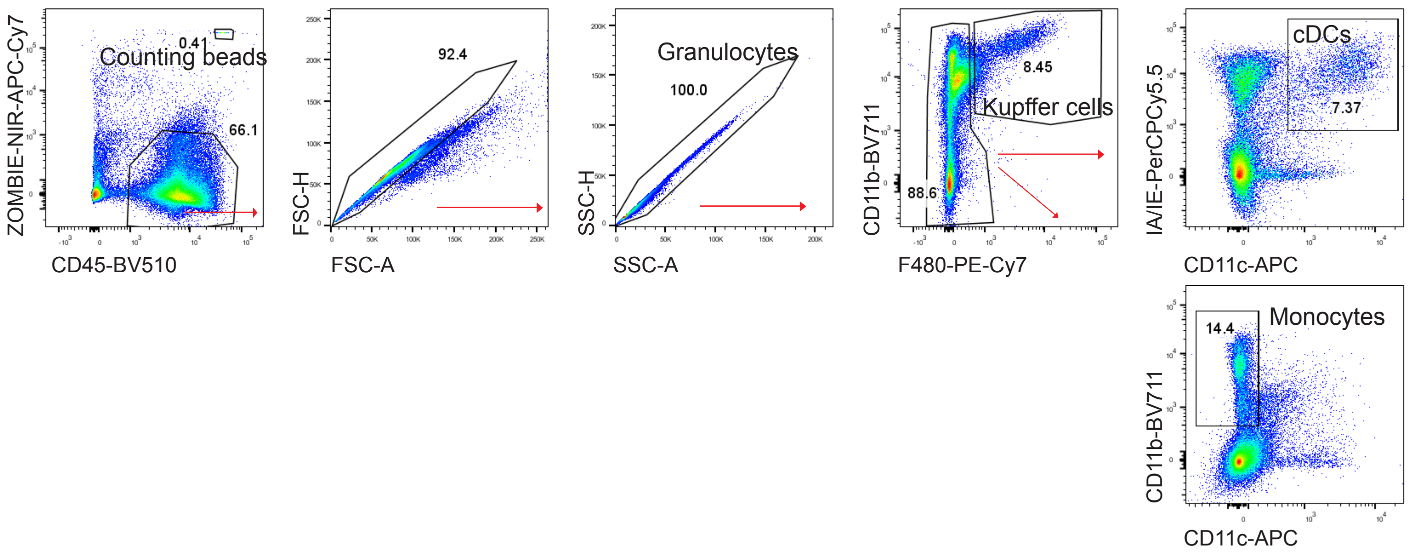

Supplementary Fig. 5: Gating strategy to identify T cells with effector phenotype in tdLN and Myeloid cells in liver.

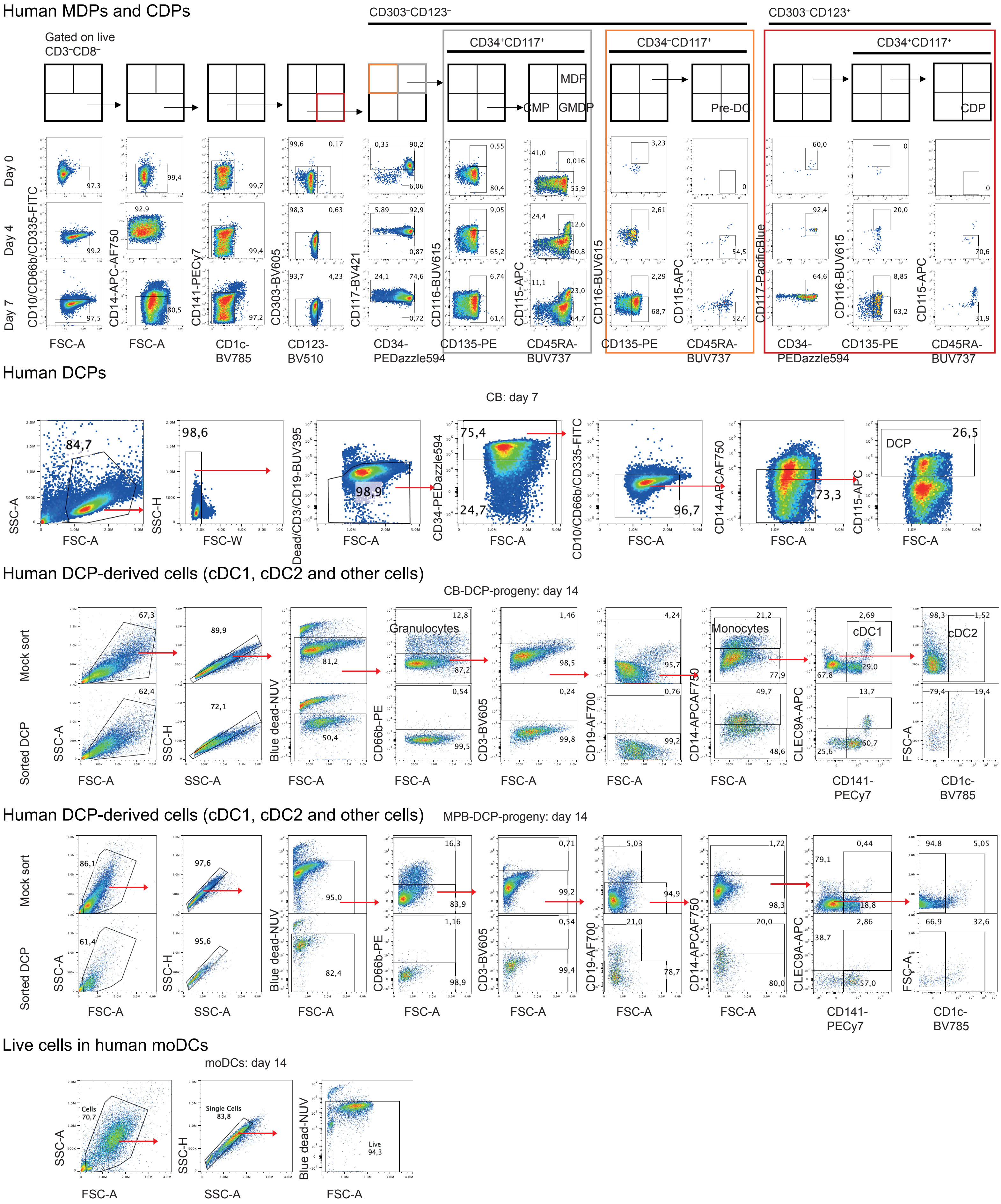

Supplementary Fig. 6: Gating strategy to identify human DCPs, other progenitors and differentiated DCs.

a

cDC1 culture (2 weeks)

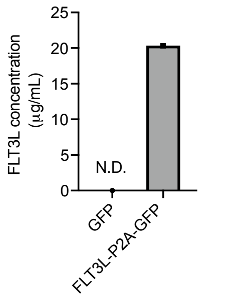

Sample	GFP	FLT3L-P2A-GFP
ng/ml	0	20.34

b

cDC1 culture (2 weeks)

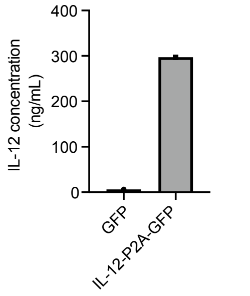

Sample	GFP	IL-12-P2A-GFP
ng/ml	6	297

c

FLT3L secretion

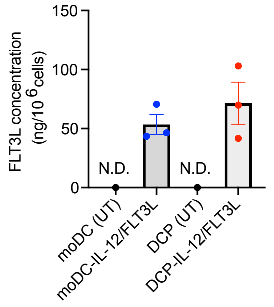

Sample	moDC UT	moDC-IL-12/FLT3L	DCP UT	DCP-IL-12/FLT3L
ng/ml	0	46.40	0	69.80
		43.45		41.64
		70.60		103.06

IL-12 secretion

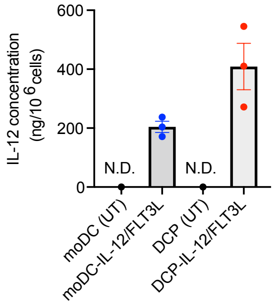

Sample	moDC UT	moDC-IL-12/FLT3L	DCP UT	DCP-IL-12/FLT3L
ng/ml	0	203.96	0	410.23
		171.48		271.58
		237.37		545.08

Supplementary Fig. 7: Quantification of FLT3L and IL-12 in medium conditioned by transduced DCPs or moDCs, according to ELISA. **a,** ELISA of FLT3L secreted by transduced HSPC-derived cells. **b,** ELISA of IL-12 secreted by transduced HSPC-derived cells. **c,** ELISA of FLT3L and IL-12 secreted by DCP-IL-12/FLT3L and moDC-IL-12/FLT3L. Data indicate mean values +/- SEM. Individual dots indicate independent cell cultures transduced at the same time.

Supplementary Table 1

FLOW CYTOMETRY

Antigen	Fluorochrome	Reactivity	Clone	Provider	Reference	Dilution used	Validation
BDCA2/CD303	Brillant Violet 605	Human	201A	BioLegend	354223	1:100	https://www.biolegend.com/en-us/products/brilliant-violet-605-anti-human-cd303-bdca-2-antibody-13062
BDCA3/CD141	PE-Cy7	Human	M80	BioLegend	344109	1:400	https://www.biolegend.com/en-us/products/pe-cyanine7-anti-human-cd141-thrombomodulin-antibody-8221
BDCA1/CD1c	Brillant Violet 785	Human	L161	BioLegend	331543	1:100	https://www.biolegend.com/en-us/products/brilliant-violet-785-anti-human-cd1c-antibody-17375
CD10	FITC	Human	HI10a	BioLegend	982204	1:100	https://www.biolegend.com/en-us/products/fitc-anti-human-cd10-antibody-14046
CD103	Brillant Violet 605	Mouse	2E7	BioLegend	121433	1:100	https://www.biolegend.com/ja-jp/products/brilliant-violet-605-anti-mouse-cd103-antibody-13558
CD103	Brillant Violet 711	Mouse	2E7	BioLegend	121435	1:100	https://www.biolegend.com/en-us/products/brilliant-violet-711-anti-mouse-cd103-antibody-14411?GroupID=BLG4646
CD103	FITC	Mouse	2E7	BioLegend	121420	1:200	https://www.biolegend.com/en-ie/products/fitc-anti-mouse-cd103-antibody-7053?GroupID=GROUP20
CD103	PE	Mouse	2E7	BioLegend	121406	1:100	https://www.biolegend.com/en-us/products/pe-anti-mouse-cd103-antibody-3574?GroupID=GROUP20
CD115	APC	Mouse	AFS98	ThermoFisher Scientific	17-1152-80	1:100	https://www.thermofisher.com/antibody/product/CD115-c-fms-Antibody-clone-AFS98-Monoclonal/17-1152-82
CD115	APC	Human	9-4D2-1E4	BioLegend	347305	1:50	https://www.biolegend.com/en-us/products/apc-anti-human-cd115-csf-1r-antibody-8783
CD117	Brillant Violet 421	Human	104D2	BioLegend	313215	1:50	https://www.biolegend.com/en-us/products/brilliant-violet-421-anti-human-cd117-c-kit-antibody-7348
CD11b	Brillant Violet 510	Mouse	M1/70	BioLegend	101263	1:100	https://www.biolegend.com/en-us/products/brilliant-violet-510-anti-mouse-human-cd11b-antibody-7993
CD11b	Brillant Violet 650	Human/Mouse	M1/70	BioLegend	101239	1:400	https://www.biolegend.com/en-us/products/brilliant-violet-650-anti-mouse-human-cd11b-antibody-7638
CD11b	Brillant Violet 711	Human/Mouse	M1/70	BioLegend	101241	1:200	https://www.biolegend.com/en-us/products/brilliant-violet-711-anti-mouse-human-cd11b-antibody-7927
CD11b	PE-Cy7	Human	M1/70	eBioscience	25-0112-82	1:400	https://www.thermofisher.com/antibody/product/CD11b-Antibody-clone-M1-70-Monoclonal/25-0112-82
CD11c	APC	Mouse	N418	BioLegend	117309	1:200	https://www.biolegend.com/de-at/products/apc-anti-mouse-cd11c-antibody-1813
CD11c	APC-Cy7	Mouse	N418	BioLegend	117324	1:100	https://www.biolegend.com/fr-ch/products/apc-cyanine7-anti-mouse-cd11c-antibody-3931?GroupID=BLG11937
CD11c	Brillant Violet 421	Mouse	N418	BioLegend	117330	1:100	https://www.biolegend.com/fr-ch/products/brilliant-violet-421-anti-mouse-cd11c-antibody-7149?GroupID=BLG11937
CD11c	Brillant Violet 650	Mouse	N418	BioLegend	117339	1:100	https://www.biolegend.com/en-us/products/brilliant-violet-650-anti-mouse-cd11c-antibody-8840?GroupID=BLG11937
CD11c	Brillant Violet 650	Human	3.9	BioLegend	301637	1:50	https://www.biolegend.com/en-us/products/brilliant-violet-650-anti-human-cd11c-antibody-8642
CD11c	PE-Dazzle594	Human	3.9	BioLegend	301641	1:100	https://www.biolegend.com/en-us/products/pe-dazzle-594-anti-human-cd11c-antibody-10234
CD116	BUV615	Human	hGMCSFR-M1	BD Biosciences	751535	1:200	https://www.bdbiosciences.com/en-us/products/reagents/flow-cytometry-reagents/research-reagents/single-color-antibodies-ruo/buv615-mouse-anti-human-cd116.751535
CD123	Brillant Violet 510	Human	6H6	BioLegend	306021	1:200	https://www.biolegend.com/en-us/products/brilliant-violet-510-anti-human-cd123-antibody-8524
CD135	PE	Human	4G8	BD Biosciences	558996	1:50	https://www.bdbiosciences.com/en-us/products/reagents/flow-cytometry-reagents/research-reagents/single-color-antibodies-ruo/pe-mouse-anti-human-cd135.558996
CD14	APC-Cy7	Human	M5E2	BioLegend	301819	1:100	https://www.biolegend.com/en-us/products/apc-cyanine7-anti-human-cd14-antibody-3293
CD14	APC-eF750	Human	Tuk4	ThermoFisher Scientific	MHCD1427	1:400	https://www.thermofisher.com/antibody/product/CD14-Antibody-clone-TuK4-Monoclonal/MHCD1427
CD19	AF700	Human	SJ25C1	Invitrogen	56019842	1:50	https://www.thermofisher.com/antibody/product/CD19-Antibody-clone-SJ25C1-Monoclonal/56-0198-42
CD19	BUV395	Human	SJ25-C1	BD Biosciences	563551	1:50	https://www.bdbiosciences.com/en-us/products/reagents/flow-cytometry-reagents/research-reagents/single-color-antibodies-ruo/buv395-mouse-anti-human-cd19.563551
CD271 (LNGFR)	APC	Human	ME20.4-1.H4	MACS	130-113-418	1:200	https://www.miltenyibiotec.com/CH-en/products/cd271-lngfr-antibody-anti-human-me20-4-1-h4.html#conjugate=apc:size=100-tests-in-200-ul
CD271 (LNGFR)	AF700	Human	ME20.4	BioLegend	345117	1:100	https://www.biolegend.com/en-us/products/alexa-fluor-700-anti-human-cd271-ngfr-antibody-16283
CD271 (LNGFR)	FITC	Human	ME20.4-1.H4	MACS	130-113-420	1:200	https://www.miltenyibiotec.com/CH-en/products/cd271-lngfr-antibody-anti-human-me20-4-1-h4.html#conjugate=fitc:size=100-tests-in-200-ul
CD271 (LNGFR)	PE	Human	ME20.4-1.H4	MACS	130-113-421	1:200	https://www.miltenyibiotec.com/CH-en/products/cd271-lngfr-antibody-anti-human-me20-4-1-h4.html#conjugate=pe:size=100-tests-in-200-ul
CD279	Brillant Violet 510	Mouse	29F 1A12	BioLegend	135241	1:100	https://www.biolegend.com/en-gb/search-results/brilliant-violet-510-anti-mouse-cd279-pd-1-antibody-14923?GroupID=BLG7928
CD279	PE	Mouse	RMP1-30	BioLegend	109104	1:100	https://www.biolegend.com/fr-ch/products/pe-anti-mouse-cd279-pd-1-antibody-454?GroupID=BLG4702
CD279	PE-Cy7	Mouse	J43	Invitrogen	25-9985-82	1:100	https://www.thermofisher.com/antibody/product/CD279-PD-1-Antibody-clone-J43-Monoclonal/25-9985-82
CD3	APC	Mouse	17A2	BioLegend	100236	1:100	https://www.biolegend.com/ja-jp/productstab/apc-anti-mouse-cd3-antibody-8055
CD3	Brillant Violet 605	Mouse	17A2	BioLegend	100237	1:100	https://www.biolegend.com/en-us/search-results/brilliant-violet-605-anti-mouse-cd3-antibody-8503?GroupID=BLG6740
CD3	Brillant Violet 650	Mouse	17A2	BioLegend	100229	1:100	https://www.biolegend.com/en-ie/products/brilliant-violet-650-anti-mouse-cd3-antibody-7843?GroupID=BLG242
CD3	FITC	Mouse	17A2	BioLegend	100204	1:100	https://www.biolegend.com/en-us/products/fitc-anti-mouse-cd3-antibody-45?GroupID=BLG6732
CD3	Brillant Violet 605	Human	OKT3	BioLegend	317321	1:100	https://www.biolegend.com/en-us/products/brilliant-violet-605-anti-human-cd3-antibody-7666
CD3	AF700	Human	HIT3a	BioLegend	300323	1:400	https://www.biolegend.com/en-us/products/alexa-fluor-700-anti-human-cd3-antibody-3417
CD3	PE	Human	SK7	BioLegend	344805	1:100	https://www.biolegend.com/en-us/products/pe-anti-human-cd3-antibody-6511
CD3	APC-eF780	Human	SK7	eBioscience	47-0036-41	1:100	https://www.thermofisher.com/antibody/product/CD3-Antibody-clone-SK7-Monoclonal/47-0036-41
CD3	BUV395	Human	UCHT1	BD Biosciences	563548	1:50	https://www.bdbiosciences.com/en-us/products/reagents/flow-cytometry-reagents/research-reagents/single-color-antibodies-ruo/buv395-mouse-anti-human-cd3.563548
CD335	FITC	Human	9E2	BioLegend	331922	1:200	https://www.biolegend.com/en-us/products/fitc-anti-human-cd335-nkp46-antibody-8464
CD335	PE	Human	9E2	BioLegend	331907	1:100	https://www.biolegend.com/en-us/products/pe-anti-human-cd335-nkp46-antibody-4577
CD34	PE-Dazzle594	Human	581	BioLegend	343533	1:100	https://www.biolegend.com/en-us/products/pe-dazzle-594-anti-human-cd34-antibody-10077
CD366	Briliant Violet 711	Mouse	RMT3-23	BioLegend	119727	1:100	https://www.biolegend.com/en-gb/products/brilliant-violet-711-anti-mouse-cd366-tim-3-antibody-14918?GroupID=BLG10656
CD4	AF700	Mouse	RM4-5	BioLegend	100536	1:400	https://www.biolegend.com/en-us/search-results/alexa-fluor-700-anti-mouse-cd4-antibody-3386?GroupID=BLG4211
CD4	Alexa Fluor 647	Mouse	RM4-5	BioLegend	100530	1:100	https://www.biolegend.com/en-gb/products/alexa-fluor-647-anti-mouse-cd4-antibody-2696?GroupID=BLG4745
CD4	AF700	Mouse	GK1.5	BioLegend	100430	1:100	https://www.biolegend.com/en-ie/products/alexa-fluor-700-anti-mouse-cd4-antibody-3385?GroupID=BLG4745
CD4	Brillant Violet 605	Mouse	RM4-5	BioLegend	100548	1:100	https://www.biolegend.com/ja-jp/products/brilliant-violet-605-anti-mouse-cd4-antibody-7627?GroupID=BLG4745
CD4	Brillant Violet 711	Mouse	RM4-5	Biolegend	100550	1:100	https://www.biolegend.com/en-us/products/brilliant-violet-711-anti-mouse-cd4-antibody-7925?GroupID=BLG4211
CD4	PE	Human/Mouse	RM4-5	BioLegend	100512	1:100	https://www.biolegend.com/en-us/products/pe-anti-mouse-cd4-antibody-482
CD44	Alexa Fluor 488	Human/Mouse	IM7	BioLegend	103016	1:100	https://www.biolegend.com/fr-ch/explore-new-products/alexa-fluor-488-anti-mouse-human-cd44-antibody-3097?GroupID=BLG10248
CD44	APC	Human/Mouse	IM7	BioLegend	103012	1:200	https://www.biolegend.com/en-us/products/apc-anti-mouse-human-cd44-antibody-312
CD44	PerCP-Cy 5.5	Mouse	IM7	BioLegend	103032	1:100	https://www.biolegend.com/en-us/search-results/percp-cyanine5-5-anti-mouse-human-cd44-antibody-5605?GroupID=BLG10248
CD45	APC	Mouse	30-F11	eBioscience	17-0451-82	1:100	https://www.thermofisher.com/antibody/product/CD45-Antibody-clone-30-F11-Monoclonal/17-0451-82#:~:text=17%2D0451%2D83%20was%20used,diagnosis%20and%20prognosis%20of%20mesothelioma.
CD45	APC	Mouse	30-F11	BioLegend	103112	1:100	https://www.biolegend.com/en-ie/products/apc-anti-mouse-cd45-antibody-97
CD45	Brillant Violet 510	Mouse	30-F11	BioLegend	103138	1:400	https://www.biolegend.com/fr-lu/cell-health/brilliant-violet-510-anti-mouse-cd45-antibody-7995
CD45	Pacific Blue	Mouse	30-F11	BioLegend	103126	1:100	https://www.biolegend.com/en-gb/search-results/pacific-blue-anti-mouse-cd45-antibody-3102?GroupID=BLG6841
CD45	PE	Mouse	30-F11	BioLegend	103106	1:100	https://www.biolegend.com/en-us/products/pe-anti-mouse-cd45-antibody-100
CD45.1	FITC	Mouse	A20	Invitrogen	11-0453-82	1:100	https://www.thermofisher.com/antibody/product/CD45-1-Antibody-clone-A20-Monoclonal/11-0453-82
CD45.1	PE	Mouse	A20	Invitrogen	12-0453-82	1:100	https://www.thermofisher.com/antibody/product/CD45-1-Antibody-clone-A20-Monoclonal/12-0453-82
CD45.1	PE-Cy7	Mouse	104	BioLegend	110730	1:100	https://www.biolegend.com/fr-fr/products/pe-cyanine7-anti-mouse-cd45-1-antibody-4917?GroupID=BLG1933
CD45.2	APC	Mouse	104	eBioscience	17-0454-82	1:100	https://www.thermofisher.com/antibody/product/CD45-2-Antibody-clone-104-Monoclonal/17-0454-82
CD45.2	APC-Cy7	Mouse	104	BioLegend	109824	1:100	https://www.biolegend.com/nl-nl/products/apc-cyanine7-anti-mouse-cd45-2-antibody-3906
CD45.2	APC-eFluor 780	Mouse	104	eBioscience	47-0454-82	1:100	https://www.thermofisher.com/antibody/product/CD45-2-Antibody-clone-104-Monoclonal/47-0454-82
CD45.2	Pacific Blue	Mouse	104	eBioscience	109820	1:100	https://www.biolegend.com/en-us/products/pacific-blue-anti-mouse-cd45-2-antibody-3108?GroupID=BLG1934
CD45.2	PE	Mouse	104	Invitrogen	12-0454-82	1:100	https://www.thermofisher.com/antibody/product/CD45-2-Antibody-clone-104-Monoclonal/12-0454-82
CD45R	Brillant Violet 605	Mouse	RA3-6B2	BioLegend	103244	1:100	https://www.biolegend.com/en-us/products/brilliant-violet-605-anti-mouse-human-cd45r-b220-antibody-7870?GroupID=GROUP20
CD45RA	BUV737	Human	HI100	BD Biosciences	612846	1:400	https://www.bdbiosciences.com/en-us/products/reagents/flow-cytometry-reagents/research-reagents/single-color-antibodies-ruo/buv737-mouse-anti-human-cd45ra.612846
CD62L	Alexa Fluor 488	Mouse	MEL-14	BioLegend	104420	1:100	https://www.biolegend.com/fr-fr/products/alexa-fluor-488-anti-mouse-cd62l-antibody-3115?GroupID=GROUP20
CD62L	PE-Cy7	Mouse	MEL-14	BioLegend	104418	1:100	https://www.biolegend.com/en-us/products/pe-cyanine7-anti-mouse-cd62l-antibody-1922?GroupID=BLG10670
CD62L	PE/Dazzle	Mouse	MEL-14	BioLegend	104448	1:200	https://www.biolegend.com/de-de/products/pe-dazzle-594-anti-mouse-cd62l-antibody-12137
CD64	Brilliant Violet 711	Mouse	X54-5/7.1	BioLegend	139311	1:100	https://www.biolegend.com/fr-ch/products/brilliant-violet-711-anti-mouse-cd64-fcgammari-antibody-9920?GroupID=BLG8805
CD66b	FITC	Human	G10F5	BioLegend	305103	1:400	https://www.biolegend.com/en-us/products/fitc-anti-human-cd66b-antibody-666
CD66b	PE	Human	G10F5	BioLegend	305106	1:400	https://www.biolegend.com/en-us/products/pe-anti-human-cd66b-antibody-6529
CD86	APC-Cy7	Mouse	GL-1	BioLegend	553030	1:100	https://www.biolegend.com/en-us/products/apc-cyanine7-anti-mouse-cd86-antibody-6554?GroupID=BLG11928
CD86	Pacific Blue	Human	IT2.2	BioLegend	305417	1:100	https://www.biolegend.com/en-us/products/pacific-blue-anti-human-cd86-antibody-3357
CD86	Pacific Blue	Mouse	GL-1	BioLegend	105022	1:100	https://www.biolegend.com/en-us/products/pacific-blue-anti-mouse-cd86-antibody-3122
CD8a	PerCp-Cy5.5	Mouse	53-6.7	BioLegend	100734	1:400	https://www.biolegend.com/nl-be/products/percp-cyanine5-5-anti-mouse-cd8a-antibody-4255
CD8a	Alexa Fluor 700	Mouse	53-6.7	eBioscience	56-0081-82	1:100	https://www.thermofisher.com/antibody/product/CD8a-Antibody-clone-53-6-7-Monoclonal/56-0081-82
CD8a	APC-Cy7	Mouse	53-6.7	BioLegend	100714	1:100	https://www.biolegend.com/en-gb/diagnostic-1/apc-cyanine7-anti-mouse-cd8a-antibody-2269
CD8a	Briliant Violet 605	Mouse	53-6.7	BioLegend	100744	1:100	https://www.biolegend.com/en-us/productstab/brilliant-violet-605-anti-mouse-cd8a-antibody-7636
CD8a	Brillant Violet 510	Mouse	53-6.7	BioLegend	100752	1:100	https://www.biolegend.com/en-ie/cell-health/brilliant-violet-510-anti-mouse-cd8a-antibody-7992
CD8a	FITC	Mouse	53-6.7	Invitrogen	553030	1:100	https://www.bdbiosciences.com/en-ch/products/reagents/flow-cytometry-reagents/research-reagents/single-color-antibodies-ruo/fitc-rat-anti-mouse-cd8a.553030
CD8a	PE	Mouse	53-6.7	Invitrogen	12-0081-82	1:100	https://www.thermofisher.com/antibody/product/CD8a-Antibody-clone-53-6-7-Monoclonal/12-0081-82
CD8a	PerCP-Cy 5.5	Human	SK1	BioLegend	353803	1:400	https://www.biolegend.com/en-us/products/percp-cyanine5-5-anti-human-cd8-antibody-6389
CLEC9A	APC	Human	8F9	BioLegend	353806	1:200	https://www.biolegend.com/en-us/products/apc-anti-human-cd370-clec9a-dngr1-antibody-11714
F4/80	Alexa Fluor 488	Mouse	BM8	BioLegend	123120	1:100	https://www.biolegend.com/en-us/products/alexa-fluor-488-anti-mouse-f4-80-antibody-4073
F4/80	APC	Mouse	BM8	BioLegend	123116	1:100	https://www.biolegend.com/en-us/products/apc-anti-mouse-f4-80-antibody-4071
F4/80	APC-Cy7	Mouse	BM8	BioLegend	123118	1:100	https://www.biolegend.com/en-us/products/apc-cyanine7-anti-mouse-f4-80-antibody-4072
F4/80	PE-Cy7	Mouse	BM8	BioLegend	123114	1:200	https://www.biolegend.com/en-us/productstab/pe-cyanine7-anti-mouse-f4-80-antibody-4070?GroupID=BLG5319
Granzyme B	PE-Texas Red	Human/Mouse	GB11	ThermoFisher Scientific	GRB17	1:100	https://www.thermofisher.com/antibody/product/Granzyme-B-Antibody-clone-GB11-Monoclonal/GRB17
GD2	APC	Human/Mouse	14G2a	BioLegend	357305	1:100	https://www.biolegend.com/fr-ch/products/apc-anti-human-ganglioside-gd2-antibody-9298
HLA-A/B/C	PerCp-Cy5.5	Human	W6/32	BioLegend	311419	1:400	https://www.biolegend.com/en-us/products/percp-cyanine5-5-anti-human-hla-a-b-c-antibody-7057
HLA-A2	PE-Cy7	Human	BB7.2	BioLegend	343313	1:100	https://www.biolegend.com/en-us/products/pe-cyanine7-anti-human-hla-a2-antibody-8184
HLA-DR	PerCp-Cy5.5	Human	L243	BioLegend	307629	1:200	https://www.biolegend.com/en-us/products/percp-cyanine5-5-anti-human-hla-dr-antibody-4246
IFN-γ	APC	Mouse	XMG1.2	BD Biosciences	562018	1:100	https://www.bdbiosciences.com/en-au/products/reagents/flow-cytometry-reagents/research-reagents/single-color-antibodies-ruo/apc-rat-anti-mouse-ifn.562018
IFN-γ	APC	Mouse	XMG1.2	BioLegend	505810	1:200	https://www.biolegend.com/en-us/products/apc-anti-mouse-ifn-gamma-antibody-993?GroupID=GROUP24
IFN-γ	PE-Cy7	Human	4S.B3	BD Biosciences	561036	1:200	https://www.bdbiosciences.com/en-us/products/reagents/flow-cytometry-reagents/research-reagents/single-color-antibodies-ruo/pe-cy-7-mouse-anti-human-ifn.561036
ISO	PE-Cy7	Human	MPC-11	BioLegend	400325	1:50	https://www.biolegend.com/en-us/products/pe-cyanine7-mouse-igg2b-kappa-isotype-ctrl-1928
ISO	PerCp-Cy5.5	Human	MOPC-173	BioLegend	400257	1:800	https://www.biolegend.com/en-us/products/percp-cyanine5-5-mouse-igg2a-kappa-isotype-ctrl-4207
Ly6C	Alexa Fluor 700	Mouse	HK1.4	BioLegend	128024	1:100	https://www.biolegend.com/en-us/products/alexa-fluor-700-anti-mouse-ly-6c-antibody-6757
Ly6C	Briliant Violet 605	Mouse	HK1.4	BioLegend	128035	1:100	https://www.biolegend.com/en-us/products/brilliant-violet-605-anti-mouse-ly-6c-antibody-8727
Ly6C	Briliant Violet 711	Mouse	HK1.4	BioLegend	128037	1:100	https://www.biolegend.com/en-us/products/brilliant-violet-711-anti-mouse-ly-6c-antibody-8935
Ly6C	Brillant Violet 650	Mouse	HK1.4	BioLegend	128049	1:200	https://www.biolegend.com/en-us/products/brilliant-violet-650-anti-mouse-ly-6c-antibody-17378
Ly6G	Brillant Violet 605	Mouse	1A8	BioLegend	127639	1:100	https://www.biolegend.com/en-us/products/brilliant-violet-605-anti-mouse-ly-6g-antibody-12244
Ly6G	Pacific Blue	Mouse	1A8	BioLegend	127611	1:100	https://www.biolegend.com/en-us/products/pacific-blue-anti-mouse-ly-6g-antibody-6082
MHC class II	APC-Cy7	Mouse	M5/114.15.2	BioLegend	107628	1:100	https://www.biolegend.com/en-us/products/apc-cyanine7-anti-mouse-i-a-i-e-antibody-5966
MHC class II	FITC	Mouse	M5/114.15.2	Invitrogen	11-5321-85	1:100	https://www.thermofisher.com/antibody/product/MHC-Class-II-I-A-I-E-Antibody-clone-M5-114-15-2-Monoclonal/11-5321-82
MHC class II	Pacific Blue	Mouse	M5/114.15.2	BioLegend	107620	1:100	https://www.biolegend.com/en-us/products/pacific-blue-anti-mouse-i-a-i-e-antibody-3136?GroupID=BLG11931
MHC class II	PE	Mouse	M5/114.15.2	ThermoFisher Scientific	A14763	1:100	https://www.thermofisher.com/antibody/product/I-A-I-E-Antibody-clone-M5-114-15-2-Monoclonal/A14763
MHC class II	Pe-Cy5	Mouse	M5/114.15.2	BioLegend	107612	1:100	https://www.biolegend.com/de-at/products/pe-cyanine5-anti-mouse-i-a-i-e-antibody-2487?GroupID=BLG11931
MHC class II	PE-Cy7	Mouse	M5/114.15.2	BioLegend	107629	1:100	https://www.biolegend.com/fr-fr/products/pe-cyanine7-anti-mouse-i-a-i-e-antibody-6136
MHC class II	PerCP-Cy 5.5	Mouse	M5/114.15.2	BioLegend	107625	1:200	https://www.biolegend.com/nl-nl/products/percp-cyanine5-5-anti-mouse-i-a-i-e-antibody-4282
NK 1.1	AF488	Mouse	PK136	BioLegend	108706	1:200	https://www.biolegend.com/en-us/products/fitc-anti-mouse-nk-1-1-antibody-429
NK 1.1	Brilliant Violet 711	Mouse	PK136	BioLegend	108745	1:200	https://www.biolegend.com/en-us/products/brilliant-violet-711-anti-mouse-nk-1-1-antibody-9576
NK1.1	PE-Cy7	Mouse	PK136	BioLegend	108714	1:100	https://www.biolegend.com/en-us/soluble-mhc/pe-cyanine7-anti-mouse-nk-1-1-antibody-2840
TCRβ	Brillant Violet 650	Mouse	H57-597	BioLegend	109251	1:400	https://www.biolegend.com/en-us/products/brilliant-violet-650-anti-mouse-tcr-beta-chain-antibody-17652
TIM-3	Brillant Violet 711	Mouse	RMT3-23	BioLegend	119727	1:100	https://www.biolegend.com/en-gb/products/brilliant-violet-711-anti-mouse-cd366-tim-3-antibody-14918?GroupID=BLG10656
TNFa	A700	Mouse	MAb11	BD Biosciences	561023	1:100	https://www.bdbiosciences.com/en-ch/products/reagents/flow-cytometry-reagents/research-reagents/single-color-antibodies-ruo/alexa-fluor-700-mouse-anti-human-tnf.561023
TNFa	PE-Cy7	Mouse	MP6-XT22	BioLegend	506324	1:100	https://www.biolegend.com/de-at/products/pe-cyanine7-anti-mouse-tnf-alpha-antibody-5866?GroupID=GROUP24
TNFa	PE/Dazzle	Human	MAb11	BD Biosciences	561023	1:100	https://www.bdbiosciences.com/en-us/products/reagents/flow-cytometry-reagents/research-reagents/single-color-antibodies-ruo/alexa-fluor-700-mouse-anti-human-tnf.561023
XCR1	Brillant Violet 510	Mouse/Rat	ZET	BioLegend	148218	1:100	https://www.biolegend.com/fr-lu/products/brilliant-violet-510-anti-mouse-rat-xcr1-antibody-10751
XCR1	PE	Mouse	ZET	BioLegend	148204	1:200	https://www.biolegend.com/en-us/clone-search/pe-anti-mouse-rat-xcr1-antibody-10217?GroupID=GROUP20
β2-microglobulin	PE	Mouse	A16041A	BioLegend	154503	1:100	https://www.biolegend.com/fr-ch/products/pe-anti-mouse-beta2-microglobulin-antibody-15126?GroupID=ImportedGROUP1

IMMUNOFLUORESCENCE STAINING

Antigen	Fluorochrome	Reactivity	Clone	Provider	Reference	Dilution used	Validation
F4/80	PE	Mouse	BM8	BioLegend	123109	1:50	https://www.biolegend.com/en-us/products/pe-anti-mouse-f4-80-antibody-4068
CD8a	APC	Mouse	53-6.7	BD Bioscience	561093	1:50	https://www.bdbiosciences.com/en-us/products/reagents/flow-cytometry-reagents/research-reagents/single-color-antibodies-ruo/apc-rat-anti-mouse-cd8a.561093
CD8b	APC	Mouse	YTS156.7.7	BioLegend	126613	1:50	https://www.biolegend.com/en-us/products/apc-anti-mouse-cd8b-antibody-9055
CD3e	PE	Mouse	OKT3	BioLegend	317308	1:100	https://www.biolegend.com/en-us/products/pe-anti-human-cd3-antibody-3645
CD3	PE	Mouse	500A2	BD Bioscience	553240	1:50	https://www.bdbiosciences.com/en-us/products/reagents/flow-cytometry-reagents/research-reagents/single-color-antibodies-ruo/pe-hamster-anti-mouse-cd3e.553240
NKp46	PE	Mouse	29A1.4	BioLegend	137603	1:50	https://www.biolegend.com/en-us/products/pe-anti-mouse-cd335-nkp46-antibody-6523
GFP	Unconj.	GFP	polyclonal	Proteintech	50430-2-AP	1:200	https://www.ptglab.com/products/eGFP-Antibody-50430-2-AP.htm
CD31	Unconj.	Mouse	polyclonal	LifeSpan Biosciences	LS-C150165	1:300	https://www.ncbi.nlm.nih.gov/pmc/articles/PMC6525097/
Secondary (for GFP)	AF488	Rabbit	polyclonal	ThermoFisher	A-21206	1:500	https://www.thermofisher.com/antibody/product/Donkey-anti-Rabbit-IgG-H-L-Highly-Cross-Adsorbed-Secondary-Antibody-Polyclonal/A-21206
Secondary (for CD31)	AF488	Goat	polyclonal	ThermoFisher	A-11055	1:500	https://www.thermofisher.com/antibody/product/Donkey-anti-Goat-IgG-H-L-Cross-Adsorbed-Secondary-Antibody-Polyclonal/A-11055
CD8a	Unconj.	Mouse	H2(20)	Cell signaling	98941S	1:400	https://www.cellsignal.com/products/primary-antibodies/cd8a-d4w2z-xp-rabbit-mab/98941?_requestid=429297&gclid=CjwKCAjwjaWoBhAmEiwAXz8DBaAmzYX7MryLyEhP4yt8IldLyCsvig3c8OP2gyoJ_lJs0ysTRg0TXBoC6g0QAvD_BwE&gclsrc=aw.ds&_requestid=2408425
CD4	Unconj.	Mouse	14-9766	eBioscience	14-9766	1:1000	https://www.thermofisher.com/antibody/product/CD4-Antibody-clone-4SM95-Monoclonal/14-9766-82
Secondary (for CD8a)	HRP*	Rabbit	polyclonal	Agilent DAKO	P044801-2	1:300	https://www.agilent.com/en/product/specific-proteins/elisa-kits-accessories/goat-anti-rabbit-immunoglobulins-hrp-affinity-isolated-2717113
Secondary (for CD4)	HRP**	Rat	polyclonal	Jackson Immuno Research	312-005-045	1:1000	https://www.jacksonimmuno.com/catalog/products/312-005-045

* HRP signal was revealed by Opal 620 reagent pack (FP1495001KT). https://my.akoyabio.com/ccrz__ProductDetails?sku=FP1495001KT&cclcl=en_US

** HRP signal was revealed by Opal 540 reagent pack (FP1494001KT). https://my.akoyabio.com/ccrz__ProductDetails?sku=FP1494001KT&cclcl=en_US

Supplementary Table 1: Antibodies used for flow cytometry and immunofluorescence stainig.
