# Supplementary material for: Cytokine-armed dendritic cell progenitors for antigen-agnostic cancer immunotherapy
Source: Nat Cancer. 2023 Nov 23;5(2):240–61. doi: 10.1038/s43018-023-00668-y (PMC10899110; doi:10.1038/s43018-023-00668-y)
Supplement: Supplementary file 2 — Reporting Summary [file 43018_2023_668_MOESM2_ESM.pdf]

nature portfolio

Corresponding author(s): Michele De Palma

Last updated by author(s): 10/10//23

Reporting Summary

Nature Portfolio wishes to improve the reproducibility of the work that we publish. This form provides structure for consistency and transparency in reporting. For further information on Nature Portfolio policies, see our Editorial Policies and the Editorial Policy Checklist.

Statistics

For all statistical analyses, confirm that the following items are present in the figure legend, table legend, main text, or Methods section.

n/a	Confirmed	
☐	☒	The exact sample size (*n*) for each experimental group/condition, given as a discrete number and unit of measurement
☐	☒	A statement on whether measurements were taken from distinct samples or whether the same sample was measured repeatedly
☐	☒	The statistical test(s) used AND whether they are one- or two-sided<br>*Only common tests should be described solely by name; describe more complex techniques in the Methods section.*
☐	☒	A description of all covariates tested
☐	☒	A description of any assumptions or corrections, such as tests of normality and adjustment for multiple comparisons
☐	☒	A full description of the statistical parameters including central tendency (e.g. means) or other basic estimates (e.g. regression coefficient) AND variation (e.g. standard deviation) or associated estimates of uncertainty (e.g. confidence intervals)
☐	☒	For null hypothesis testing, the test statistic (e.g. *F*, *t*, *r*) with confidence intervals, effect sizes, degrees of freedom and *P* value noted<br>*Give P values as exact values whenever suitable.*
☒	☐	For Bayesian analysis, information on the choice of priors and Markov chain Monte Carlo settings
☒	☐	For hierarchical and complex designs, identification of the appropriate level for tests and full reporting of outcomes
☒	☐	Estimates of effect sizes (e.g. Cohen's *d*, Pearson's *r*), indicating how they were calculated

Our web collection on statistics for biologists contains articles on many of the points above.

Software and code

Policy information about availability of computer code

Data collection

Flow cytometry data were acquired using BD LSRFortessa™ (Becton Dickinson); BD® LSR II SORP (Becton Dickinson); and Attune™ NxT (Invitrogen).

Acquisition of immunofluorescence staining images from tissue sections used a Axioscan microscope (Zeiss).

Luciferase radiance from liver tumors and intracranial gliomas was acquired using an IVIS apparatus (PerkinElmer).

Single cell RNA sequencing used an Illumina HiSeq 4000 paired-end Flow Cell instrument (Illumina).

TCR sequencing used an Illumina MiSeq instrument (Illumina).

Data analysis

Graphs were generated and statistical analyses performed with Prism (GraphPad Software). Error bars indicate the standard error of the mean (SEM), unless indicated otherwise. The number of biological (non-technical) replicates and applied statistical analysis are indicated in the figure legends. Briefly, comparison between two unpaired groups was performed by non-parametric Mann-Whitney test. For multiple comparisons involving one variable, one-way ANOVA was performed followed by Tukey's multiple comparison test, unless otherwise stated in the figure legends. Simultaneous analysis of two variables (tumor growth over time) among multiple groups was performed by two-way ANOVA followed by Tukey's (three groups or more) or Sidak's (two groups) multiple comparison test. Other statistical tests were applied in selected cases, as detailed in the figure legends.

Flow cytometry analysis used FlowJo, LLC V10.1. Gating strategies are reported in the section "Flow Cytometry" below.

To analyze immunofluorescent images, we used the Qupath 77 software. A region of interest (ROI) of the tumor was defined by setting a DAPI threshold and adjusted manually to include DAPI-negative necrotic areas and exclude adjacent tissue. For quantification of CD31+ and F4/80+ area, a CD31+ or F4/80+ pixel classifier trained on 10% of the dataset was applied. The area of each marker was normalized to the total tumor area. For quantification of CD3+, CD3+CD8+, CD4+, and NKp46+ cells, nuclei segmentation was performed using StarDist and "dsb2018_heavy_augment.pb" model on the DAPI channel. A CD3+, CD8+, CD4+, or NKp46+ cell composite classifier trained on 15% of the dataset was applied.

IVIS imaging used IVIS software (v4.7.3; PerkinElmer)

RNA-seq analysis used CellRanger (v 7.0) and Seurat (4.1.1) to map reads on mouse genome in single cell RNA sequencing datasets, and ClusterProfiler (4.4.1) for gene pathway analysis.

TCR analysis was performed by using the Immunarch library in R (4.2.1).

For manuscripts utilizing custom algorithms or software that are central to the research but not yet described in published literature, software must be made available to editors and reviewers. We strongly encourage code deposition in a community repository (e.g. GitHub). See the Nature Portfolio guidelines for submitting code & software for further information.

Data

Policy information about availability of data

All manuscripts must include a data availability statement. This statement should provide the following information, where applicable:

- Accession codes, unique identifiers, or web links for publicly available datasets
- A description of any restrictions on data availability
- For clinical datasets or third party data, please ensure that the statement adheres to our policy

All raw data supporting the findings of this study are presented as Source Data.

ScRNA-seq data have been deposited in GEO with GEO accession GSE228014.

TCR-Seq data have been deposited in GEO with GEO accession GSE228161.

Human research participants

Policy information about studies involving human research participants and Sex and Gender in Research.

Reporting on sex and gender	Information on sex and gender was not considered in the study design and analysis.
Population characteristics	Population characteristics were not considered in the study design and analysis.
Recruitment	CD34+ cells from cord blood (CB) and mobilized peripheral blood (MPB) were purchased from Stem Cell Technologies (CB) and Lonza (CB and MPB). Peripheral blood from healthy donors was obtained from the Blood Transfusion Center (Lausanne, Switzerland) under protocol P_297.
Ethics oversight	The use of primary blood cells did not require approval from an Ethics Committee.

Note that full information on the approval of the study protocol must also be provided in the manuscript.

Field-specific reporting

Please select the one below that is the best fit for your research. If you are not sure, read the appropriate sections before making your selection.

☒ Life sciences ☐ Behavioural & social sciences ☐ Ecological, evolutionary & environmental sciences

For a reference copy of the document with all sections, see nature.com/documents/nr-reporting-summary-flat.pdf

Life sciences study design

All studies must disclose on these points even when the disclosure is negative.

Sample size

Studies involving independent cohorts of mice were typically performed once, with the several exceptions stated in the figure legends. Each study was designed to use the minimum number of mice required to obtain informative results (i.e., quantitative data suited to statistical analysis) and sufficient material for further studies. No specific statistical tests were used to predetermine the sample size; our previous experience with the different tumor models provided guidance about the adequate number of mice that would provide statistically significant results. Based on these considerations, we typically employed experimental cohorts of 5-10 mice.

Experiments involving human primary cells (Fig. 8) used several independent donors (5 for MPB; 7 for CB) for initial experiments aimed to characterize the key properties of the DCPs (expansion and yield: Fig. 8C-D). For these studies, we employed relatively high numbers of

	donors (5-7) to verify reproducibility of the key results across independent donors. Additional studies shown in Fig. 8F-I were aimed to evaluate qualitative differences between moDCs and DCPs and, therefore, only used 1-3 independent donors; statistical analyses were not performed on these samples.
Data exclusions	Outliers were never excluded from analysis. In rare cases, selected samples were lost and excluded from specific analyses because of technical flaws during sample collection, processing or data acquisition. Exclusion criteria were not pre-established.
Replication	Biological replicates in each experiment are defined in the figure legends. Studies involving mice were typically performed once to limit the use of animals, with the exception of control groups or standard treatments (e.g., DCP treatment), which were replicated in independent experiments. Cell culture experiments (Fig. 8) were performed 5-7 times for obtaining the key results and 1-3 times for additional qualitative data.
Randomization	Tumor-bearing mice were randomized before treatment by allocating mice bearing tumors ranked by volume to alternate treatment groups. Certain mice were excluded at the time of randomization, for example, if the tumors were not detectable or were significantly larger than the average tumor volume. We further verified that the mean tumor volume before treatment was comparable in the different experimental groups, when applicable.
Blinding	Blinding in mouse studies was not possible due to tagging of the mice and access to identification codes by the investigators. Although data acquisition was blinded in most cases (samples were labeled with identification codes that do not indicate the treatment/experimental group), the investigators had access to the identity of the samples through the identification codes. In most applications, acquisition of the data was performed by expert technical staff with knowledge of the identity of the experimental groups but without knowledge of the expected treatment effects. The investigators were blinded when collecting most data (tumor growth; acquisition and fofware-based quantification of immunofluorescence staining datasets; acquisition of flow cytometry data), but were not blinded when analyzing flow cytometry data (owing to manual processing of the data and access to identification codes by the investigators).

Reporting for specific materials, systems and methods

We require information from authors about some types of materials, experimental systems and methods used in many studies. Here, indicate whether each material, system or method listed is relevant to your study. If you are not sure if a list item applies to your research, read the appropriate section before selecting a response.

Materials & experimental systems

n/a	Involved in the study	
☐	☒	Antibodies
☐	☒	Eukaryotic cell lines
☒	☐	Palaeontology and archaeology
☐	☒	Animals and other organisms
☒	☐	Clinical data
☒	☐	Dual use research of concern

Methods

n/a	Involved in the study	
☒	☐	ChIP-seq
☐	☒	Flow cytometry
☒	☐	MRI-based neuroimaging

Antibodies

Antibodies used

A list of antibodies is provided. We indicate the following: Antigen; Fluorochrome; Reactivity; Clone; Provider; Reference (catalogue #); Dilution.

FLOW CYTOMETRY

Antigen - Fluorochrome - Reactivity - Clone - Provider - Reference - Dilution
BDCA2/CD303 Brillant Violet 605 Human 201A BioLegend 354223 1:100
BDCA3/CD141 PE-Cy7 Human M80 BioLegend 344109 1:400
BDCA1/CD1c Brillant Violet 785 Human L161 BioLegend 331543 1:100
CD10 FITC Human Hl10a BioLegend 982204 1:100
CD103 Brillant Violet 605 Mouse 2E7 BioLegend 121433 1:100
CD103 Brillant Violet 711 Mouse 2E7 BioLegend 121435 1:100
CD103 FITC Mouse 2E7 BioLegend 121420 1:200
CD103 PE Mouse 2E7 BioLegend 121406 1:100
CD115 APC Mouse AFS98 ThermoFisher Scientific 17-1152-80 1:100
CD115 APC Human 9-4D2-1E4 BioLegend 347305 1:50
CD117 Brillant Violet 421 Human 104D2 BioLegend 313215 1:50
CD11b Briliant Violet 510 Mouse M1/70 BioLegend 101263 1:100
CD11b Brillant Violet 650 Human/Mouse M1/70 BioLegend 101239 1:400
CD11b Brillant Violet 711 Human/Mouse M1/70 BioLegend 101241 1:200
CD11b PE-Cy7 Human M1/70 eBioscience 25-0112-82 1:400
CD11c APC Mouse N418 BioLegend 117309 1:200
CD11c APC-Cy7 Mouse N418 BioLegend 117324 1:100
CD11c Brillant Violet 421 Mouse N418 BioLegend 117330 1:100
CD11c Brillant Violet 650 Mouse N418 BioLegend 117339 1:100
CD11c Brillant Violet 650 Human 3.9 BioLegend 301637 1:50

CD11c PE-Dazzle594 Human 3.9 BioLegend 301641 1:100
CD116 BUV615 Human hGMCSFR-M1 BD Biosciences 751535 1:200
CD123 Briliant Violet 510 Human 6H6 BioLegend 306021 1:200
CD135 PE Human 4G8 BD Biosciences 558996 1:50
CD14 APC-Cy7 Human M5E2 BioLegend 301819 1:100
CD14 APC-eF750 Human Tuk4 ThermoFisher Scientific MHCD1427 1:400
CD19 AF700 Human SJ25C1 Invitrogen " 56019842" 1:50
CD19 BUV395 Human SJ25-C1 BD Biosciences 563551 1:50
CD271/LNGFR APC Human ME20.4-1.H4 MACS 130-113-418 1:200
CD271/LNGFR AF700 Human ME20.4 BioLegend 345117 1:100
CD271/LNGFR FITC Human ME20.4-1.H4 MACS 130-113-420 1:200
CD279 Brillant Violet 510 Mouse 29F 1A12 BioLegend 135241 1:100
CD279 PE Mouse RMP1-30 BioLegend 109104 1:100
CD279 PE-Cy7 Mouse J43 Invitrogen 25-9985-82 1:100
CD3 APC Mouse 17A2 BioLegend 100236 1:100
CD3 Briliant Violet 605 Mouse 17A2 BioLegend 100237 1:100
CD3 Brillant Violet 650 Mouse 17A2 BioLegend 100229 1:100
CD3 FITC Mouse 17A2 BioLegend 100204 1:100
CD3 Briliant Violet 605 Human OKT3 BioLegend 317321 1:100
CD3 AF700 Human HIT3a BioLegend 300323 1:400
CD3 PE Human SK7 BioLegend 344805 1:100
CD3 APC-eF780 Human SK7 eBioscience 47-0036-41 1:100
CD3 BUV395 Human UCHT1 BD Biosciences 563548 1:50
CD335 FITC Human 9E2 BioLegend 331922 1:200
CD335 PE Human 9E2 BioLegend 331907 1:100
CD34 PE-Dazzle594 Human 581 BioLegend 343533 1:100
CD366 Briliant Violet 711 Mouse RMT3-23 BioLegend 119727 1:100
CD4 AF700 Mouse RM4-5 BioLegend 100536 1:400
CD4 Alexa Fluor 647 Mouse RM4-5 BioLegend 100530 1:100
CD4 AF700 Mouse GK1.5 BioLegend 100430 1:100
CD4 Brillant Violet 605 Mouse RM4-5 BioLegend 100548 1:100
CD4 Brillant Violet 711 Mouse RM4-5 Biolegend 100550 1:100
CD4 PE Human/Mouse RM4-5 BioLegend 100512 1:100
CD44 Alexa Fluor 488 Human/Mouse IM7 BioLegend 103016 1:100
CD44 APC Human/Mouse IM7 BioLegend 103012 1:200
CD44 PerCP-Cy 5.5 Mouse IM7 BioLegend 103032 1:100
CD45 APC Mouse 30-F11 eBioscience 17-0451-82 1:100
CD45 APC Mouse 30-F11 BioLegend 103112 1:100
CD45 Brillant Violet 510 Mouse 30-F11 BioLegend 103138 1:400
CD45 Pacific Blue Mouse 30-F11 BioLegend 103126 1:100
CD45 PE Mouse 30-F11 BioLegend 103106 1:100
CD45.1 FITC Mouse A20 Invitrogen 11-0453-82 1:100
CD45.1 PE Mouse A20 Invitrogen 12-0453-82 1:100
CD45.1 PE-Cy7 Mouse 104 BioLegend 110730 1:100
CD45.2 APC Mouse 104 eBioscience 17-0454-82 1:100
CD45.2 APC-Cy7 Mouse 104 BioLegend 109824 1:100
CD45.2 " APC-eFluor 780" Mouse 104 eBioscience 47-0454-82 1:100
CD45.2 Pacific Blue Mouse 104 eBioscience 109820 1:100
CD45.2 PE Mouse 104 Invitrogen 12-0454-82 1:100
CD45R Brillant Violet 605 Mouse RA3-6B2 BioLegend 103244 1:100
CD45RA BUV737 Human HI100 BD Biosciences 612846 1:400
CD62L Alexa Fluor 488 Mouse MEL-14 BioLegend " 104420" 1:100
CD62L PE-Cy7 Mouse MEL-14 BioLegend 104418 1:100
CD62L PE/Dazzle Mouse MEL-14 BioLegend 104448 1:200
CD64 Brilliant Violet 711 Mouse X54-5/7.1 BioLegend 139311 1:100
CD66b FITC Human G10F5 BioLegend 305103 1:400
CD66b PE Human G10F5 BioLegend 305106 1:400
CD86 APC-Cy7 Mouse GL-1 BioLegend 553030 1:100
CD86 Pacific Blue Human IT2.2 BioLegend 305417 1:100
CD86 Pacific Blue Mouse GL-1 BioLegend 105022 1:100
CD8a PerCp-Cy5.5 Mouse 53-6.7 BioLegend 100734 1:400
CD8a Alexa Fluor 700 Mouse 53-6.7 eBioscience 56-0081-82 1:100
CD8a APC-Cy7 Mouse 53-6.7 BioLegend 100714 1:100
CD8a Briliant Violet 605 Mouse 53-6.7 BioLegend 100744 1:100
CD8a Brillant Violet 510 Mouse 53-6.7 BioLegend 100752 1:100
CD8a FITC Mouse 53-6.7 Invitrogen 553030 1:100
CD8a PE Mouse 53-6.7 Invitrogen 12-0081-82 1:100
CD8a PerCP-Cy 5.5 Human SK1 BioLegend 353803 1:400
CLEC9A APC Human 8F9 BioLegend 353806 1:200
F4/80 Alexa Fluor 488 Mouse BM8 BioLegend 123120 1:100
F4/80 APC Mouse BM8 BioLegend 123116 1:100
F4/80 APC-Cy7 Mouse BM8 BioLegend 123118 1:100
F4/80 PE-Cy7 Mouse BM8 BioLegend 123114 1:200
Granzyme B PE-Texas Red Human/Mouse GB11 ThermoFisher Scientific GRB17 1:100
GD2 APC Mouse/Human 14G2A BioLegend 357305 1:100
HLA-A/B/C PerCp-Cy5.5 Human W6/32 BioLegend 311419 1:400

HLA-A2 PE-Cy7 Human BB7.2 BioLegend 343313 1:100
HLA-DR PerCp-Cy5.5 Human L243 BioLegend 307629 1:200
IFN-γ APC Mouse XMG1.2 BD Biosciences 562018 1:100
IFN-γ APC Mouse XMG1.2 BioLegend 505810 1:200
IFN-γ PE-Cy7 Human 4S.B3 BD Biosciences 561036 1:200
ISO PE-Cy7 Human MPC-11 BioLegend 400325 1:50
ISO PerCp-Cy5.5 Human MOPC-173 BioLegend 400257 1:800
Ly6C Alexa Fluor 700 Mouse HK1.4 BioLegend 128024 1:100
Ly6C Briliant Violet 605 Mouse HK1.4 BioLegend 128035 1:100
Ly6C Briliant Violet 711 Mouse HK1.4 BioLegend 128037 1:100
Ly6C Brillant Violet 650 Mouse HK1.4 BioLegend 128049 1:200
Ly6G Briliant Violet 605 Mouse 1A8 BioLegend " 127639" 1:100
Ly6G Pacific Blue Mouse 1A8 BioLegend 127611 1:100
MHC class II APC-Cy7 Mouse M5/114.15.2 BioLegend 107628 1:100
MHC class II FITC Mouse M5/114.15.2 Invitrogen 11-5321-85 1:100
MHC class II Pacific Blue Mouse M5/114.15.2 BioLegend 107620 1:100
MHC class II PE Mouse M5/114.15.2 ThermoFisher Scientific A14763 1:100
MHC class II Pe-Cy5 Mouse M5/114.15.2 BioLegend 107612 1:100
MHC class II PE-Cy7 Mouse M5/114.15.2 BioLegend 107629 1:100
MHC class II PerCP-Cy 5.5 Mouse M5/114.15.2 BioLegend 107625 1:200
NK 1.1 AF488 Mouse PK136 BioLegend 108706 1:200
NK 1.1 Brillant Violet 711 Mouse PK136 BioLegend 108745 1:200
NK1.1 PE-Cy7 Mouse PK136 BioLegend 108714 1:100
TCRβ Brillant Violet 650 Mouse H57-597 BioLegend 109251 1:400
TIM-3 Briliant Violet 711 Mouse RMT3-23 BioLegend 119727 1:100
TNFa A700 Mouse MAb11 BD Biosciences 561023 1:100
TNFa PE-Cy7 Mouse MP6-XT22 BioLegend 506324 1:100
TNFa PE/Dazzle Human MAb11 BD Biosciences 561023 1:100
XCR1 Brillant Violet 510 Mouse/Rat ZET BioLegend 148218 1:100
XCR1 PE Mouse ZET BioLegend 148204 1:200
β2-microglobulin PE Mouse A16041A BioLegend 154503 1:100

IMMUNOFLUORESCENCE STAINING

Antigen - (Fluorochrome) - Reactivity - Clone - Provider - Reference - Dilution
F4/80 PE Mouse BM8 BioLegend 123109 1:50
CD8a APC Mouse 53-6.7 BD Bioscience 561093 1:50
CD8b APC Mouse YTS156.7.7 BioLegend 126613 1:50
CD3e PE Mouse OKT3 BioLegend 317308 1:100
CD3 PE Mouse 500A2 BD Bioscience 553240 1:50
NKp46 PE Mouse 29A1.4 BioLegend 137603 1:50

GFP Unconj. GFP polyclonal Proteintech 50430-2-AP 1:200
CD31 Unconj. Mouse polyclonal LifeSpan Biosciences LS-C150165 1:300
Secondary (for GFP) AF488 Rabbit polyclonal ThermoFisher A-21206 1:500
Secondary (for CD31) AF488 Goat polyclonal ThermoFisher A-11055 1:500

CD8a Unconj. Mouse H2(20) Cell signaling 98941S 1:400
CD4 Unconj. Mouse 14-9766 eBioscience 14-9766 1:1000
Secondary (for CD8a) HRP* Rabbit polyclonal Agilent DAKO P044801-2 1:300
Secondary (for CD4) HRP** Rat polyclonal Jackson Immuno Research 312-005-045 1:1000

Validation

Only commercially available and routinely used antibodies have been used in this study. All antibodies were validated by the provider, as indicated in the references provided by the manufacturer.

BDCA2/CD303 Brillant Violet 605 Human 201A BioLegend 354223 1:100 https://www.biolegend.com/en-us/products/brilliant-violet-605-anti-human-cd303-bdca-2-antibody-13062

BDCA3/CD141 PE-Cy7 Human M80 BioLegend 344109 1:400 https://www.biolegend.com/en-us/products/pe-cyanine7-anti-human-cd141-thrombomodulin-antibody-8221

BBDCA1/CD1c Brillant Violet 785 Human L161 BioLegend 331543 1:100 https://www.biolegend.com/en-us/products/brilliant-violet-785-anti-human-cd1c-antibody-17375

CD10 FITC Human Hl10a BioLegend 982204 1:100 https://www.biolegend.com/en-us/products/fitc-anti-human-cd10-antibody-14046

CD103 Brillant Violet 605 Mouse 2E7 BioLegend 121433 1:100 https://www.biolegend.com/ja-jp/products/brilliant-violet-605-anti-mouse-cd103-antibody-13558

CD103 Brillant Violet 711 Mouse 2E7 BioLegend 121435 1:100 https://www.biolegend.com/en-us/products/brilliant-violet-711-anti-mouse-cd103-antibody-14411?GroupID=BLG4646

CD103 FITC Mouse 2E7 BioLegend 121420 1:200 https://www.biolegend.com/en-ie/products/fitc-anti-mouse-cd103-antibody-7053?GroupID=GROUP20

CD103 PE Mouse 2E7 BioLegend 121406 1:100 https://www.biolegend.com/en-us/products/pe-anti-mouse-cd103-antibody-3574?GroupID=GROUP20

CD115 APC Mouse AFS98 ThermoFisher Scientific 17-1152-80 1:100 https://www.thermofisher.com/antibody/product/CD115-c-fms-Antibody-clone-AFS98-Monoclonal/17-1152-82

CD115 APC Human 9-4D2-1E4 BioLegend 347305 1:50 https://www.biolegend.com/en-us/products/apc-anti-human-cd115-csf-1r-antibody-8783

CD117 Brillant Violet 421 Human 104D2 BioLegend 313215 1:50 https://www.biolegend.com/en-us/products/brilliant-violet-421-anti-human-cd117-c-kit-antibody-7348

CD11b Briliant Violet 510 Mouse M1/70 BioLegend 101263 1:100 https://www.biolegend.com/en-us/products/brilliant-violet-510-anti-mouse-human-cd11b-antibody-7993

CD11b Brillant Violet 650 Human/Mouse M1/70 BioLegend 101239 1:400 https://www.biolegend.com/en-us/products/brilliant-violet-650-anti-mouse-human-cd11b-antibody-7638

CD11b Brillant Violet 711 Human/Mouse M1/70 BioLegend 101241 1:200 https://www.biolegend.com/en-us/products/brilliant-violet-711-anti-mouse-human-cd11b-antibody-7927

CD11b PE-Cy7 Human M1/70 eBioscience 25-0112-82 1:400 https://www.thermofisher.com/antibody/product/CD11b-Antibody-clone-M1-70-Monoclonal/25-0112-82

CD11c APC Mouse N418 BioLegend 117309 1:200 https://www.biolegend.com/de-at/products/apc-anti-mouse-cd11c-antibody-1813

CD11c APC-Cy7 Mouse N418 BioLegend 117324 1:100 https://www.biolegend.com/fr-ch/products/apc-cyanine7-anti-mouse-cd11c-antibody-3931?GroupID=BLG11937

CD11c Brillant Violet 421 Mouse N418 BioLegend 117330 1:100 https://www.biolegend.com/fr-ch/products/brilliant-violet-421-anti-mouse-cd11c-antibody-7149?GroupID=BLG11937

CD11c Brillant Violet 650 Mouse N418 BioLegend 117339 1:100 https://www.biolegend.com/en-us/products/brilliant-violet-650-anti-mouse-cd11c-antibody-8840?GroupID=BLG11937

CD11c Brillant Violet 650 Human 3.9 BioLegend 301637 1:50 https://www.biolegend.com/en-us/products/brilliant-violet-650-anti-human-cd11c-antibody-8642

CD11c PE-Dazzle594 Human 3.9 BioLegend 301641 1:100 https://www.biolegend.com/en-us/products/pe-dazzle-594-anti-human-cd11c-antibody-10234

CD116 BUV615 Human hGMCSFR-M1 BD Biosciences 751535 1:200 https://www.bdbiosciences.com/en-us/products/reagents/flow-cytometry-reagents/research-reagents/single-color-antibodies-ruo/buv615-mouse-anti-human-cd116.751535

CD123 Briliant Violet 510 Human 6H6 BioLegend 306021 1:200 https://www.biolegend.com/en-us/products/brilliant-violet-510-anti-human-cd123-antibody-8524

CD135 PE Human 4G8 BD Biosciences 558996 1:50 https://www.bdbiosciences.com/en-us/products/reagents/flow-cytometry-reagents/research-reagents/single-color-antibodies-ruo/pe-mouse-anti-human-cd135.558996

CD14 APC-Cy7 Human M5E2 BioLegend 301819 1:100 https://www.biolegend.com/en-us/products/apc-cyanine7-anti-human-cd14-antibody-3293

CD14 APC-eF750 Human Tuk4 ThermoFisher Scientific MHCD1427 1:400 https://www.thermofisher.com/antibody/product/CD14-Antibody-clone-TuK4-Monoclonal/MHCD1427

CD19 AF700 Human SJ25C1 Invitrogen 56019842 1:50 https://www.thermofisher.com/antibody/product/CD19-Antibody-clone-SJ25C1-Monoclonal/56-0198-42

CD19 BUV395 Human SJ25-C1 BD Biosciences 563551 1:50 https://www.bdbiosciences.com/en-us/products/reagents/flow-cytometry-reagents/research-reagents/single-color-antibodies-ruo/buv395-mouse-anti-human-cd19.563551

CD271/dLNGFR APC Human ME20.4-1.H4 MACS 130-113-418 1:200 https://www.miltenyibiotec.com/CH-en/products/cd271-lngfr-antibody-anti-human-me20-4-1-h4.html#conjugate=apc:size=100-tests-in-200-ul

CD271/dLNGFR AF700 Human ME20.4 BioLegend 345117 1:100 https://www.biolegend.com/en-us/products/alexa-fluor-700-anti-human-cd271-ngfr-antibody-16283

CD271/dLNGFR FITC Human ME20.4-1.H4 MACS 130-113-420 1:200 https://www.miltenyibiotec.com/CH-en/products/cd271-lngfr-antibody-anti-human-me20-4-1-h4.html#conjugate=fitc:size=100-tests-in-200-ul

CD279 Brillant Violet 510 Mouse 29F 1A12 BioLegend 135241 1:100 https://www.biolegend.com/en-gb/search-results/brilliant-violet-510-anti-mouse-cd279-pd-1-antibody-14923?GroupID=BLG7928

CD279 PE Mouse RMP1-30 BioLegend 109104 1:100 https://www.biolegend.com/fr-ch/products/pe-anti-mouse-cd279-pd-1-

antibody-454?GroupID=BLG4702

CD279 PE-Cy7 Mouse J43 Invitrogen 25-9985-82 1:100 https://www.thermofisher.com/antibody/product/CD279-PD-1-Antibody-clone-J43-Monoclonal/25-9985-82

CD3 APC Mouse 17A2 BioLegend 100236 1:100 https://www.biolegend.com/ja-jp/productstab/apc-anti-mouse-cd3-antibody-8055

CD3 Briliant Violet 605 Mouse 17A2 BioLegend 100237 1:100 https://www.biolegend.com/en-us/search-results/brilliant-violet-605-anti-mouse-cd3-antibody-8503?GroupID=BLG6740

CD3 Brillant Violet 650 Mouse 17A2 BioLegend 100229 1:100 https://www.biolegend.com/en-ie/products/brilliant-violet-650-anti-mouse-cd3-antibody-7843?GroupID=BLG242

CD3 FITC Mouse 17A2 BioLegend 100204 1:100 https://www.biolegend.com/en-us/products/fitc-anti-mouse-cd3-antibody-45?GroupID=BLG6732

CD3 Briliant Violet 605 Human OKT3 BioLegend 317321 1:100 https://www.biolegend.com/en-us/products/brilliant-violet-605-anti-human-cd3-antibody-7666

CD3 AF700 Human HIT3a BioLegend 300323 1:400 https://www.biolegend.com/en-us/products/alexa-fluor-700-anti-human-cd3-antibody-3417

CD3 PE Human SK7 BioLegend 344805 1:100 https://www.biolegend.com/en-us/products/pe-anti-human-cd3-antibody-6511

CD3 APC-eF780 Human SK7 eBioscience 47-0036-41 1:100 https://www.thermofisher.com/antibody/product/CD3-Antibody-clone-SK7-Monoclonal/47-0036-41

CD3 BUV395 Human UCHT1 BD Biosciences 563548 1:50 https://www.bdbiosciences.com/en-us/products/reagents/flow-cytometry-reagents/research-reagents/single-color-antibodies-ruo/buv395-mouse-anti-human-cd3.563548

CD335 FITC Human 9E2 BioLegend 331922 1:200 https://www.biolegend.com/en-us/products/fitc-anti-human-cd335-nkp46-antibody-8464

CD335 PE Human 9E2 BioLegend 331907 1:100 https://www.biolegend.com/en-us/products/pe-anti-human-cd335-nkp46-antibody-4577

CD34 PE-Dazzle594 Human 581 BioLegend 343533 1:100 https://www.biolegend.com/en-us/products/pe-dazzle-594-anti-human-cd34-antibody-10077

CD366 Briliant Violet 711 Mouse RMT3-23 BioLegend 119727 1:100 https://www.biolegend.com/en-gb/products/brilliant-violet-711-anti-mouse-cd366-tim-3-antibody-14918?GroupID=BLG10656

CD4 AF700 Mouse RM4-5 BioLegend 100536 1:400 https://www.biolegend.com/en-us/search-results/alexa-fluor-700-anti-mouse-cd4-antibody-3386?GroupID=BLG4211

CD4 Alexa Fluor 647 Mouse RM4-5 BioLegend 100530 1:100 https://www.biolegend.com/en-gb/products/alexa-fluor-647-anti-mouse-cd4-antibody-2696?GroupID=BLG4745

CD4 AF700 Mouse GK1.5 BioLegend 100430 1:100 https://www.biolegend.com/en-ie/products/alexa-fluor-700-anti-mouse-cd4-antibody-3385?GroupID=BLG4745

CD4 Brillant Violet 605 Mouse RM4-5 BioLegend 100548 1:100 https://www.biolegend.com/ja-jp/products/brilliant-violet-605-anti-mouse-cd4-antibody-7627?GroupID=BLG4745

CD4 Brillant Violet 711 Mouse RM4-5 Biolegend 100550 1:100 https://www.biolegend.com/en-us/products/brilliant-violet-711-anti-mouse-cd4-antibody-7925?GroupID=BLG4211

CD4 PE Human/Mouse RM4-5 BioLegend 100512 1:100 https://www.biolegend.com/en-us/products/pe-anti-mouse-cd4-antibody-482

CD44 Alexa Fluor 488 Human/Mouse IM7 BioLegend 103016 1:100 https://www.biolegend.com/fr-ch/explore-new-products/alexa-fluor-488-anti-mouse-human-cd44-antibody-3097?GroupID=BLG10248

CD44 APC Human/Mouse IM7 BioLegend 103012 1:200 https://www.biolegend.com/en-us/products/apc-anti-mouse-human-cd44-antibody-312

CD44 PerCP-Cy 5.5 Mouse IM7 BioLegend 103032 1:100 https://www.biolegend.com/en-us/search-results/percp-cyanine5-5-anti-mouse-human-cd44-antibody-5605?GroupID=BLG10248

CD45 APC Mouse 30-F11 eBioscience 17-0451-82 1:100 https://www.thermofisher.com/antibody/product/CD45-Antibody-clone-30-F11-Monoclonal/17-0451-82#:~:text=17%2D0451%2D83%20was%20used,diagnosis%20and%20prognosis%20of%20mesothelioma.

CD45 APC Mouse 30-F11 BioLegend 103112 1:100 https://www.biolegend.com/en-ie/products/apc-anti-mouse-cd45-antibody-97

CD45 Brillant Violet 510 Mouse 30-F11 BioLegend 103138 1:400 https://www.biolegend.com/fr-lu/cell-health/brilliant-violet-510-anti-mouse-cd45-antibody-7995

CD45 Pacific Blue Mouse 30-F11 BioLegend 103126 1:100 https://www.biolegend.com/en-gb/search-results/pacific-blue-anti-mouse-cd45-antibody-3102?GroupID=BLG6841

CD45 PE Mouse 30-F11 BioLegend 103106 1:100 https://www.biolegend.com/en-us/products/pe-anti-mouse-cd45-antibody-100

CD45.1 FITC Mouse A20 Invitrogen 11-0453-82 1:100 https://www.thermofisher.com/antibody/product/CD45-1-Antibody-clone-A20-Monoclonal/11-0453-82

CD45.1 PE Mouse A20 Invitrogen 12-0453-82 1:100 https://www.thermofisher.com/antibody/product/CD45-1-Antibody-clone-A20-Monoclonal/12-0453-82

CD45.1 PE-Cy7 Mouse 104 BioLegend 110730 1:100 https://www.biolegend.com/fr-fr/products/pe-cyanine7-anti-mouse-cd45-1-antibody-4917?GroupID=BLG1933

CD45.2 APC Mouse 104 eBioscience 17-0454-82 1:100 https://www.thermofisher.com/antibody/product/CD45-2-Antibody-clone-104-Monoclonal/17-0454-82

CD45.2 APC-Cy7 Mouse 104 BioLegend 109824 1:100 https://www.biolegend.com/nl-nl/products/apc-cyanine7-anti-mouse-cd45-2-antibody-3906

CD45.2 APC-eFluor 780 Mouse 104 eBioscience 47-0454-82 1:100 https://www.thermofisher.com/antibody/product/CD45-2-Antibody-clone-104-Monoclonal/47-0454-82

CD45.2 Pacific Blue Mouse 104 eBioscience 109820 1:100 https://www.biolegend.com/en-us/products/pacific-blue-anti-mouse-cd45-2-antibody-3108?GroupID=BLG1934

CD45.2 PE Mouse 104 Invitrogen 12-0454-82 1:100 https://www.thermofisher.com/antibody/product/CD45-2-Antibody-clone-104-Monoclonal/12-0454-82

CD45R Brillant Violet 605 Mouse RA3-6B2 BioLegend 103244 1:100 https://www.biolegend.com/en-us/products/brilliant-violet-605-anti-mouse-human-cd45r-b220-antibody-7870?GroupID=GROUP20

CD45RA BUV737 Human HI100 BD Biosciences 612846 1:400 https://www.bdbiosciences.com/en-us/products/reagents/flow-cytometry-reagents/research-reagents/single-color-antibodies-ruo/buv737-mouse-anti-human-cd45ra.612846

CD62L Alexa Fluor 488 Mouse MEL-14 BioLegend " 104420" 1:100 https://www.biolegend.com/fr-fr/products/alexa-fluor-488-anti-mouse-cd62l-antibody-3115?GroupID=GROUP20

CD62L PE-Cy7 Mouse MEL-14 BioLegend 104418 1:100 https://www.biolegend.com/en-us/products/pe-cyanine7-anti-mouse-cd62l-antibody-1922?GroupID=BLG10670

CD62L PE/Dazzle Mouse MEL-14 BioLegend 104448 1:200 https://www.biolegend.com/de-de/products/pe-dazzle-594-anti-mouse-cd62l-antibody-12137

CD64 Brilliant Violet 711 Mouse X54-5/7.1 BioLegend 139311 1:100 https://www.biolegend.com/fr-ch/products/brilliant-violet-711-anti-mouse-cd64-fcgammari-antibody-9920?GroupID=BLG8805

CD66b FITC Human G10F5 BioLegend 305103 1:400 https://www.biolegend.com/en-us/products/fitc-anti-human-cd66b-antibody-666

CD66b PE Human G10F5 BioLegend 305106 1:400 https://www.biolegend.com/en-us/products/pe-anti-human-cd66b-antibody-6529

CD86 APC-Cy7 Mouse GL-1 BioLegend 553030 1:100 https://www.biolegend.com/en-us/products/apc-cyanine7-anti-mouse-cd86-antibody-6554?GroupID=BLG11928

CD86 Pacific Blue Human IT2.2 BioLegend 305417 1:100 https://www.biolegend.com/en-us/products/pacific-blue-anti-human-cd86-antibody-3357

CD86 Pacific Blue Mouse GL-1 BioLegend 105022 1:100 https://www.biolegend.com/en-us/products/pacific-blue-anti-mouse-cd86-antibody-3122

CD8a PerCp-Cy5.5 Mouse 53-6.7 BioLegend 100734 1:400 https://www.biolegend.com/nl-be/products/percp-cyanine5-5-anti-mouse-cd8a-antibody-4255

CD8a Alexa Fluor 700 Mouse 53-6.7 eBioscience 56-0081-82 1:100 https://www.thermofisher.com/antibody/product/CD8a-Antibody-clone-53-6-7-Monoclonal/56-0081-82

CD8a APC-Cy7 Mouse 53-6.7 BioLegend 100714 1:100 https://www.biolegend.com/en-gb/diagnostic-1/apc-cyanine7-anti-mouse-cd8a-antibody-2269

CD8a Briliant Violet 605 Mouse 53-6.7 BioLegend 100744 1:100 https://www.biolegend.com/en-us/productstab/brilliant-violet-605-anti-mouse-cd8a-antibody-7636

CD8a Brillant Violet 510 Mouse 53-6.7 BioLegend 100752 1:100 https://www.biolegend.com/en-ie/cell-health/brilliant-violet-510-anti-mouse-cd8a-antibody-7992

CD8a FITC Mouse 53-6.7 Invitrogen 553030 1:100 https://www.bdbiosciences.com/en-ch/products/reagents/flow-cytometry-reagents/research-reagents/single-color-antibodies-ruo/fitc-rat-anti-mouse-cd8a.553030

CD8a PE Mouse 53-6.7 Invitrogen 12-0081-82 1:100 https://www.thermofisher.com/antibody/product/CD8a-Antibody-clone-53-6-7-Monoclonal/12-0081-82

CD8a PerCP-Cy 5.5 Human SK1 BioLegend 353803 1:400 https://www.biolegend.com/en-us/products/percp-cyanine5-5-anti-human-cd8-antibody-6389
CLEC9A APC Human 8F9 BioLegend 353806 1:200 https://www.biolegend.com/en-us/products/apc-anti-human-cd370-clec9a-dngr1-antibody-11714

F4/80 Alexa Fluor 488 Mouse BM8 BioLegend 123120 1:100 https://www.biolegend.com/en-us/products/alexa-fluor-488-anti-mouse-f4-80-antibody-4073

F4/80 APC Mouse BM8 BioLegend 123116 1:100 https://www.biolegend.com/en-us/products/apc-anti-mouse-f4-80-antibody-4071

F4/80 APC-Cy7 Mouse BM8 BioLegend 123118 1:100 https://www.biolegend.com/en-us/products/apc-cyanine7-anti-mouse-f4-80-antibody-4072

F4/80 PE-Cy7 Mouse BM8 BioLegend 123114 1:200 https://www.biolegend.com/en-us/productstab/pe-cyanine7-anti-mouse-f4-80-antibody-4070?GroupID=BLG5319

Granzyme B PE-Texas Red Human/Mouse GB11 ThermoFisher Scientific GRB17 1:100 https://www.thermofisher.com/antibody/product/Granzyme-B-Antibody-clone-GB11-Monoclonal/GRB17

GD2 APC Mouse/Human 14G2A BioLegend 357305 1:100. https://www.biolegend.com/fr-ch/products/apc-anti-human-ganglioside-gd2-antibody-9298

HLA-A/B/C PerCp-Cy5.5 Human W6/32 BioLegend 311419 1:400 https://www.biolegend.com/en-us/products/percp-cyanine5-5-anti-human-hla-a-b-c-antibody-7057

HLA-A2 PE-Cy7 Human BB7.2 BioLegend 343313 1:100 https://www.biolegend.com/en-us/products/pe-cyanine7-anti-human-hla-a2-antibody-8184

HLA-DR PerCp-Cy5.5 Human L243 BioLegend 307629 1:200 https://www.biolegend.com/en-us/products/percp-cyanine5-5-anti-human-hla-dr-antibody-4246

IFN-γ APC Mouse XMG1.2 BD Biosciences 562018 1:100 https://www.bdbiosciences.com/en-au/products/reagents/flow-cytometry-reagents/research-reagents/single-color-antibodies-ruo/apc-rat-anti-mouse-ifn.562018

IFN-γ APC Mouse XMG1.2 BioLegend 505810 1:200 https://www.biolegend.com/en-us/products/apc-anti-mouse-ifn-gamma-antibody-993?GroupID=GROUP24

IFN-γ PE-Cy7 Human 4S.B3 BD Biosciences 561036 1:200 https://www.bdbiosciences.com/en-us/products/reagents/flow-cytometry-reagents/research-reagents/single-color-antibodies-ruo/pe-cy-7-mouse-anti-human-ifn.561036

ISO PE-Cy7 Human MPC-11 BioLegend 400325 1:50 https://www.biolegend.com/en-us/products/pe-cyanine7-mouse-igg2b-kappa-isotype-ctrl-1928

ISO PerCp-Cy5.5 Human MOPC-173 BioLegend 400257 1:800 https://www.biolegend.com/en-us/products/percp-cyanine5-5-mouse-igg2a-kappa-isotype-ctrl-4207

Ly6C Alexa Fluor 700 Mouse HK1.4 BioLegend 128024 1:100 https://www.biolegend.com/en-us/products/alexa-fluor-700-anti-mouse-ly-6c-antibody-6757

Ly6C Briliant Violet 605 Mouse HK1.4 BioLegend 128035 1:100 https://www.biolegend.com/en-us/products/brilliant-violet-605-anti-mouse-ly-6c-antibody-8727

Ly6C Briliant Violet 711 Mouse HK1.4 BioLegend 128037 1:100 https://www.biolegend.com/en-us/products/brilliant-violet-711-anti-mouse-ly-6c-antibody-8935

Ly6C Brillant Violet 650 Mouse HK1.4 BioLegend 128049 1:200 https://www.biolegend.com/en-us/products/brilliant-violet-650-anti-mouse-ly-6c-antibody-17378

Ly6G Briliant Violet 605 Mouse 1A8 BioLegend " 127639" 1:100 https://www.biolegend.com/en-us/products/brilliant-violet-605-anti-mouse-ly-6g-antibody-12244

Ly6G Pacific Blue Mouse 1A8 BioLegend 127611 1:100 https://www.biolegend.com/en-us/products/pacific-blue-anti-mouse-ly-6g-antibody-6082

MHC class II APC-Cy7 Mouse M5/114.15.2 BioLegend 107628 1:100 https://www.biolegend.com/en-us/products/apc-cyanine7-anti-mouse-i-a-i-e-antibody-5966

MHC class II FITC Mouse M5/114.15.2 Invitrogen 11-5321-85 1:100 https://www.thermofisher.com/antibody/product/MHC-Class-II-I-A-I-E-Antibody-clone-M5-114-15-2-Monoclonal/11-5321-82

MHC class II Pacific Blue Mouse M5/114.15.2 BioLegend 107620 1:100 https://www.biolegend.com/en-us/products/pacific-blue-anti-mouse-i-a-i-e-antibody-3136?GroupID=BLG11931

MHC class II PE Mouse M5/114.15.2 ThermoFisher Scientific A14763 1:100 https://www.thermofisher.com/antibody/product/I-A-I-E-Antibody-clone-M5-114-15-2-Monoclonal/A14763

MHC class II Pe-Cy5 Mouse M5/114.15.2 BioLegend 107612 1:100 https://www.biolegend.com/de-at/products/pe-cyanine5-anti-mouse-i-a-i-e-antibody-2487?GroupID=BLG11931

MHC class II PE-Cy7 Mouse M5/114.15.2 BioLegend 107629 1:100 https://www.biolegend.com/fr-fr/products/pe-cyanine7-anti-mouse-i-a-i-e-antibody-6136

MHC class II PerCP-Cy 5.5 Mouse M5/114.15.2 BioLegend 107625 1:200 https://www.biolegend.com/nl-nl/products/percp-cyanine5-5-anti-mouse-i-a-i-e-antibody-4282

NK 1.1 AF488 Mouse PK136 BioLegend 108706 1:200 https://www.biolegend.com/en-us/products/fitc-anti-mouse-nk-1-1-antibody-429

NK 1.1 Brillant Violet 711 Mouse PK136 BioLegend 108745 1:200 https://www.biolegend.com/en-us/products/brilliant-violet-711-anti-mouse-nk-1-1-antibody-9576

NK1.1 PE-Cy7 Mouse PK136 BioLegend 108714 1:100 https://www.biolegend.com/en-us/soluble-mhc/pe-cyanine7-anti-mouse-nk-1-1-antibody-2840

TCRβ Brillant Violet 650 Mouse H57-597 BioLegend 109251 1:400 https://www.biolegend.com/en-us/products/brilliant-violet-650-anti-mouse-tcr-beta-chain-antibody-17652

TIM-3 Briliant Violet 711 Mouse RMT3-23 BioLegend 119727 1:100 https://www.biolegend.com/en-gb/products/brilliant-violet-711-anti-mouse-cd366-tim-3-antibody-14918?GroupID=BLG10656

TNFa A700 Mouse MAb11 BD Biosciences 561023 1:100 https://www.bdbiosciences.com/en-ch/products/reagents/flow-cytometry-reagents/research-reagents/single-color-antibodies-ruo/alexa-fluor-700-mouse-anti-human-tnf.561023

TNFa PE-Cy7 Mouse MP6-XT22 BioLegend 506324 1:100 https://www.biolegend.com/de-at/products/pe-cyanine7-anti-mouse-tnf-alpha-antibody-5866?GroupID=GROUP24

TNFa PE/Dazzle Human MAb11 BD Biosciences 561023 1:100 https://www.bdbiosciences.com/en-us/products/reagents/flow-cytometry-reagents/research-reagents/single-color-antibodies-ruo/alexa-fluor-700-mouse-anti-human-tnf.561023

XCR1 Brillant Violet 510 Mouse/Rat ZET BioLegend 148218 1:100 https://www.biolegend.com/fr-lu/products/brilliant-violet-510-anti-mouse-rat-xcr1-antibody-10751

XCR1 PE Mouse ZET BioLegend 148204 1:200 https://www.biolegend.com/en-us/clone-search/pe-anti-mouse-rat-xcr1-antibody-10217?GroupID=GROUP20

β2-microglobulin PE Mouse A16041A BioLegend 154503 1:100 https://www.biolegend.com/fr-ch/products/pe-anti-mouse-beta2-microglobulin-antibody-15126?GroupID=ImportedGROUP1

F4/80 PE Mouse BM8 BioLegend 123109 1:50 https://www.biolegend.com/en-us/products/pe-anti-mouse-f4-80-antibody-4068

CD8a APC Mouse 53-6.7 BD Bioscience 561093 1:50 https://www.bdbiosciences.com/en-us/products/reagents/flow-cytometry-reagents/research-reagents/single-color-antibodies-ruo/apc-rat-anti-mouse-cd8a.561093

CD8b APC Mouse YTS156.7.7 BioLegend 126613 1:50 https://www.biolegend.com/en-us/products/apc-anti-mouse-cd8b-antibody-9055

CD3e PE Mouse OKT3 BioLegend 317308 1:100 https://www.biolegend.com/en-us/products/pe-anti-human-cd3-antibody-3645

CD3 PE Mouse 500A2 BD Bioscience 553240 1:50 https://www.bdbiosciences.com/en-us/products/reagents/flow-cytometry-reagents/research-reagents/single-color-antibodies-ruo/pe-hamster-anti-mouse-cd3e.553240

NKp46 PE Mouse 29A1.4 BioLegend 137603 1:50 https://www.biolegend.com/en-us/products/pe-anti-mouse-cd335-nkp46-antibody-6523

GFP Unconj. GFP polyclonal Proteintech 50430-2-AP 1:200 https://www.ptglab.com/products/eGFP-Antibody-50430-2-AP.htm
CD31 Unconj. Mouse polyclonal LifeSpan Biosciences LS-C150165 1:300 https://www.lsbio.com/targets/target/g3760?removedcatalog=LS-C150165&prefix=a

Secondary (for GFP) AF488 Rabbit polyclonal ThermoFisher A-21206 1:500 https://www.thermofisher.com/antibody/product/Donkey-anti-Rabbit-IgG-H-L-Highly-Cross-Adsorbed-Secondary-Antibody-Polyclonal/A-21206

Secondary (for CD31) AF488 Goat polyclonal ThermoFisher A-11055 1:500 https://www.thermofisher.com/antibody/product/Donkey-anti-Goat-IgG-H-L-Cross-Adsorbed-Secondary-Antibody-Polyclonal/A-11055

CD8a Unconj. Mouse H2(20) Cell signaling 98941S 1:400 https://www.cellsignal.com/products/primary-antibodies/cd8a-d4w2z-xp-rabbit-mab/98941?_requestid=429297&gclid=CjwKCAjwjaWoBhAmEiwAXz8DBaAmzYX7MryLyEhP4yt8IldLyCsvig3c8OP2gyoJ_IJs0ysTRg0TXBoC6g0QAvD_BwE&gclsrc=aw.ds&_requestid=2408425

CD4 Unconj. Mouse 14-9766 eBioscience 14-9766 1:1000 https://www.thermofisher.com/antibody/product/CD4-Antibody-clone-4SM95-Monoclonal/14-9766-82

Secondary (for CD8a) HRP* Rabbit polyclonal Agilent DAKO P044801-2 1:300 https://www.agilent.com/en/product/specific-proteins/elisa-kits-accessories/goat-anti-rabbit-immunoglobulins-hrp-affinity-isolated-2717113

Secondary (for CD4) HRP** Rat polyclonal Jackson Immuno Research 312-005-045 1:1000 https://www.jacksonimmuno.com/catalog/products/312-005-045

* HRP signal was revealed by Opal 620 reagent pack (FP1495001KT). https://my.akoyabio.com/ccrz__ProductDetails?sku=FP1495001KT&cclcl=en_US

** HRP signal was revealed by Opal 540 reagent pack (FP1494001KT). https://my.akoyabio.com/ccrz__ProductDetails?sku=FP1494001KT&cclcl=en_US

Eukaryotic cell lines

Policy information about cell lines and Sex and Gender in Research

Cell line source(s)

293T cells were obtained from Dr. Luigi Naldini (San Raffaele Institute, Milan, Italy) and maintained in the De Palma laboratory. Phoenix-Eco cells were obtained from ATCC (cat. CRL-3214) and maintained in the Migliorini laboratory. MC38 colorectal carcinoma cells and B16F10 melanoma cells modified to express OVA were obtained from Pedro Romero (University of Lausanne, Switzerland). SB28 glioma cells available in the Migliorini laboratory were generated by Hideho Okada (UCSF, CA); these cells express both luciferase and GFP.

Authentication

While the original stocks of cancer cell lines were authenticated, we did not perform further authentication in the past several years. However, the cell lines appeared authentic based on their morphology and growth behavior in vitro and in vivo, especially with respect to ability to form tumors in mice, expression of defined fluorescent or bioluminescent genes, and gene expression. In particular, 293T and Phoenix-Eco cells supported high titer LV and RV production; B16F10 cells were validated as melanoma cells by RNA-Seq analysis; and SB28 cells expressed luciferase and GFP and formed invasive gliomas in mice.

Mycoplasma contamination

All cell lines have tested negative for Mycoplasma contamination in tests routinely performed in the laboratory.

Commonly misidentified lines
(See ICLAC register)

None

Animals and other research organisms

Policy information about studies involving animals; ARRIVE guidelines recommended for reporting animal research, and Sex and Gender in Research

Laboratory animals

All studies used C57Bl/6 mice (CD45.2 wild-type; CD45.1 wild-type; Batf3–/–; Rag1–/–). C57Bl/6 (CD45.2) mice were purchased from Charles River Laboratories (France). C57Bl/6 (CD45.1), Batf3–/– and Rag1–/– mice were maintained as stable colonies in the EPFL mouse facility. All mice were housed in groups of up to 5 mice/cage at 18-24 degrees C (ambient temperature), with 40-60% humidity, and maintained on a 12 h light/dark cycle (6 am to 6 pm). Food and water were available ad libitum. Experiments involving subcutaneous tumor models used cohorts of 6-9 weeks old female mice. Glioma studies were performed twice, once in female and once in male C57Bl/6 mice (all 6-8 weeks old). The liver cancer studies used 8-9 weeks old male C57Bl/6 mice. BM cells were isolated from female mice. The mice with subcutaneous tumors were monitored 3 times per week. The tumors were allowed to grow up to 1 cm3 in size. Upon reaching the endpoint tumor size, the experiments could continue for additional 48h provided that all health parameters detailed in a health score sheet remained normal. Long (D) and short (d) tumor diameters were measured with a caliper and the tumor volume calculated using the following formula: tumor volume = ½ x d2 x D. Termination criteria in the liver tumor models were defined by authority-confined endpoints (morbidity; non-physiological posture; indication of jaundice, cramps, or emaciation; weight loss of more than 20% of the initial weight). Termination criteria in the intracranial glioma model were defined by authority-confined endpoints (15% weight loss over one week; compromised ability to walk, eat or drink; dyspnea; hunched posture; or lethargic behavior).

Wild animals

The study did not involve wild animals.

Reporting on sex

Experiments involving subcutaneous tumor models used female mice. Glioma studies were performed twice, once in female and once in male mice. The liver cancer studies used male mice.

Field-collected samples

None

Ethics oversight	The studies conducted at EPFL (De Palma's laboratory) were performed according to protocols approved by the Veterinary Authorities of the Canton Vaud according to Swiss law (protocols VD3154, VD3154.1, VD3785). The studies conducted at UNIGE (Migliorini's laboratory; brain glioma model) were performed according to protocols approved by the Veterinary Authorities of the Canton Geneva according to Swiss law (protocol VD3717c). The studies conducted at DKFZ (Heikenwälder's laboratory; liver cancer models) were performed according to protocols approved by the Regierungspräsidium Karlsruhe according to German law (protocols G275/18, G5/19 and DKFZ332). All studies were compliant with the humane endpoints established in the above authorizations.

Note that full information on the approval of the study protocol must also be provided in the manuscript.

Flow Cytometry

Plots

Confirm that:

☒ The axis labels state the marker and fluorochrome used (e.g. CD4-FITC).

☒ The axis scales are clearly visible. Include numbers along axes only for bottom left plot of group (a 'group' is an analysis of identical markers).

☒ All plots are contour plots with outliers or pseudocolor plots.

☒ A numerical value for number of cells or percentage (with statistics) is provided.

Methodology

Sample preparation

Processing of subcutaneous tumors for flow cytometry
Tumors were excised, chopped, and enzymatically digested with collagenase IV (0.2 mg/ml; Worthington), dispase (2 mg/ml; Life Technologies), and DNaseI (0.1 mg/ml; New England BioLabs), in DMEM medium, for 30 min at 37°C. Filtered single cells were washed with PBS and transferred to 96 u-bottom wells for staining with flow cytometry antibodies.

Processing of hematopoietic and lymphoid organs for flow cytometry
Spleens, tumor- or liver-draining lymph nodes (tdLNs and ldLNs, respectively) were smashed gently and thoroughly on a 70 µm cell strainer and RBCs were depleted RBC lysis buffer (Sigma). Single-cell suspensions were washed in PBS and transferred to 96 u-bottom wells for staining with antibodies.

Processing of liver and lung tissue for flow cytometry
Liver cells were isolated by mechanical dissociation followed by enzymatic digestion for 40 min at 37°C in RPMI medium with collagen IV (final concentration: 60 U) and DNase I (final concentration: 25 µg/ml). After washing in PBS, leukocytes were isolated by Percoll (Sigma-Aldrich; GE17-0891-01) gradient (40-80% Percoll/HBSS) centrifugation for 15 min at 1,800xg at 4°C. Enriched leukocytes were collected, washed in FACS buffer, and counted (CountBright™ Absolute Counting Beads; Thermo Fisher; C36950) before staining with antibodies. Lungs were harvested and digested with the mouse lung dissociation kit (Miltenyi Biotec) using gentleMACS tissue dissociator (Miltenyi Biotec). RBCs were lysed with RBC lysis buffer (Sigma) before staining with antibodies.

Ex vivo restimulation of cells for flow cytometry
For ex vivo re-stimulation of immune cells, cells were re-stimulated with phorbol 12-myristate 13-acetate (PMA; 10 ng/ml; Sigma-Aldrich; P2696) and ionomycin (500 ng/ml; Sigma-Aldrich; I0634) in RPMI medium for 30 minutes, followed by Golgi Stop (BD Biosciences; Cat No. 554715) and Golgi plug (BD Biosciences; Cat No. 555028) for 3h. Cells were then stained for intracellular cytokines (BD Biosciences; Cat No. 554715). Liver-derived cells were incubated for 2h in RPMI medium containing the "cell activation cocktail" with brefeldin A (1:500; Biolegend; 423304) and monensin solution (1:1,000; Biolegend; 420701), before flow cytometry analysis.

Instrument

BD LSRFortessa™, BD® LSR II SORP, and Invitrogen Attune™ NxT were used for acquisition of flow cytometry data.

Software

For flow cytomentry analysis, we employed FlowJo, LLC V10.1.

Cell population abundance

Cell sorting used a BD FACSAria™ Fusion Flow Cytometer. Enriched cell populations were used for functional assays or tumor growth and not for analytical studies.

Gating strategy

Gating strategies are shown in Supplementary Figures 1-7.

MOUSE CELLS:

Spleen:
- cDCs: CD11chighMHCII+
- cDC1: CD11chighMHCII+CD8a+CD11b–
- cDC2: CD11chighMHCII+CD8a¬–CD11b+
- Double-negative (DN) cDCs: CD11chighMHCII+CD8a–CD11b–
- CD8+ T-effector: CD3+CD8+CD44+CD62L–
- CD4+ T-effector: CD3+CD4+CD44+CD62L–

Tumor:
- cDCs: Ly6C–F4/80–CD11c+MHCII+
- cDC1: Ly6C–F4/80–CD11c+MHCII+CD103+CD11b–; Ly6C–F4/80–CD11c+MHCII+XCR1+CD11b–
- cDC2: Ly6C–F4/80–CD11c+MHCII+CD103–CD11b+; Ly6C–F4/80–CD11c+MHCII+XCR1–CD11b+
- Macrophages: CD11b+F4/80+

- M1-like macrophages: CD11b+F4/80+MHCII+; CD11b+F4/80+MHCII+CD86+
- CD8+ T: CD3+CD8+; CD11b–CD3+CD8+CD4–; NK1.1–CD3+CD8+CD4–
- CD4+ T: CD3+CD4+; CD11b–CD3+CD8–CD4+
- NK: CD11b–NK1.1+; CD3–NK1.1+
- NKT: CD3+NK1.1+
- Neutrophils: Ly6C+Ly6G+

TdLN:
- cDCs: CD11c+/highMHCII+
- cDC1: CD11c+/highMHCII+CD8a+CD11b–
- cDC2: CD11c+/high MHCII+CD8a¬–CD11b+
- CD8+ T-effector: CD3+CD8+CD44+CD62L–; MHCII–CD3+CD8+CD44+CD62L–
- CD4+ T-effector: CD3+CD4+CD44+CD62L–; MHCII–CD3+CD4+CD44+CD62L–

Lung
- cDCs: Ly6C–F4/80–CD11c+MHCII+
- cDC1: Ly6C–F4/80–CD11c+MHCII+CD103+CD11b–
- cDC2: Ly6C–F4/80–CD11c+MHCII+CD103–CD11b+
- Macrophages: CD11b+F4/80+

Liver:
- NK: CD45+TCRβ–NK1.1+
- cDCs: CD45+CD11b+F4/80–CD11c+MHCII+
- Monocytes: CD45+CD11b+F4/80–CD11c–
- Kupffer cells: CD45+CD11b+F4/80+
- CD8+ T: CD45+TCRβ+NK1.1–CD8+
- CD4+ T: CD45+TCRβ+NK1.1–CD4+
- IFNγ+ CD8+ T: CD45+TCRβ+NK1.1–CD8+IFNγ+
- CD8+ T-effector: CD45+TCRβ+NK1.1–CD8+CD44+CD62L–
- CD4+ T-effector: CD45+TCRβ+NK1.1–CD4+CD44+CD62L–

Spleen and ldLN (liver cancer models):
- CD8+ T-effector: CD45+TCRβ+NK1.1–CD8+CD44+CD62L–
- CD4+ T-effector: CD45+TCRβ+NK1.1–CD4+CD44+CD62L–

HUMAN CELLS:

Human hematopoietic progenitors (day 7 cultures):
- CMP (Lin–CD34+CD115–CD1c–CD303–CD141–CD123–CD117+CD135+CD116–CD45RA–)
- GMDP (Lin–CD34+CD115–CD1c–CD303–CD141–CD123–CD117+CD135+CD116–CD45RA+)
- MDP (Lin–CD34+CD115+CD1c–CD303–CD141–CD123–CD117+CD135+CD116–CD45RA+)
- CDP (Lin–CD34+CD115–CD1c–CD303–CD141–CD123+CD117+CD135+CD116+CD45RA+)
- Pre-DC (Lin–CD34–CD115–CD1c–CD303–CD141–CD123–CD117+CD135+CD116+CD45RA+)

Human cells (day-14 cultures):

APCs
- Monocytes: CD66b–CD3–CD19–CD14+
- cDC1: CD66b–CD3–CD19–CD14–CD141+CLEC9A+
- Immature DCs: CD66b–CD3–CD19–CD14–CD141+CLEC9A–
- cDC2: CD66b–CD3–CD19–CD14–CD141–CLEC9A–CD1c+

Other cells
- Granulocytes: CD66b+
- T cells: CD66b–CD3+
- B cells: CD66b– CD3–CD19+
- Other cells: CD66b–CD3–CD19–CD14–CD141–CLEC9A–CD1c–

☒ Tick this box to confirm that a figure exemplifying the gating strategy is provided in the Supplementary Information.
